# Bacillibactin and Bacillomycin Analogues with Cytotoxicities against Human Cancer Cell Lines from Marine *Bacillus* sp. PKU-MA00093 and PKU-MA00092

**DOI:** 10.3390/md16010022

**Published:** 2018-01-10

**Authors:** Mengjie Zhou, Fawang Liu, Xiaoyan Yang, Jing Jin, Xin Dong, Ke-Wu Zeng, Dong Liu, Yingtao Zhang, Ming Ma, Donghui Yang

**Affiliations:** State Key Laboratory of Natural and Biomimetic Drugs, Department of Natural Medicines, School of Pharmaceutical Sciences, Peking University, 38 Xueyuan Road, Haidian District, Beijing 100191, China; zmj216@bjmu.edu.cn (M.Z.); fawang90@126.com (F.L.); yaner1888@163.com (X.Y.); jinjing@bjmu.edu.cn (J.J.); lingyu213@sohu.com (X.D.); ZKW@bjmu.edu.cn (K.-W.Z.); liudong_1982@126.com (D.L.); zytao1988@163.com (Y.Z.)

**Keywords:** bacillibactin, bacillomycin, genome mining, marine *Bacillus*, nonribosomal peptides

## Abstract

Nonribosomal peptides from marine *Bacillus* strains have received considerable attention for their complex structures and potent bioactivities. In this study, we carried out PCR-based genome mining for potential nonribosomal peptides producers from our marine bacterial library. Twenty-one “positive” strains were screened out from 180 marine bacterial strains, and subsequent small-scale fermentation, HPLC and phylogenetic analysis afforded *Bacillus* sp. PKU-MA00092 and PKU-MA00093 as two candidates for large-scale fermentation and isolation. Ten nonribosomal peptides, including four bacillibactin analogues (**1**–**4**) and six bacillomycin D analogues (**5**–**10**) were discovered from *Bacillus* sp. PKU-MA00093 and PKU-MA00092, respectively. Compounds **1** and **2** are two new compounds and the ^1^H NMR and ^13^C NMR data of compounds **7** and **9** is first provided. All compounds **1**–**10** were assayed for their cytotoxicities against human cancer cell lines HepG2 and MCF7, and the bacillomycin D analogues **7**–**10** showed moderate cytotoxicities with IC_50_ values from 2.9 ± 0.1 to 8.2 ± 0.2 µM. The discovery of **5**–**10** with different fatty acid moieties gave us the opportunity to reveal the structure-activity relationships of bacillomycin analogues against these human cancer cell lines. These results enrich the structural diversity and bioactivity properties of nonribosomal peptides from marine *Bacillus* strains.

## 1. Introduction

Nonribosomal peptides produced by nonribosomal peptide synthetases (NRPS) from *Bacillus* strains are important sources of clinically used agents or bioactive molecules. Bacitracin, one mixture of nonribosomal peptides discovered from *Bacillus subtilis*, is used as an antibacterial drug, with the mode of action of interfering with cell wall and peptidoglycan synthesis [1]. Tyrocidines and gramicidins, nonribosomal peptides discovered from *Bacillus brevis* strains, show antibacterial activities [2,3], and their early biosynthetic research initiate the characterization of the functions of domains in NRPS assembly lines [4,5,6,7,8]. Bacillibactin, one nonribosomal peptide discovered from *Bacillus subtilis*, serves as a catecholic siderophore in iron acquisition that is essential to the host’s life [9,10]. Other important nonribosomal peptides produced by *Bacillus* strains include surfactin, fengycin, and iturin, which represent three families of lipopeptides showing antibacterial, antifungal, and hemolytic activities owing to their cell membrane-interacting properties [11,12]. In last two decades, marine *Bacillus* strains as nonribosomal peptide producers have received considerable attention [13]. The marine environments characterized by high salinity, high pressure, and low temperature hold the promise of producing new natural products structurally or bioactively distinct with those from terrestrial environments. Various *Bacillus* species have been isolated from marine sources with different isolation methods [14,15,16], and new nonribosomal peptides with varied bioactivities have been discovered. Gageopeptides that were discovered from *Bacillus subtilis* collected from Gageocho reef, Republic of Korea were linear lipopeptides showing potent antifungal and antibacterial activities [17]. Bogorol A discovered from *Bacillus* sp. collected from Papua New Guinea was first linear “cationic peptide antibiotics”, showing potent antibacterial activity [18]. Bacillistatins discovered from *Bacillus silvestris* collected from southern Chile ocean were cyclodepsipeptides showing potent antitumor activity [19]. More nonribosomal peptides with different bioactivities were summarized in Figure S1, depicting the potential of nonribosomal peptides from marine *Bacillus* strains as natural drug leads.

**Figure 1:**
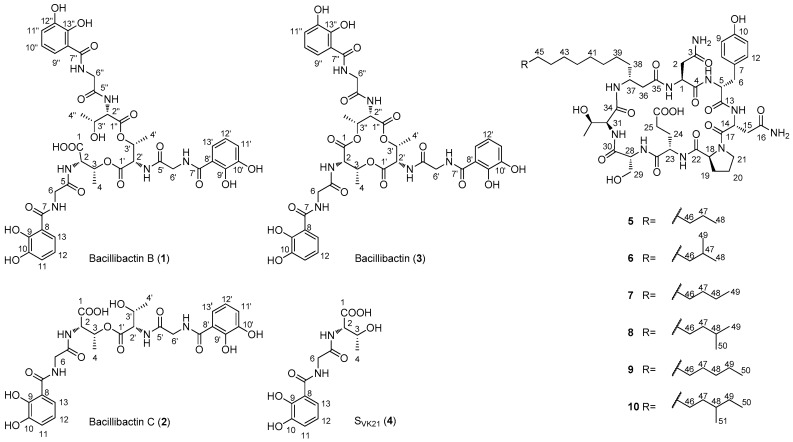
The structures of bacillibactin analogues (**1**–**4**) and bacillomycin D analogues (**5**–**10**) discovered from *Bacillus* sp. PKU-MA00093 and PKU-MA00092, respectively.

Genome mining targeting the NRPS genes is a useful tool for the discovery of new nonribosomal peptides. The investigation of sequenced genomic DNA based on bioinformatics analysis has been extensively applied to identify a specific nonribosomal peptide gene cluster and its corresponding natural product [20,21,22]. The PCR screening using degenerate primers with unsequenced genomic DNAs as the templates has been applied to discover 13 NRPS-containing *Bacillus* strains from 109 marine bacteria collection [14] and grisechelin-type nonribosomal peptides from an actinomycetes collection [23]. We here report the discovery of 10 nonribosomal peptides (**1**–**10**), including two new compounds (**1** and **2**) from our marine bacteria library (Figure 1), based on the PCR screening with degenerate primers designed from the conserved sequences of adenylation domains (A domain) in six nonribosomal peptides biosynthesis [24]. All the compounds **1**–**10** were assayed for their cytotoxicities against human cancer cell lines HepG2 and MCF-7, and compound **8** showed best cytotoxicities with IC_50_ values of 2.9 ± 0.1 µM. These results highlight marine *Bacillus* strains as the rich sources of nonribosomal peptides and the power of genome mining in natural product discovery.

## 2. Results and Discussion

### 2.1. Genome Mining for Nonribosomal Peptides Discovery from Marine Bacteria Library Based on a PCR Screening Method

We have constructed a marine bacteria library containing strains isolated from sponges, corals and deposits collected from South China Sea and south coasts of China. To discover nonribosomal peptides from this library, we carried out a PCR screening targeting adenylation domains (A domain) in nonribosomal peptides biosynthesis with genomic DNAs of strains in the library as the templates, using the degenerate primers (A3F and A7R) that were designed from the conserved sequences of A domains in cephamycin, vancomycin, balhimycin, actinomycin, pristinamycin, and chloroeremomycin biosynthesis [24]. Totally 180 strains in the library were screened and 21 strains were identified as the “positive hits”, which gave clear PCR products with expected size of ~700 bp based on the agarose gel electrophoresis analysis (Figure S2). All 21 PCR products were sequenced and confirmed to encode A domains with high sequence identities to known NRPS homologues (Table S1). The high sequence identities (82–99%, Table S1) to known NRPS homologues confirmed our PCR screening’s accuracy, but excluded the possibility of new A domain sequences discovered. The same degenerate primers (A3F and A7R) have been used in the screening of NRPS genes from marine Actinobacteria strains [25,26,27]. These screening gave higher “positive” rates of 92% (22 out of 24) [25], 76% (19 out of 25) [26], and 63% (38 out of 60) [27], when compared to the lower “positive” rate of 12% (21 out of 180) in this screening. The degenerate primers (A3F and A7R) are specific for the screening of NRPS genes from Actinobacteria because they are designed from the conserved sequences of A domains in the biosynthesis of six nonribosomal peptides produced by Actinobacteria strains. We have amplified all the 16S rRNAs sequences of the 21 “positive” strains. The sequencing and BLAST results showed that the 21 “positive” strains consisted of 14 Firmicutes (14 from *Bacillus*) strains and seven Actinobacteria (four from *Rhodococcus*, one from *Streptomyces*, one from *Nocardiopsis*, and one from *Mycobacterium*) stains (Table S1). These results suggest that the majority of the 180 marine bacteria in this screening are not Actinobacteria, which may be the reason for the lower “positive” rate. Other PCR screening for NRPS genes from marine bacteria using different degenerate primers gave different phyla of “positive” strains with different “positive” rates (30% and 14%) [14,28], exemplifying the degenerate primers as another important factor affecting the screening result.

We then fermented the 21 strains in small-scale (50 mL) using four different media (see Materials and Methods), and analyzed the crude extracts by HPLC. The HPLC analysis showed that strains PKU-MA00092 and PKU-MA00093 provided abundant natural products in medium M2, under the UV detection of 210 nm. Thus, strains PKU-MA00092 and PKU-MA00093 were chosen for the large-scale fermentation in medium M2 and natural products isolation. Four bacillibactin analogues (**1**–**4**), including two new compounds (**1** and **2**) were isolated from a 4.5 L fermentation of PKU-MA00093, and six bacillomycin D analogues (**5**–**10**) were isolated from a 10.0 L fermentation of PKU-MA00092 (Figure 1). The structures of compounds **1**–**10** were elucidated based on comprehensive spectroscopic analyses. Bacillibactin and bacillomycin D have been previously isolated from different *Bacillus* strains [9,10,29]. To identify the genuses of strains PKU-MA00092 and PKU-MA00093, the housekeeping 16S rRNA genes of the two strains were amplified by PCR and sequenced. BLAST on the NCBI website showed that the two 16S rRNAs were highly homologous to 16S rRNAs from *Bacillus* strains. The phylogenetic analysis was carried out based on the sequence alignments of the two 16S rRNAs with different *Bacillus* homologues, identifying PKU-MA00092 and PKU-MA00093 as two *Bacillus* strains (Figure S3).

### 2.2. Structural Elucidation of Compounds ***1**–**10***

**Figure 2:**
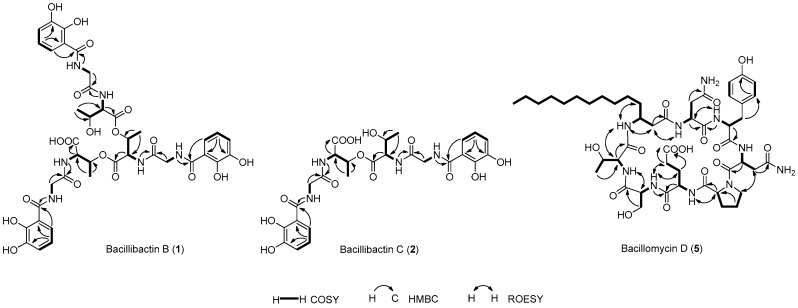
The key COSY, HMBC, and ROESY correlations of compounds **1**, **2**, and **5**.

**Figure 3:**
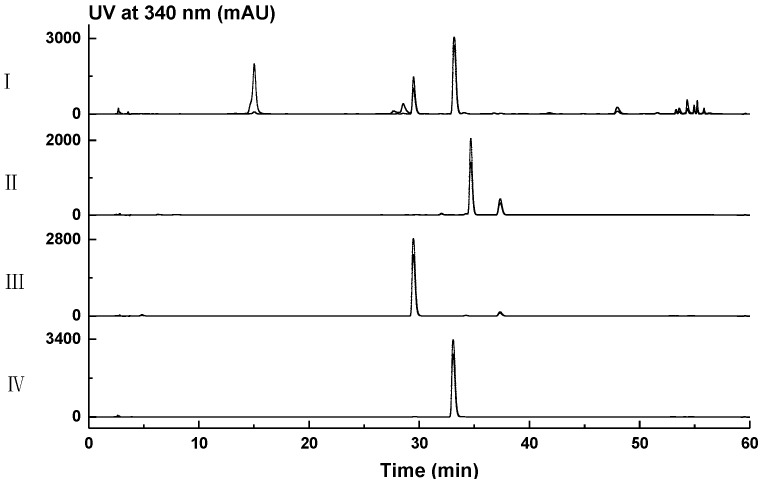
The Marfey’s analysis for the determination of absolute configuration of threonine moiety in compound **1**. (I) The HPLC analysis for the FDAA derivative of hydrolysates of compound **1**, showing the peaks for FDAA derivatives of threonine and glycine between 28 and 35 min. (II) The HPLC analysis of FDAA derivative of d-threonine. (III) The HPLC analysis of FDAA derivative of l-threonine. (IV) The HPLC analysis of FDAA derivative of glycine.

Compound **1** was obtained as a light green gum. HRESIMS analysis afforded an [M − H]^−^ ion at *m*/*z* 899.2584, giving the molecular formula of **1** as C_39_H_44_N_6_O_19_. The ^1^H NMR spectrum of **1** resembled that of bacillibactin (**3**) [30,31], except that the resonances at *δ*_H_ 5.31 (d, *J* = 6.8 Hz, H_3_-3/3′/3′′) in **3** split to *δ*_H_ 5.33 (br s, H-3), *δ*_H_ 5.38 (br s, H-3′) and *δ*_H_ 4.19 (br s, H-3′′) in **1**, and the resonances at *δ*_H_ 4.59 (br s, H_3_-2/2′/2′′) in **3** split to *δ*_H_ 4.56 (br d, *J* = 8.0 Hz, H-2), *δ*_H_ 4.79 (br d, *J* = 8.0 Hz, H-2′), and *δ*_H_ 4.38 (br d, *J* = 8.0 Hz, H-2′′) in **1** (Figure S4, Table 1). The ^13^C NMR spectrum of **1** resembled that of bacillibactin (**3**) [30,31] except that the resonances at *δ*_C_ 70.8 (C-3/3′/3′′) in **3** split to *δ*_C_ 70.7 (C-3/3′) and *δ*_C_ 66.3 (C-3′′) in **1**, the resonances at *δ*_C_ 56.6 (C-2/2′/2′′) in **3** split to *δ*_C_ 55.1 (C-2), *δ*_C_ 54.8 (C-2′) and *δ*_C_ 57.4 (C-2′′) in **1**, the resonances at *δ*_C_ 16.5 (C-4/4′/4′′) in **3** split to *δ*_C_ 16.4 (C-4), *δ*_C_ 16.3 (C-4′) and *δ*_C_ 20.2 (C-4′′) in **1**, and the resonance attributed to C-1 at *δ*_C_ 168.4 in **3** shifted downfield to *δ*_C_ 170.9 in **1** (Figure S6, Table 1). These differences in combination with the molecular formula of **1** suggested that **1** was the hydrolyzed product of **3**, which was confirmed by the MS/MS analysis based on the observation of ions [a]^−^, [b]^−^, [c]^−^, and [d]^−^ at *m*/*z* 763.2365, 706.2209, 605.1732, and 311.0882, respectively (Figure S11). The linkage relations of 2,3-dihydroxybenzoic acid (DHBA), glycine and threonine in three units were confirmed by COSY, HSQC, and HMBC experiments (Figure 2, Figures S5, S7 and S8). To further establish the absolute configuration of **1**, the Marfey’s analysis [32] was carried out. HPLC analysis of the FDAA derivatives of the amino acids in **1** unambiguously established that **1** only contained l-threonine (Figure 3). Thus, compound **1** was identified as the hydrolyzed product of bacillibactin (**3**), and named as bacillibactin B.

Compound **2** was obtained as a light green gum. HRESIMS analysis afforded an [M + Na]^+^ ion at *m*/*z* 629.1702, giving the molecular formula of **2** as C_26_H_30_N_4_O_13_. The ^1^H NMR spectrum of **2** resembled that of S_VK21_ (**4**) [33] except that the resonances at *δ*_H_ 4.20 (d, *J* = 6.0 Hz, H-3) in **4** split to *δ*_H_ 5.33 (m, H-3) and *δ*_H_ 4.21 (m, H-3′) in **2**, the resonances at *δ*_H_ 4.10 (br s, H-2) in **4** split to *δ*_H_ 4.61 (dd, *J* = 8.8, 2.5 Hz, H-2) and *δ*_H_ 4.39 (dd, *J* = 8.5, 2.5 Hz, H-2′) in **2**, the resonances at *δ*_H_ 1.04 (d, *J* = 6.3 Hz, H-4) in **4** split to *δ*_H_ 1.15 (d, *J* = 6.4 Hz, H-4) and *δ*_H_ 1.05 (d, *J* = 6.2 Hz, H-4′) in **2**, and the resonances at *δ*_H_ 7.86 (d, *J* = 8.3 Hz, 2-NH) in **4** split to *δ*_H_ 8.36 (d, *J* = 8.8 Hz, 2-NH) and *δ*_H_ 8.06 (d, *J* = 8.5 Hz, 2′-NH) in **2** (Figure S12, Table 2). The ^13^C NMR spectrum of **2** resembled that of S_VK21_ (**4**) [33] except that the resonances at *δ*_C_ 66.2 (C-3) in **4** split to *δ*_C_ 70.8 (C-3) and *δ*_C_ 66.4 (C-3′) in **2**, the resonances at *δ*_C_ 57.5 (C-2) in **4** split to *δ*_C_ 55.0 (C-2) and *δ*_C_ 57.4 (C-2′) in **2**, and the resonances at *δ*_C_ 20.1 (C-4) in **4** split to *δ*_C_ 16.5 (C-4) and *δ*_C_ 20.1 (C-4′) in **2** (Figure S14, Table 2). These differences in combination with the molecular formula of **2** suggested that **2** was the esterification product of two S_VK21_ (**4**) units with the linkage between the two threonine residues. This was confirmed by the MS/MS analysis based on the observation of ions [a + H + Na]^+^, [b + H + Na]^+^, and [c + H + Na]^+^ at *m*/*z* 493.1541, 436.1345, and 335.0855, respectively (Figure S19). The linkage relations of 2,3-dihydroxybenzoic acid (DHBA), glycine, and threonine in the two units were confirmed by COSY, HSQC, and HMBC experiments (Figure 2, Figures S13, S15 and S16). The two l-threonine residues in **2** were established based on their similar chemical shifts, coupling constants and same biosynthetic pathway with that of **4**. Thus, compound **2** was identified as the esterification product of two S_VK21_ (**4**) units and named as bacillibactin C.

Compounds **3** and **4** was identified as bacillibactin and S_VK21_, respectively, by comparison of their spectroscopic data with that of the authentic compounds [30,31,33]. The biological role of bacillibactin as the catecholic siderophore in iron acquisition has been characterized in details [10,34]. The biosynthetic research of bacillibactin has revealed how nature utilized NRPS machinery to synthesized this molecule. The standalone adenylation protein in the starter module, DhbE, activates 2,3-dihydroxybenzic acid (DHBA), and loaded this starter unit onto the first peptidyl carrier protein (PCP), initiating the downstream peptide elongation; the crystal structure of DhbE revealed the ten amino acids in the active sites determining the substrate selectivity [35]; the three-molular NRPS catalyzes the condensation between DHBA, glycine, and l-threonine to generate PCP-tethered linear tripeptides intermediate (thioester of compound **4**), then the thioesterase domain (TE) in the last module catalyzes the polymerization of S_VK21_ units to generate the TE-tethered ester of compound **2**, followed by the formation of TE-tethered ester of compound **1** and the intramolecular cyclization to generate bacillibactin (**3**) [35,36]. In this study, we isolated compounds **1**, **2**, and **4** whose structures are corresponding to the three biosynthetic intermediates in bacillibactin biosynthesis. The HPLC analysis of the crude extract of PKU-MA00093 using an increasing gradient of MeOH in H_2_O (containing no acids or bases) as the moble phase showed the co-occurrence of the peaks for compounds **1**–**4** (data not shown). Therefore, compounds **1**, **2**, and **4** may be isolated as the shunt products released from the NRPS machinery in the biosynthesis of bacillibactin (**3**).

Compound **5** was obtained as a white amorphous powder. HRESIMS analysis afforded an [M − H]^−^ ion at *m*/*z* 1029.5260, giving the molecular formula of **5** as C_48_H_74_N_10_O_15_. The ^1^H NMR and ^13^C NMR spectra (Figures S27 and S29) resembled that of bacillomycin D [29,37]. The COSY, HSQC, and HMBC experiments (Figure 2, Figures S28, S30 and S31) confirmed the presence of two asparagines, one tyrosine, one proline, one glutamate, one serine, one threonine, and one C_14_-fatty acid moieties in **5**. The correlations of H-36 with 1-NH, H-1 with 5-NH, H-15 with H-21, H-18 with 23-NH, H-24 with 28-NH, H-25 with 28-NH, H-28 with 31-NH, H-31 with 37-NH, 37-NH with H-36, 37-NH with H-38 in the ROESY spectrum (Figure 2 and Figure S32) established the cyclo(Asn-Tyr-Asn-Pro-Glu-Ser-Thr-C_14_-fatty acid) structure of **5**. To confirm that the amino acids in **5** have the same absolute configurations as that in bacillomycin D, the Marfey’s analysis was carried out. HPLC analysis of the FDAA derivatives of the amino acids established that **5** contained same l-asparagine, d-asparagine, d-tyrosine, l-proline, l-glutamate, d-serine, and l-threonine residues as bacillomycin D (Figure S49). Thus, compound **5** was identified as bacillomycin D.

The analyses of the ^1^H NMR and ^13^C NMR spectra of compounds **6**–**10** showed that they contained the same cyclopeptide skeleton as compound **5**. However, their different molecular weights (see Section 3.6 part in Materials and Methods) determined by ESIMS analyses showed that they contained different fatty acid moieties. Compound **6** contained one C_15_-fatty acid moiety with a branch terminal, which was established by the HRESIMS analysis (Figure S36) and the resonances attributed to two methyl groups at *δ*_H_ 0.84 (d, *J* = 6.3 Hz, H_3_-48, and H_3_-49) in the ^1^H NMR spectrum (Figure S34, Table S4); Compound **7** contained one C_15_-fatty acid moiety with a linear terminal, which was established by the HRESIMS analysis (Figure S39) and the resonance attributed to the methyl group at *δ*_H_ 0.88 (t, *J* = 7.4 Hz, H_3_-49) in the ^1^H NMR spectrum (Figure S37, Table S5); Compound **8** contained one C_16_-fatty acid moiety with a branch terminal, which was established by the ESIMS analysis (Figure S42) and the resonances attributed to two methyl groups at *δ*_H_ 0.84 (d, *J* = 6.5 Hz, H_3_-49 and H_3_-50) in the ^1^H NMR spectrum (Figure S40, Table S6); Compound **9** contained one C_16_-fatty acid moiety with a linear terminal, which was established by the ESIMS analysis (Figure S45) and the resonance attributed to the methyl group at *δ*_H_ 0.87 (t, *J* = 6.4 Hz, H_3_-50) in the ^1^H NMR spectrum (Figure S43, Table S7); Compound **10** contained one C_17_-fatty acid moiety with a branch, which was established by the ESIMS analysis (Figure S48) and the resonances attributed to two methyl groups at *δ*_H_ 0.84 (t, *J* = 7.3 Hz, H_3_-50) and *δ*_H_ 0.83 (d, *J* = 6.3 Hz, H_3_-51) in the ^1^H NMR spectrum (Figure S46, Table S8), and the correlations of H_3_-51 with H-48, H-48 with H_2_-49, H_2_-49 with H_3_-50 in the COSY spectrum (Figure S47). Thus, compounds **6**, **8**, and **10** were identified as iso-C_15_ bacillomycin D [38], iso-C_16_ bacillomycin D [38], and anteiso-C_17_ bacillomycin D [38], respectively. Compounds **7** and **9** were identified as C_15_ bacillomycin D and C_16_ bacillomycin D, respectively. Compounds **7** and **9** were reported previously without NMR data provided [39,40], and we here report the structures of **7** and **9** with fully assigned ^1^H NMR and ^13^C NMR data for the first time.

The separation of compounds **5**–**10** is challenging because their structures are highly similar with only the fatty acid moieties different. Due to the difficulties in the separation, compounds **5** and **6** were purified as a mixture in the cytotoxicity assay against Ehrlich carcinoma tumor cell line and hemolytic activity assay [37]. The structural elucidation of compounds **5**–**10** is also challenging, because the large molecular weights and the complex structures of **5**–**10** require large amount of samples for 1D and 2D NMR analyses. Due to the little amount obtained, two bacillomycin D analogues with C_14_ and C_15_ fatty acid moieties [41] and five bacillomycin D analogues with C_14_ to C_18_ fatty acid moieties [42] were proposed only based on LC-MS analyses. The biosynthetic gene cluster of bacillomycin D has been cloned and sequenced [12,43]. The acyl-CoA ligase domain in the first module is responsible for the ligation of the fatty acid moiety to the polyketide synthase (PKS)-NRPS hybrid assembly line. The isolation of compounds **5**–**10** in this study suggested that the acyl-CoA ligase domain possessed substrate promiscuity, which allowed for the ligation of different length and branches of fatty acid moieties to the polyketide-nonribosomal peptide hybrid macrocycle. The substrate promiscuity of the acyl-CoA ligase domain can be employed in combinatorial biosynthesis for the generation of more bacillomycin D analogues.

### 2.3. Cytotoxicity Assays against Human Cancer Cell Lines

Compounds **1**–**10** were tested for their antitumor activities. Two human cancer cell lines, liver cancer cell line HepG2, and breast cancer cell line MCF7, were used in the assays. Compounds **7**–**10** showed moderate cytotoxicities with IC_50_ values from 2.9 ± 0.1 to 8.2 ± 0.2 µM and compounds **1**–**6** show no cytotoxicities (inhibition rates were below 20% at the concentration of 10 µM) (Table 3). The isolation of **5**–**10** allowed us to analyze the effections of different fatty acid moieties on the cytotoxicities. Compound **5** with a C_14_-fatty acid moiety showed no cytotoxicities (inhibition rates were below 20% at the concentration of 10 µM); both compounds **6** and **7** contain a C_15_-fatty acid moiety, however, compound **7** with a linear C_15_-fatty acid moiety showed moderate cytotoxicities and **6** with a branched C_15_-fatty acid moiety lost the cytotoxicities, suggesting that a linear C_15_-fatty acid moiety is important to the cytotoxicities; compounds **8** and **9** containing a C_16_-fatty acid moiety showed most potent cytotoxicities; compound **10** containing one more methyl group attached at C-49 when compared to **8** or attached at C-48 when compared to **9**, showed decreased cytotoxicity against MCF7 and lost the cytotoxicity against HepG2, suggesting a C_17_-fatty acid moiety with terminal branch is disadvantageous to the cytotoxicities. In conclusion, bacillomycin D analogues with a C_16_-fatty acid moiety may possess best cytotoxicities against HepG2 and MCF7 cancer cell lines. The bacillomycin D analogues have been mostly assayed for their antifungal activities [44], especially against fungi of plant pathogens [38,40,45]. For their antitumor activities, a mixture of compounds **5** and **6**’s moderate cytotoxicities have been reported against mice Ehrlich’s carcinoma cells [37]. In another report, one lipopeptide was proposed as bacillomycin D (**5**) only based on LC-MS analysis and showed inhibitory activities against three human cancer cell lines A549, A498 and HCT-15 [46]. The cytotoxicities against human cancer cell lines HepG2 and MCF7 and the structure-activity relationships that we first revealed here enriched our understanding of the diversity of bacillomycin D analogues’ bioactivities.

## 3. Materials and Methods

### 3.1. General Experimental Procedures

Optical rotations were measured on an Autopol III automatic polarimeter (Rudolph Research Analytical, Hackettstown, NJ, USA). UV spectra were collected on a Cary 300 spectrometer (VARIAN, Palo Alto, CA, USA). IR spectra were collected on a Nicolet Nexus 470 FT-IR spectrometer (Thermo Scientific, Waltham, MA, USA). ^1^H and ^13^C NMR spectra were collected on a Bruker Avance-400FT NMR spectrometer (Bruker Corporation, Billerica, MA, USA). HRESIMS and MS/MS spectra were collected on a Waters Xevo G2 Q-TOF spectrometer (Waters, Milford, MA, USA). ESIMS spectra were collected on a Waters SQD Single Quadruple Mass spectrometer (Waters, Milford, MA, USA). HPLC analysis was performed on an Agilent 1260 series (Agilent Technologies, Santa Clara, CA, USA) with a diode array detector (Agilent Technologies, Santa Clara, CA, USA) and a C_18_ RP-column (Eclipse XDBC_18_, 150 × 4.6 mm, 5 μm, Agilent Technologies, Santa Clara, CA, USA). Semi-preparative HPLC was performed on a SSI 23201 system (Scientific Systems Inc., State College, PA, USA) with a YMC-Pack ODS-1 column (250 × 10 mm, 5 μm, YMC Co., Ltd., Shimogyo-ku, Kyoto, Japan). MPLC was performed on a LC3000 series (Beijing Tong Heng Innovation Technology, Beijing, China) with a Claricep^TM^ Flash i-series C_18_ cartridge (20–35 μm, 40 g, Bonna-Agela, Wilmington, DE, USA). Size exclusion chromatography was carried out using a Sephadex LH-20 (Pharmacia Fine Chemicals, Piscataway, NJ, USA) column.

### 3.2. Marine Bacterial Strains Isolation

Total 15 Sponge samples were collected from Yongxin Island in the South China Sea and were deposited at the State Key Laboratory of Natural and Biomimetic Drugs, Peking University, China. The isolation of the marine bacterial strains was carried out according to the published procedures [47]. The sponge samples were thoroughly washed with sterilized water containing 3% sea salt for three times, and cut into pieces of about 1 cm^3^, then homogenized in a sterilized mortar with 9 mL sterilized water containing 3% sea salt. The homogenates were incubated in a water bath at 55 °C for 6 min, and put into biosafety cabinet for 30 min, and the supernatants were serially diluted by sterilized water containing 3% sea salt and plated in triplicate on agar plates for each sponge sample. Four media, M1 (yeast extract 1 g, peptone 5 g, beef extract 1 g, FePO_4_ 0.01 g, agar 18 g, and sea salt 33 g in 1.0 L distilled water, pH 7.4), M2 (glycerol 6 mL, arginine 1 g, K_2_HPO_4_ 1 g, MgSO_4_ 0.5 g, agar 18 g, sea salt 33 g in 1.0 L distilled water, pH 7.2), M3 (soluble starch 20 g, KNO_3_ 1 g, K_2_HPO_4_ 0.5 g, MgSO_4_·7H_2_O 0.5 g, NaCl 0.5 g, FeSO_4_·7H_2_O 0.01 g, agar 18 g, sea salt 33 g in 1.0 L distilled water, pH 7.2), and M4 (sodium acetate trihydrate 5 g, peptone 0.5 g, yeast extract 0.5 g, glucose 0.5 g, sucrose 0.5 g, sodium citrate 0.05 g, malic acid 0.05 g, NH_4_NO_3_ 1 g, NH_4_Cl 0.2 g, KH_2_PO_4_ 0.5 g, agar 18 g, sea salt 33 g in 1.0 L distilled water, pH 7.6), were used for bacterial strains isolation. Each plate was supplemented with cycloheximide (50 μg/mL) and nalidixic acid (50 μg/mL) to suppress the growth of fungi and Gram-negative bacteria, and incubated at 28 °C for four weeks. Different colonies of strains were carefully selected and restreaked on agar plates with medium M1 for further proliferation and dereplication. Totally 180 bacterial strains were isolated and stored at −80 °C in 20% glycerol.

### 3.3. PCR Screening for Potential Producers of Nonribosomal Peptides

The genomic DNAs of all 180 strains were extracted following standard protocols [48]. The forward primer (5′-GCSTACSYSATSTACACSTCSGG-3′) and the reverse primer (5′-SASGTCVCCSGTSCGGTAS-3′), designed from the conserved sequences of adenylation domains (A domains) in the biosynthesis of cephamycin, vancomycin, balhimycin, actinomycin, pristinamycin, and chloroeremomycin, were used in the screening [24]. A 20 μL PCR system consisting of 10 µL Easy Taq Polymerase (Beijing TransGen Biotech, Beijing, China), 2 µL of forward and reverse primer mixture (each for 10 µM), 1 µL genomic DNA, and 7 µL sterilized water, were used. The PCR program was performed with an initial denaturation at 95 °C for 5 min, followed by 30 cycles of denaturation at 95 °C for 30 s, annealing at 57 °C for 1 min and extension at 72 °C for 1 min, followed by incubation at 72 °C for 10 min. The PCR products were analyzed by agarose gel electrophoresis (Figure S2), recovered with a gel purification kit (Beijing TransGen Biotech, Beijing, China) and sequenced to afford 21 “positive” strains.

### 3.4. Small-Scale Fermentation, HPLC

For each of the 21 “positive” strains, 50 µL of spore suspension was inoculated into 50 mL of seed medium (medium M1 broth), and incubated with a HYG-C shaker (Suzhou Peiying Laboratory Equipment, Suzhou, China) at 28 °C, 200 rpm for three days. Three milliliter of the resultant seed culture was inoculated into 50 mL of production media, and the fermentation continued at 28 °C, 200 rpm for seven days. Four different production media, media M1, M2, and M3 broth, and M5 (soluble starch 10 g, casein 0.3 g, K_2_HPO_4_ 2 g, KNO_3_ 2 g, MgSO_4_·7H_2_O 0.05 g, NaCl 2 g, FeSO_4_·7H_2_O 0.01 g, CaCO_3_ 0.02 g, sea salt 33 g in 1.0 L distilled water, pH 7.2), were used in the small-scale fermentation. The Diaion HP20 (2 g/100 mL, Mitsubishi Chemical Corporation, Tokyo, Japan) and Amberlite XAD-16 (2 g/100 mL, Sigma-Aldrich, St. Louis, MO, USA) resins were added 10 h before the fermentation finished. The resins and cell mass were harvested by centrifugation and extracted with MeOH. The MeOH extracts were concentrated and analyzed by HPLC. The HPLC analysis was carried out with a flow rate of 1 mL/min with UV detection at 210 nm, using a gradient elution program from 5% MeOH in H_2_O to 100% MeOH over 45 min.

### 3.5. Phylogenetic Analysis

The phylogenetic analysis of strains PKU-MA00092 and PKU-MA00093 was carried out by sequence alignments of their 16S rRNAs with different *Bacillus* homologues. The primers 27F (5′-AGAGTTTGATCMTGGCTCAG-3′) and 1492R (5′-TACGGYTACCTTGTTACGACTT-3′) [49] were used to amplify the 16S rDNA genes (GeneBank accession number MF774821 for PKU-MA00092 and KY780192 for PKU-MA00093). Homologous genes were searched using BLAST on the NCBI website and the phylogenetic tree was generated with Mega 5.1 using the Neighbor-Joining algorithm.

### 3.6. Large-Scale Fermentation and Isolation

The large-scale fermentation of *Bacillus* sp. PKU-MA00093 and *Bacillus* sp. PKU-MA00092 in medium M2 broth were carried out with similar procedures used in the small-scale fermentation. For *Bacillus* sp. PKU-MA00092 or PKU-MA00093, 50 µL of spore suspension was inoculated into 50 mL of seed medium (medium M1 broth, pH 7.4) and incubated with HYG-C shakers at 28 °C, 200 rpm for three days. Twelve milliliter of the resultant seed culture was inoculated into 200 mL of medium M2 broth (pH 7.2) in 1 L Erlenmeyer flasks, and the fermentation continued at 28 °C, 200 rpm for seven days. The Diaion HP20 (2 g/100 mL), and Amberlite XAD-16 (2 g/100 mL) resins were added 10 h before the fermentation finished. The resins and cell mass were harvested by centrifugation and extracted with MeOH.

A 4.5 L fermentation of *Bacillus* sp. PKU-MA00093 gave a 4.3 g of MeOH extract. The MeOH extract was concentrated in a vacuum and suspended in deionized water, extracted with ethyl acetate for three times. The ethyl acetate extracts were combined (1.4 g) and loaded onto MPLC using step-gradient elution of MeOH in H_2_O (10%, 20%, 35%, 50%, 70%) to yield five fractions (F1–F5). Fraction F4 (147 mg) was purified by semi-preparative HPLC with MeOH/H_2_O (44/56, *v*/*v*) as the mobile phase to yield **1** (4.6 mg); fraction F2 (169 mg) was purified by semi-preparative HPLC with MeOH/H_2_O (37/63, *v*/*v*) as the mobile phase to yield compound **2** (7.8 mg) and **4** (1.4 mg); fraction F3 (231 mg) was purified by semi-preparative HPLC with MeOH/H_2_O (40/60, *v*/*v*) as the mobile phase to yield **3** (17.4 mg). All of the semi-preparative HPLCs were carried out at a flowrate of 2 mL/min and under the detection of 248 nm.

A 10.0 L fermentation of *Bacillus* sp. PKU-MA00092 gave a 1.5 g of MeOH extract. The MeOH extract was concentrated and loaded onto MPLC using step-gradient elution of MeOH in H_2_O (20%, 40%, 60%, 80%) to yield four fractions (F1–F4). Fraction F4 (368 mg) was purified repeatedly by semi-preparative HPLC with CH_3_CN/H_2_O (45/55 *v*/*v*) containing 0.01% TFA as the mobile phase, to afford compounds **5** (36.5 mg), **6** (15.7 mg), **7** (11.4 mg), **8** (13.3 mg), **9** (14.5 mg) and **10** (3.1 mg), at a flowrate of 2 mL/min and under the detection of 210 nm.

Bacillibactin B (**1**): light green gum; ${\left[\alpha \right]}_{D}^{25}$ +37 (*c* 0.23, MeOH); UV (MeOH) λ_max_ (log *ε*): 207 (1.47), 248 (0.40), 312 (0.16) nm; IR (KBr) ν_max_ 3317, 1740, 1642, 1541, 1266, 1023, 745 cm^−1^ (Figure S10); ^1^H NMR and ^13^C NMR data, Table 1; HRESIMS *m*/*z* 899.2584 [M − H]^−^ (calcd. for C_39_H_43_N_6_O_19_, 899.2580).

Bacillibactin C (**2**): light green gum; ${\left[\alpha \right]}_{D}^{25}$ +40 (*c* 0.27, MeOH); UV (MeOH) λ_max_ (log *ε*): 206 (2.22), 249 (1.12), 312 (0.54) nm; IR (KBr) ν_max_ 3320, 1738, 1644, 1540, 1264, 1022, 746 cm^−1^ (Figure S18); ^1^H NMR and ^13^C NMR data, Table 2; HRESIMS *m*/*z* 605.1740 [M − H]^−^ (calcd. for C_26_H_29_N_4_O_13_, 605.1731); *m*/*z* 629.1702 [M + Na]^+^ (calcd. for C_26_H_30_N_4_O_13_Na, 629.1705).

Bacillibactin (**3**): light green gum; ^1^H NMR and ^13^C NMR data, Supplementary Table S2; HRESIMS *m*/*z* 883.2621 [M + H]^+^ (calcd. for C_39_H_43_N_6_O_18_, 883.2631).

S_VK21_ (**4**): light green gum; ^1^H NMR and ^13^C NMR data, Supplementary Table S2; HRESIMS *m*/*z* 311.0878 [M − H]^−^ (calcd. for C_13_H_15_N_2_O_7_, 311.0878).

Bacillomycin D (**5**): white amorphous powder; ^1^H NMR and ^13^C NMR data, Supplementary Table S3; HRESIMS *m*/*z* 1029.5260 [M − H]^−^ (calcd. for C_48_H_73_N_10_O_15_, 1029.5254).

iso-C_15_ Bacillomycin (**6**): white amorphous powder; ^1^H NMR and ^13^C NMR data, Supplementary Table S4; HRESIMS *m*/*z* 1043.5421 [M − H]^−^ (calcd. for C_49_H_75_N_10_O_15_, 1043.5411).

C_15_ Bacillomycin (**7**): white amorphous powder; ^1^H NMR and ^13^C NMR data, Supplementary Table S5; HRESIMS *m*/*z* 1043.5411 [M − H]^−^ (calcd. for C_49_H_75_N_10_O_15_, 1043.5411).

iso-C_16_ Bacillomycin (**8**): white amorphous powder; ^1^H NMR and ^13^C NMR data, Supplementary Table S6; ESIMS *m*/*z* 1057.34 [M − H]^−^.

C_16_ Bacillomycin (**9**): white amorphous powder; ^1^H NMR and ^13^C NMR data, Supplementary Table S7; ESIMS *m*/*z* 1057.43 [M − H]^−^.

anteiso-C_17_ Bacillomycin (**10**): white amorphous powder; ^1^H NMR data, Supplementary Table S8; ESIMS *m*/*z* 1071.45 [M − H]^−^.

### 3.7. Marfey’s Analysis for Compounds ***2*** and ***5***

The Marfey’s analysis of compounds **2** and **5** was carried out with same procedure [32,50]. The peptide (1.0 mg) was hydrolyzed with 500 μL of 6 N HCl in a sealed glass vial (1.5 mL) at 110 °C for 18 h, and dried with N_2_ gas. The hydrolysate was dissolved in 50 μL of H_2_O, followed by the addition of 100 μL FDAA (1-fluoro-2,4-dinitrophenyl-5-l-alanine amide, 1% in acetone, 3.7 µmol) and 20 μL of 1 M NaHCO_3_. The mixture was heated at 45 °C for 1 h, followed by the addition of 10 μL of 2 M HCl to terminate the reaction. The FDAA-derivatives were dried with N_2_ gas and dissolved in 1 mL of 50% CH_3_CN in H_2_O for HPLC analysis. The HPLC was carried out with a C_18_ column (50 × 4.6 mm, 5 µm, Grace Corporate, Columbia, MD, USA) and with a program of linear gradient of 10% CH_3_CN in H_2_O containing 0.1% TFA to 40% CH_3_CN in H_2_O containing 0.1% TFA over 40 min, at a flow rate of 1.0 mL/min, and under the detection of 340 nm. The FDAA-derivatives of standard l or d-amino acids were used as comparison.

### 3.8. Cytotoxicity Assays for Compounds ***1**–**10***

HepG2 and MCF7 cell lines were purchased from Peking Union Medical College, Cell Bank (Beijing, China). In this study, the cells were grown in Dulbecco’s modified Eagle’s medium (Hyclone, Waltham, MA, USA), supplemented with 10% fetal bovine serum (FBS, PAN Biotech, Aidenbach, Germany), penicillin (100 U/mL), and streptomycin (100 µg/mL) in a humidified incubator containing 95% air and 5% CO_2_ at 37 °C. Cell viability was detected by MTT colorimetric assay. Briefly, the cells were treated with different concentrations of compounds (in DMSO stock) for 48 h, with taxol as the positive control. Then, culture supernatants were replaced with medium containing 0.5 mg/mL MTT for 4 h. The supernatant was removed and 100 μL of dimethyl sulfoxide was added. Absorbance was detected at 550 nm and cell viability was expressed as the mean percentage of the absorbance in treated vs. control cells. Different concentrations of compounds ranging from 0.1 to 40 µM were tested and each of the concentration was tested in parallel triplicate. Six concentration points in the slope region were used in the calculation of IC_50_ values. The IC_50_ values were calculated from the concentration–response curves that were generated by nonlinear regression with Graph Pad Prism 5 software (Graph Pad Software, Inc., San Diego, CA, USA).

## Figures and Tables

**Table 1:** The ^1^H (400 MH_Z_) and ^13^C NMR (100 MH_Z_) data of compound **1** in DMSO-*d*_6_.

Position	*δ*_H_ (*J* in Hz)	*δ*_C_, Type	Position	*δ*_H_ (*J* in Hz)	*δ*_C_, Type	Position	*δ*_H_ (*J* in Hz)	*δ*_C_, Type
1		170.9, C	1′		168.0, C	1′′		169.3 ^b^, C
2	4.56, br d (8.0)	55.1, CH	2′	4.79, br d (8.0)	54.8, CH	2′′	4.38, br d (7.9)	57.4, CH
3	5.33, br s	70.7, CH	3′	5.38, br s	70.7, CH	3′′	4.19, br s	66.3, CH
4	1.09, d (6.0)	16.4 ^a^, CH_3_	4′	1.16, d (6.1)	16.3 ^a^, CH_3_	4′′	1.06, d (5.9)	20.2, CH_3_
5		168.9 ^b^, C	5′		169.1 ^b^, C	5′′		169.2 ^b^, C
6	4.09, m	41.8 ^c^, CH_2_	6′	4.09, m	41.9 ^c^, CH_2_	6′′	4.03, m	42.2 ^c^, CH_2_
7		169.49 ^b^, C	7′		169.52 ^b^, C	7′′		169.7 ^b^, C
8		115.33 ^d^, C	8′		115.2 ^d^, C	8′′		115.26 ^d^, C
9		149.05 ^e^, C	9′		149.14 ^e^, C	9′′		149.2 ^e^, C
10		146.1, C	10′		146.1, C	10′′		146.1, C
11	6.93, m	118.8, CH	11′	6.93, m	118.8, CH	11′′	6.93, m	118.8, CH
12	6.69, m	118.1, CH	12′	6.69, m	118.1, CH	12′′	6.69, m	118.1, CH
13	7.31, m	117.7 ^f^, CH	13′	7.31, m	117.6 ^f^, CH	13′′	7.31, m	117.6 ^f^, CH
2-NH	8.33, br s		2′-NH	8.47, br s		2′′-NH	8.14, br s	
6-NH	9.04, d (5.1)		6′-NH	9.04, d (5.1)		6′′-NH	9.13, m	
9-OH	12.31, br s		9′-OH	12.31, br s		9′′-OH	12.31, br s	
10-OH	9.23, br s		10′-OH	9.23, br s		10′′-OH	9.23, br s	

^a–f^ Assignments may be interchanged.

**Table 2:** The ^1^H (400 MH_Z_) and ^13^C NMR (100 MH_Z_) data of compound **2** in DMSO-*d*_6_.

Position	*δ*_H_ (*J* in Hz)	*δ*_C_, Type	Position	*δ*_H_ (*J* in Hz)	*δ*_C_, Type
1		170.7, C	1′		169.4 ^a^, C
2	4.61, dd (2.5, 8.8)	55.0, CH	2′	4.39, dd (2.5, 8.5)	57.4, CH
3	5.33, m	70.8, CH	3′	4.21, m	66.4, CH
4	1.15, d (6.4)	16.5, CH_3_	4′	1.05, d (6.2)	20.1, CH_3_
5		168.9 ^a^, C	5′		169.0 ^a^, C
6	4.06, m	41.9 ^b^, CH_2_	6′	4.06, m	42.1 ^b^, CH_2_
7		169.5 ^a^, C	7′		169.6 ^a^, C
8		115.3, C	8′		115.3, C
9		149.2, C	9′		149.2, C
10		146.1, C	10′		146.1, C
11	6.93, d (7.6)	118.8, CH	11′	6.93, d (7.6)	118.8, CH
12	6.70, t (7.6)	118.1, CH	12′	6.70, t (7.6)	118.1, CH
13	7.32, d (7.6)	117.7, CH	13′	7.32, d (7.6)	117.7, CH
2-NH	8.36, d (8.8)		2′-NH	8.06, d (8.5)	
6-NH	9.06, br s		6′-NH	9.06, br s	
9-OH	12.36, br s		9′-OH	12.36, br s	
10-OH	9.27, br s		10′-OH	9.27, br s	

^a,b^ Assignments may be interchanged.

**Table 3:** The cytotoxicity assays of compounds **7**–**10**.

	Cytotoxicity (IC_50_ Values in µM)				
Cell Lines	7	8	9	10	Taxol
HepG2	8.2 ± 0.2	5.1 ± 0.2	4.9 ± 0.2	>10	0.075 ± 0.004
MCF7	4.2 ± 0.1	2.9 ± 0.1	3.3 ± 0.1	7.2 ± 0.2	0.043 ± 0.003

## Supplementary Materials

The following are available online at www.mdpi.com/1660-3397/16/1/22/s1, Figure S1: nonribosomal peptides from marine-derived *Bacillus* species, Figure S2: the agarose gel electrophoresis analysis of the 21 “positive” PCR products, Figure S3: the phylogenetic analysis of strains PKU-MA00092 and PKU-MA00093, Figures S4–S11: the NMR, MS and IR spectra of compound **1**, Figures S12–S19: the NMR, MS and IR spectra of compound **2**, Figures S20–S23: the NMR and MS spectra of compound **3**, Figures S24–S26: the NMR and MS spectra of compound **4**, Figures S27–S33: the NMR and MS spectra of compound **5**, Figures S34–S36: the NMR and MS spectra of compound **6**, Figures S37–S39: the NMR and MS spectra of compound **7**, Figures S40–S42: the NMR and MS spectra of compound **8**, Figures S43–S45: the NMR and MS spectra of compound **9**, Figures S46–S48: the NMR and MS spectra of compound **10**, Figure S49: the Marfey’s analysis of compound **5**. Table S1: the homologues of the 21 “positive” strains and their PCR products, Tables S2–S8: the ^1^H NMR and ^13^C NMR data of compounds **3**–**10**.
