# Supplementary material for: Bacillibactin and Bacillomycin Analogues with Cytotoxicities against Human Cancer Cell Lines from Marine Bacillus sp. PKU-MA00093 and PKU-MA00092
Source: Mar Drugs. 2018 Jan 10;16(1):22. doi: 10.3390/md16010022 (PMC5793070; doi:10.3390/md16010022)

Bacillibactin and Bacillomycin Analogues with Cytotoxicities against Human Cancer Cell Lines from Marine *Bacillus* sp. PKU-MA00093 and PKU-MA00092

Mengjie Zhou, Fawang Liu, Xiaoyan Yang, Jing Jin, Xin Dong, Ke-Wu Zeng, Dong Liu, Yingtao Zhang, Ming Ma* and Donghui Yang*

State Key Laboratory of Natural and Biomimetic Drugs, Department of Natural Medicines, School of Pharmaceutical Sciences, Peking University, 38 Xueyuan Road, Haidian District, Beijing 100191, China.

Mengjie Zhou and Fawang Liu contributed equally

*Correspondence: mma@bjmu.edu.cn; ydhui@bjmu.edu.cn

Supplementary Informations (SI)

Table S1. The homologues of the 21 "positive" strains and their PCR products. S3

Table S2. The ^{1}H and ^{13}C NMR data of compounds **3** and **4** in DMSO-d_6. S4

Table S3. The ^{1}H and ^{13}C NMR data of compound **5** in pyridine-d_5. S5

Table S4. The ^{1}H and ^{13}C NMR data of compound **6** in pyridine-d_5. S6

Table S5. The ^{1}H and ^{13}C NMR data of compound **7** in pyridine-d_5. S7

Table S6. The ^{1}H and ^{13}C NMR data of compound **8** in pyridine-d_5. S8

Table S7. The ^{1}H and ^{13}C NMR data of compound **9** in pyridine-d_5**.** S9

Table S8. The ^{1}H NMR data of compound **10** in pyridine-d_5. S10

Figure S1. Nonribosomal peptides from marine-derived *Bacillus* species. S11

Figure S2. The agarose gel electrophoresis analysis of the 21 "positive" PCR products. S12

Figure S3. The phylogenetic analysis of strains PKU-MA00092 and PKU-MA00093. S13

Figure S4-S10. The ^{1}H NMR, COSY, ^{13}C NMR, HSQC, HMBC, HRESIMS and IR spectra of compound **1**. S14-20

Figure S11. The MS/MS analysis of compound **1**. S21

Figure S12-18. The ^{1}H NMR, COSY, APT, HSQC, HMBC, HRESIMS and IR spectra of compound **2**. S22-28

Figure S19. The MS/MS analysis of compound **2**. S29

Figure S20-23. The ^{1}H NMR, APT, HMBC, HRESIMS spectra of compound **3**. S30-33

Figure S24-26. The ^{1}H NMR, ^{13}C NMR, HRESIMS spectra of compound **4**. S34-36

Figure S27-33. The ^{1}H NMR, COSY, ^{13}C NMR, HSQC, HMBC, ROSEY and HRESIMS spectra of compound **5**. S37-43

Figure S34-36. The ^{1}H NMR, ^{13}C NMR, HRESIMS spectra of compound **6**. S44-46

Figure S37-39. The ^{1}H NMR, ^{13}C NMR, HRESIMS spectra of compound **7**. S47-49

Figure S40-42. The ^{1}H NMR, ^{13}C NMR, ESIMS spectra of compound **8**. S50-52

Figure S43-45. The ^{1}H NMR, ^{13}C NMR, ESIMS spectra of compound **9**. S53-55

Figure S46-48. The ^{1}H NMR, COSY, ESIMS spectra of compound **10**. S56-58

Figure S49. The Marfey's analysis of compound **5**. S59

Table S1. The homologues of the 21 "positive" strains based on 16S rRNA comparison, and the homologues of their PCR products. The right three columns show the identities of the highest homologues with the PCR products, the accession numbers of the highest homologues of the PCR products and the predicted amino acid substrates (the numbers in parentheses show the percentage identities with nearest signatures) of the A domains by using the web server NRPSpredictor2 [1], respectively.

Positive strains	Closest relatives by blastn	Homologues of PCR products	Identities	Accession numbers	Predicted amino acids
PKU-MA00072	*Bacillus licheniformis*	NRPS [*Bacillus licheniformis*]	96%	EHK82944.1	Leu (90%)
PKU-MA00082	*Rhodococcus pyridinivorans*	NRPS [*Rhodococcus* sp.]	96%	WP_052227234	Phe (60%)
PKU-MA00090	*Bacillus licheniformis*	NRPS [*Bacillus licheniformis*]	99%	WP_044789674.1	Leu (90%)
PKU-MA00091	*Bacillus paralicheniformis*	NRPS [*Bacillus licheniformis*]	96%	AAD32132.1	Asp (90%)
PKU-MA00092	*Bacillus velezensis*	NRPS [*Bacillus amyloliquefaciens*]	97%	WP_060675312.1	Glu (90%)
PKU-MA00093	*Bacillus endophyticus*	NRPS [*Bacillus licheniformis*]	99%	WP_011197536.1	Leu (90%)
PKU-MA00095	*Bacillus sonorensis*	NRPS [*Bacillus licheniformis*]	96%	WP_044789674.1	Leu (90%)
PKU-MA00096	*Bacillus sonorensis*	NRPS [*Bacillus licheniformis*]	96%	WP_044789674.1	Leu (90%)
PKU-MA00103	*Bacillus licheniformis*	NRPS [*Bacillus licheniformis*]	96%	WP_044789674.1	Leu (90%)
PKU-MA00110	*Bacillus sonorensis*	NRPS [*Bacillus paralicheniformis*]	96%	WP_059231730.1	Asp (90%)
PKU-MA00117	*Rhodococcus pyridinivorans*	NRPS [*Rhodococcus* sp.]	98%	WP_033096084.1	Thr (90%)
PKU-MA00125	*Bacillus licheniformis*	NRPS [*Bacillus paralicheniformis*]	91%	WP_059231730.1	Asp (90%)
PKU-MA00147	*Bacillus licheniformis*	NRPS [*Bacillus licheniformis*]	96%	WP_044789674.1	Leu (90%)
PKU-MA00149	*Rhodococcus pyridinivorans*	NRPS [*Rhodococcus pyridinivorans*]	96%	WP_006553896.1	Orn (90%)
PKU-MA00152	*Rhodococcus pyridinivorans*	NRPS [*Rhodococcus pyridinivorans*]	99%	WP_006554805.1	Phe (50%)
PKU-MA00156	*Bacillus oceani* strain	NRPS [*Rhodococcus* sp.]	99%	WP_037218052.1	Phe (60%)
PKU-MA00173	*Streptomyces sedi strain*	NRPS [*Micromonospora* sp.]	98%	EEP74799.1	Cys (50%)
PKU-MA00181	*Nocardiopsis dassonvillei*	NRPS [*Bacillus licheniformis*]	99%	WP_044789674.1	Leu (90%)
PKU-MA00183	*Brevibacillus parabrevis*	NRPS [*Brevibacillus* sp.]	96%	WP_007729123.1	Leu (80%)
PKU-MA00191	*Mycobacterium neoaurum*	NRPS [*Mycobacterium* sp.]	82%	WP_057167572.1	Alaninol (80%)
PKU-MA00197	*Bacillus licheniformis*	NRPS [*Rhodococcus rhodochrous*]	86%	WP_016693962.1	Gln (60%)

1. Rottig, M.; Medema M.H.; Blin, K.; Weber, T.; Rausch, C.; Kohlbacher, O. NRPSpredictor2--a web server for predicting NRPS adenylation domain specificity. *Nucleic. Acids Res*. **2011**, *39*, W362-367.

Table S2. The ^{1}H (400 MHz) and ^{13}C NMR (100 MHz) data of compounds **3** and **4** in DMSO-d_6.

	**3**			**4**	
position	δ_H, mult.(*J* in Hz)	δ_C type	position	δ_H, mult.(*J* in Hz)	δ_C type
1, 1', 1"		168.4, C	1		168.6, C
2, 2', 2"	4.59,br s	56.6, CH	2	4.10, br s	57.5, CH
3, 3', 3"	5.31, d (6.8)	70.8, CH	3	4.20, d (6.0)	66.2, CH
4, 4', 4"	1.18, d (6.1)	16.5, CH_3	4	1.04, d (6.3)	20.1, CH_3
5, 5', 5"		169.8, C	5		172.0, C
6, 6', 6"	4.28, d (13.6)	42.5, CH_2	6	4.00, d (3.7)	42.1, CH_2
	4.05, m				
7, 7', 7"		169.3, C	7		169.6, C
8, 8', 8"		115.7, C	8		115.2, C
9, 9', 9"		148.5, C	9		149.4, C
10, 10', 10"		146.1, C	10		146.2, C
11, 11', 11"	6.93, d (7.8)	118.8, CH	11	6.93, d (8.3)	118.8, CH
12, 12', 12"	6.70, t (7.8)	118.2, CH	12	6.70, t (8.3)	118.0, CH
13, 13', 13"	7.33, d (7.8)	118.0, CH	13	7.31, d (8.3)	117.6, CH
2, 2', 2"-NH	8.31, br s		2-NH	7.86, d (8.3)	
6, 6', 6"-NH	9.18, br s		6-NH	9.09, m	
9, 9', 9"-OH	11.97, br s				
10, 10', 10"-OH	9.31, br s				

Table S3. The ^{1}H (400 MHz) and ^{13}C NMR (100 MHz) data of compound **5** in pyridine-d_5.

				**5**				
position	δ_H, mult.(J in Hz)	δ_C, type	position	δ_H, mult.(J in Hz)	δ_C, type	position	δ_H, mult.(J in Hz)	δ_C, type
**L-Asn-1**			14-NH	8.90, br s		31	4.83^c, m	59.7, CH
1	5.27, m	53.1, CH	16-NH$_2$	8.51, br s; 7.88, br s		32	4.97, m	66.5, CH
2	3.04^a, m; 3.02^a, m	37.5, CH$_2$	**L-Pro**			33	1.35^d, m	21.2, CH$_3$
3		172.0, C	18	4.73, t (6.6)	62.6, CH	34		171.4, CH
4		174.0, C	19	2.12, m; 2.11, m	30.1^b, CH$_2$	31-NH	8.09, d (8.6)	
1-NH	8.90, br s		20	1.88, m; 1.69, m	25.3, CH$_2$	**D-β-AA**		
3-NH$_2$	8.32, br s; 7.82, br s		21	4.25, m; 4.06, m	48.9, CH$_2$	35		173.0, C
**D-Tyr**			22		172.7, C	36	2.63, m; 2.43, m	42.2, CH$_2$
5	5.33, m	56.2, CH	**L-Glu**			37	4.62, m	47.8, CH
6	3.71, dd (4.5, 14.0); 3.38, m	36.8, CH$_2$	23	4.86^c, m	55.6, CH	38	1.59, m; 1.51, m	36.0, CH$_2$
7		129.0, C	24	2.81, m; 2.66, m	28.2, CH$_2$	39	1.34^d, m	26.4, CH$_2$
8, 12	7.50, d (8.0)	131.7, CH	25	3.00^a, m; 2.98^a, m	32.4, CH$_2$	40	1.19-1.16^e	29.91^b, CH$_2$
9, 11	7.09, d (8.0)	116.5, CH	26		174.1, C	41	1.19-1.16^e	29.94^b, CH$_2$
10		157.8, C	27		173.6, C	42	1.19-1.16^e	30.18^b, CH$_2$
13		173.1, C	23-NH	8.26, br s		43	1.19-1.16^e	30.20^b, CH$_2$
5-NH	9.64, br s		**D-Ser**			44	1.19-1.16^e	30.22^b, CH$_2$
**D-Asn-2**			28	4.89^c, m	58.4, CH	45	1.19-1.16^e	30.24^b, CH$_2$
14	5.41, m	50.6, CH	29	4.36, m	63.9, CH$_2$	46	1.19-1.16^e	32.4, CH$_2$
15	3.59, m; 3.19, dd (4.6, 15.2)	38.2, CH$_2$	30		172.0, C	47	1.24, m	23.2, CH$_2$
16		173.0, C	28-NH	8.80, br s		48	0.85, t (6.7)	14.6, CH$_3$
17		172.8, C	**L-Thr**			37-NH	8.04, d (9.2)	

$^{a, c, d, e}$ Overlapped. ^{b}Assignments may be interchanged.

Table S4. The ^{1}H (400 MHz) and ^{13}C NMR (100 MHz) data of compound **6** in pyridine-d_5.

				**6**				
position	δ_H, mult.(J in Hz)	δ_C, type	position	δ_H, mult.(J in Hz)	δ_C, type	position	δ_H, mult.(J in Hz)	δ_C, type
**L-Asn-1**			16-NH_2	8.87[d], br s; 7.92, br s		33	1.36[i], d (5.8)	21.1, CH_3
1	5.31[a], m	53.3, CH	**L-Pro**			34		172.1[c], CH
2	3.07[b], m; 3.03[b], m	37.5, CH_2	18	4.75, t (6.7)	62.4, CH	31-NH	8.43[d], br s	
3		173.5[c], C	19	2.12, m; 2.10, m	30.2[f], CH_2	**D-β-AA**		
4		172.8[c], C	20	1.86, m; 1.64[g], m	25.3, CH_2	35		173.2[c], C
1-NH	8.97[d], br s		21	4.26, m; 4.05, m	48.9, CH_2	36	2.65, m; 2.54, m	42.5, CH_2
3-NH_2	8.58[d], br s; 7.87, br s		22		172.1[c], C	37	4.64, m	47.9, CH
**D-Tyr**			**L-Glu**			38	1.64[g], m; 1.47[j], m	36.1, CH_2
5	5.31[a], m	56.3, CH	23	4.94[h], m	55.8, CH	39	1.34[i], m	26.4, CH_2
6	3.76, m;<br>3.48, m	36.7, CH_2	24	2.80, m; 2.69, m	28.0, CH_2	40	1.19-1.16[k]	29.9[f], CH_2
7		129.2, C	25	3.15[e], m; 2.98[b], m	32.4, CH_2	41	1.19-1.16[k]	30.2[f], CH_2
8, 12	7.51, d (7.7)	131.6, CH	26		175.8, C	42	1.19-1.16[k]	30.2[f], CH_2
9, 11	7.09, d (7.7)	116.5, CH	27		171.5, C	43	1.19-1.16[k]	30.3[f], CH_2
10		157.8, C	23-NH	8.20[d], br s		44	1.19-1.16[k]	30.3[f], CH_2
13		173.9[c], C	**D-Ser**			45	1.19-1.16[k]	30.5[f], CH_2
5-NH	9.59, br s		28	4.98[h], m	58.3, CH	46	1.19-1.16[k]	39.6, CH_2
**D-Asn-2**			29	4.42, m; 4.36, m	63.9, CH_2	47	1.47[j], m	28.5, CH
14	5.52, m	50.4, CH	30		173.8[c], C	48	0.84, d (6.3)	23.1, CH_3
15	3.63, m; 3.19[e], m	38.3, CH_2	28-NH	8.87[d], br s		49	0.84, d (6.3)	23.1, CH_3
16		173.5[c], C	**L-Thr**			37-NH	8.15[d], br s	
17		172.9[c], C	31	4.93[h], m	59.7, CH			
14-NH	9.22[d], br s		32	4.98[h], m	66.7, CH			

[a, b, e, g, h, i, j, k]Overlapped. [c, d, f]Assignments may be interchanged.

Table S5. The ^{1}H (400 MHz) and ^{13}C NMR (100 MHz) data of compound **7** in pyridine-d_5.

				**7**				
position	δ_H, mult.(J in Hz)	δ_C, type	position	δ_H, mult.(J in Hz)	δ_C, type	position	δ_H, mult.(J in Hz)	δ_C, type
**L-Asn-1**			16-NH_2	8.39, br s; 7.90, br s		33	1.35^g, m	21.1, CH_3
1	5.30^a, m	53.1, CH	**L-Pro**			34		172.0, CH
2	3.06^b, m; 3.02^b, m	37.5, CH_2	18	4.73, m	62.5, CH	31-NH	8.39, br s	
3		173.2, C	19	2.12, m	30.1^c, CH_2	**D-β-AA**		
4		172.89, C	20	1.88, m; 1.66^d, m	25.3, CH_2	35		173.2, C
1-NH	8.99, br s		21	4.23, m; 4.04, m	48.9, CH_2	36	2.64^f, m; 2.44, m	42.2, CH_2
3-NH_2	8.56, br s; 7.85, br s		22		172.8, C	37	4.62, m	47.8, CH
**D-Tyr**			**L-Glu**			38	1.66^d, m; 1.47^h, m	35.9, CH_2
5	5.31^a, m	56.2, CH	23	4.87^e, m	55.6, CH	39	1.34^g, m	26.4, CH_2
6	3.72, m;<br>3.39, m	36.8, CH_2	24	2.80, m; 2.67^f, m	28.1, CH_2	40	1.21-1.15^i	29.9^c, CH_2
7		129.0, C	25	3.00^b, m; 2.97^b, m	32.0, CH_2	41	1.21-1.15^i	30.0^c, CH_2
8, 12	7.49, d (7.4)	131.6, CH	26		175.9, C	42	1.21-1.15^i	30.1^c, CH_2
9, 11	7.08, d (7.4)	116.4, CH	27		171.5, C	43	1.21-1.15^i	30.1^c, CH_2
10		157.8, C	23-NH	8.11,br s		44	1.21-1.15^i	30.2^c, CH_2
13		174.0, C	**D-Ser**			45	1.21-1.15^i	30.2^c, CH_2
5-NH	9.66, br s		28	4.88^e, m	58.3, CH	46	1.21-1.15^i	30.2^c, CH_2
**D-Asn-2**			29	4.37, m	63.8, CH_2	47	1.21-1.15^i	30.6^c, CH_2
14	5.42, m	50.6, CH	30		174.0,C	48	1.45^h, m	23.2, CH_2
15	3.56, m; 3.21, m	38.2, CH_2	28-NH	8.78,br s		49	0.88, t (7.4)	14.4, CH_3
16		173.5, C	**L-Thr**			37-NH	8.11, br s	
17		172.93,C	31	4.85^e, m	59.7, CH			
14-NH	8.92, br s		32	4.96, m	66.5, CH			

^{a,b,c,d,e,f,g,h,i} Overlapped. ^{c} Assignments may be interchanged.

Table S6. The ^{1}H (400 MHz) and ^{13}C NMR (100 MHz) data of compound **8** pyridine-d_5.

				**8**				
position	δ_H, mult.(J in Hz)	δ_C, type	position	δ_H, mult.(J in Hz)	δ_C, type	position	δ_H, mult.(J in Hz)	δ_C, type
**L-Asn-1**			16-NH_2	8.60[d], br s; 7.94, br s		33	1.37[i], m	21.0, CH_3
1	5.32[a], m	53.2, CH	**L-Pro**			34		171.5[c], CH
2	3.14[b], m; 3.10[b], m	37.2, CH_2	18	4.75, t (6.6)	62.3, CH	31-NH	8.60[d], br s	
3		173.36[c], C	19	2.11, m	30.16[e], CH_2	**D-β-AA**		
4		172.2[c], C	20	1.87, m; 1.63[f], m	25.4, CH_2	35		173.44[c], C
1-NH	9.06[d], br s		21	4.20, m; 3.99, m	48.8, CH_2	36	2.66[h], m; 2.60[h], m	42.5, CH_2
3-NH_2	8.46[d], br s; 7.87, br s		22		173.1[c], C	37	4.65, m	48.0, CH
**D-Tyr**			**L-Glu**			38	1.61[f], m; 1.47[j], m	36.2, CH_2
5	5.32[a], m	56.4, CH	23	4.94[g], m	55.7, CH	39	1.32[i], m	26.5, CH_2
6	3.72, m;<br>3.46, m	36.8, CH_2	24	2.77, m; 2.66[h], m	28.0, CH_2	40	1.22-1.16[k]	30.0[e], CH_2
7		129.2, C	25	2.97, m; 2.95, m	32.1, CH_2	41	1.22-1.16[k]	30.23[e], CH_2
8, 12	7.49, d (7.8)	131.6, CH	26		175.9, C	42	1.22-1.16[k]	30.23[e], CH_2
9, 11	7.09, d (7.8)	116.5, CH	27		171.5, C	43	1.22-1.16[k]	30.31[e], CH_2
10		157.8, C	23-NH	8.34[d], br s		44	1.22-1.16[k]	30.31[e], CH_2
13		173.9[c], C	**D-Ser**			45	1.22-1.16[k]	30.33[e], CH_2
5-NH	9.58, br s		28	4.95[g], m	58.1, CH	46	1.22-1.16[k]	30.6[e], CH_2
**D-Asn-2**			29	4.43, m; 4.38, m	63.8, CH_2	47	1.22-1.16[k]	38.2, CH_2
14	5.46, m	50.6, CH	30		173.44[c], C	48	1.47[j], m	28.5, CH
15	3.57, m;<br>3.18[b], m	37.6, CH_2	28-NH	8.91[d], br s		49	0.84, d (6.5)	23.1, CH_3
16		173.44[c], C	**L-Thr**			50	0.84, d (6.5)	23.1, CH_3
17		173.1[c], C	31	4.92[g], m	59.8, CH	37-NH	8.32[d], br s	
14-NH	9.25[d], br s		32	5.00[g], m	67.0, CH			

[a, b, f, g, h, i, j, k] Overlapped. [c, d, e] Assignments may be interchanged.

Table S7. The ^{1}H (400 MHz) and ^{13}C NMR (100 MHz) data of compound **9** pyridine-d_5.

				**9**				
position	δ_H, mult.(J in Hz)	δ_C, type	position	δ_H, mult.(J in Hz)	δ_C, type	position	δ_H, mult.(J in Hz)	δ_C, type
**L-Asn-1**			16-NH_2	8.61[c], br s; 7.94, br s		33	1.38[i], m	20.9, CH_3
1	5.32[a], m	53.1, CH	**L-Pro**			34		171.4[c], CH
2	3.13[b], m; 3.08[b], m	37.1, CH_2	18	4.75, t (6.6)	62.2, CH	31-NH	8.61[d], br s	
3		173.3[c], C	19	2.12, m	30.0[e], CH_2	**D-β-AA**		
4		172.5[c], C	20	1.88, m; 1.64[f], m	25.3, CH_2	35		173.0, C
1-NH	9.01[d], br s		21	4.28, m; 4.05, m	48.7, CH_2	36	2.65[h], m; 2.55, m	42.4, CH_2
3-NH_2	8.43[d], br s; 7.86, br s		22		173. 6[c], C	37	4.65, m	48.0, CH
**D-Tyr**			**L-Glu**			38	1.64[f], m; 1.46, m	35.9, CH_2
5	5.32[a], m	56.3, CH	23	4.92[g], m	55.6, CH	39	1.38[i], m	26.4, CH_2
6	3.76, m; 3.47, m	36.6, CH_2	24	2.81, m; 2.68[h], m	27.8, CH_2	40	1.24-1.17[j]	29.8[e], CH_2
7		129.2, C	25	3.01[b], m; 2.99[b], m	32.4, CH_2	41	1.24-1.17[j]	29.8[e], CH_2
8, 12	7.50, d (7.9)	131.5, CH	26		175.8, C	42	1.24-1.17[j]	30.15[e], CH_2
9, 11	7.09, d (7.9)	116.4, CH	27		171.4, C	43	1.24-1.17[j]	30.15[e], CH_2
10		157.7, C	23-NH	8.25[d], br s		44	1.24-1.17[j]	30.15[e], CH_2
13		173.7[c], C	**D-Ser**			45	1.24-1.17[j]	30.21[e], CH_2
5-NH	9.63, br s		28	4.95[g], m	58.0, CH	46	1.24-1.17[j]	30.21[e], CH_2
**D-Asn-2**			29	4.43, m; 4.38, m	63.7, CH_2	47	1.24-1.17[j]	30.21[e], CH_2
14	5.46, m	50.4, CH	30		172.1[c], C	48	1.24-1.17[j]	31.9, CH_2
15	3.62, m; 3.21[b], m	37.6, CH_2	28-NH	8.88[d], br s		49	1.24, m	23.2, CH_2
16		173.3[c], C	**L-Thr**			50	0.87, t (6.4)	14.5, CH_3
17		173.2[c], C	31	4.90[g], m	59.8, CH	37-NH	8.23[d], br s	
14-NH	9.20[d], br s		32	4.96[g], m	66.9, CH			

[a, b, f, g, h, i, j]Overlapped. [c, d, e] Assignments may be interchanged.

Table S8. The ^{1}H (400 MHz) data of compound **10** pyridine-d_5.

		**10**			
position	δ_H, mult.(J in Hz)	position	δ_H, mult.(J in Hz)	position	δ_H, mult.(J in Hz)
**L-Asn-1**		19	2.12, m	36	2.64^{f}, m; 2.45, m
1	4.98^{a}, m	20	1.71^{d}, m; 1.65^{d}, m	37	4.64, m
2	3.09^{b}, m; 3.08^{b}, m	21	4.27, m; 4.05, m	38	1.59^{d}, m; 1.45, m
1-NH	9.00^{c}, br s	**L-Glu**		39	1.35^{g}, m
3-NH_2	8.42^{c}, br s; 7.88, br s	23	4.90^{e}, m	40	1.22-1.17^{h}
**D-Tyr**		24	2.82, m; 2.67^{f}, m	41	1.22-1.17^{h}
5	4.99^{a}, m	25	3.06^{b}, m; 3.02^{b}, m	42	1.22-1.17^{h}
6	3.74, m;<br>3.41, m	23-NH	8.15^{c}, br s	43	1.22-1.17^{h}
8, 12	7.50, d (8.2)	**D-Ser**		44	1.22-1.17^{h}
9, 11	7.08, d (8.2)	28	4.92^{e}, m	45	1.22-1.17^{h}
5-NH	9.74, br s	29	4.40, m; 4.37, m	46	1.22-1.17^{h}
**D-Asn-2**		28-NH	8.94^{c}, br s	47	1.22-1.17^{h}
14	5.70, m	**L-Thr**		48	1.25, m
15	3.58, m; 3.19, m	31	4.88^{e}, m	49	1.27, m;1.05, m
14-NH	9.05^{c}, br s	32	4.93^{e}, m	50	0.84, t (7.3)
16-NH_2	8.60^{c}, br s; 7.95, br s	33	1.35^{g}, d (6.3)	51	0.83, d (6.3)
**L-Pro**		31-NH	8.78^{c}, br s	37-NH	8.13^{c}, br s
18	4.75, t (6.8)	**D-β-AA**			

$^{a, b, d, e, f, g, h}$ Overlapped. ^{c} Assignments may be interchanged.

Figure S1. Nonribosomal peptides from marine-derived *Bacillus* species.

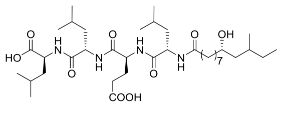

Gageopeptide A
(Antifungal, antibacterial)

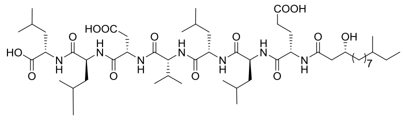

Gageosatin A
(Antifungal, antitumor)

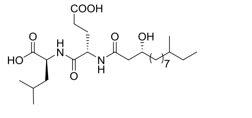

Gageotetrin A
(Antifungal, antibacterial)

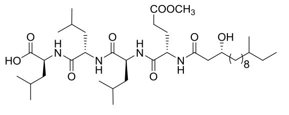

Gageopeptide B
(Antifungal, antibacterial)

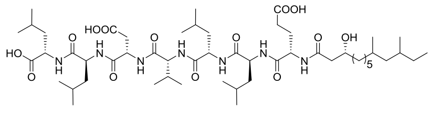

Gageosatin B
(Antifungal, antitumor)

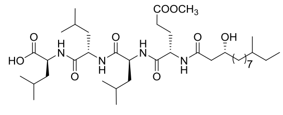

Gageotetrin B
(Antifungal, antibacterial)

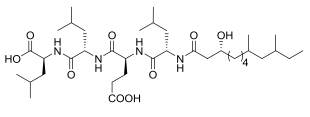

Gageopeptide C
(Antifungal, antibacterial)

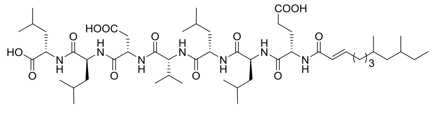

Gageosatin C
(Antifungal, antitumor)

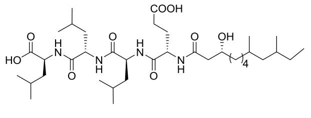

Gageotetrin C
(Antifungal, antibacterial)

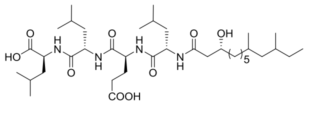

Gageopeptide D
(Antifungal, antibacterial)

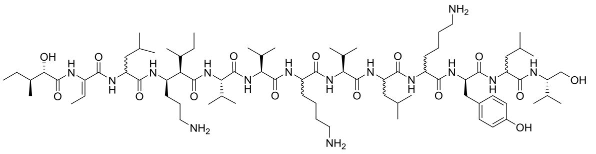

Bogorol A
(Antibacterial)

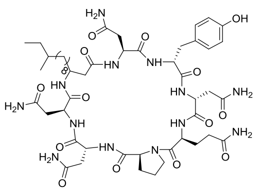

Mojavensin A
(Antifungal)

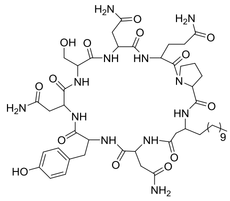

Marihysin A
(Antifungal)

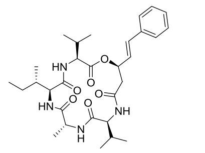

Turnagainolide A
(not detect)

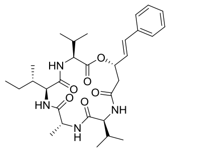

Turnagainolide B
(SHIP1 agonist)

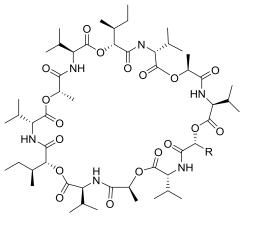

Bacillistatin 1 R = 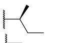

Bacillistatin 2 R = 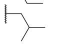
(Antitumor)

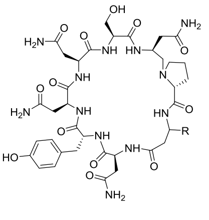

Mixirin A R = 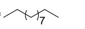

Mixirin B R = 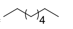

Mixirin C R = 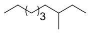
(Antitumor)

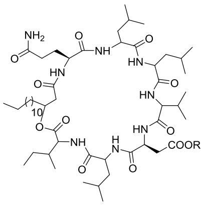

Halobacillin R = H
(Cytotoxicity)

Methyl halobacillin R = CH_3

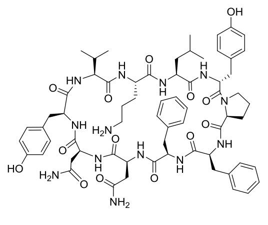

Loloatin A
(Antibacterial)

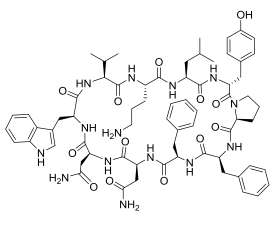

Loloatin B
(Antibacterial)

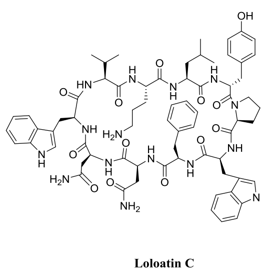

Loloatin C
(Antibacterial)

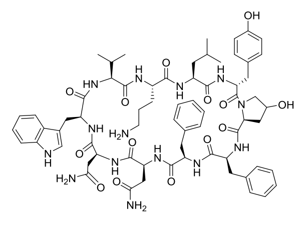

Loloatin D
(Antibacterial)

Figure S2. The agarose gel electrophoresis analysis of the 21 "positive" PCR products. Positive control: PCR product using the genomic DNA of one actinomycin producing strain as the template; negative control: PCR amplification without any genomic DNA used. The size of the expected PCR products is about 700 bp.

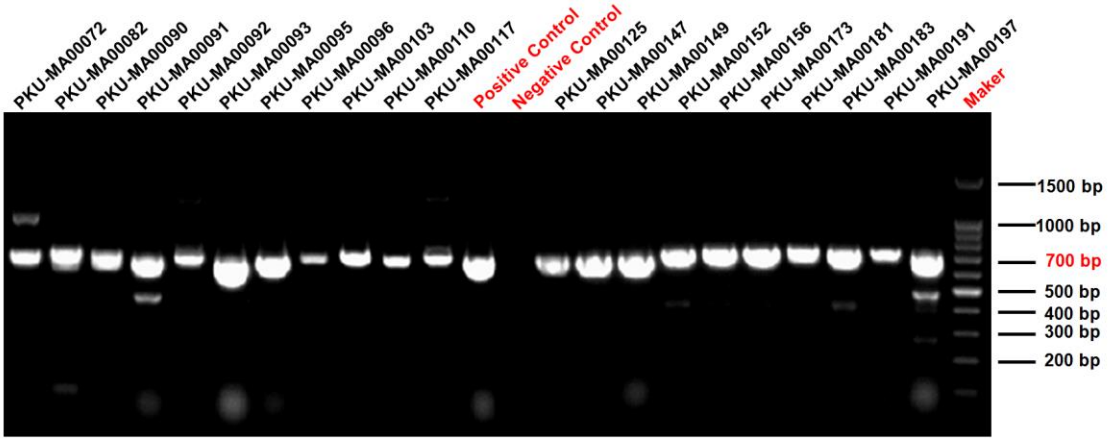

Figure S3. The phylogenetic analysis of strain PKU-MA00092 and strain PKU-MA00093 (labeled in red) based on comparison of 16S rRNA sequences. The sequence of 16S rRNA of *Streptomyces ovlivoverticillatus* HBUM175186 was used as an outgroup. The GenBank accession numbers are shown in parentheses.

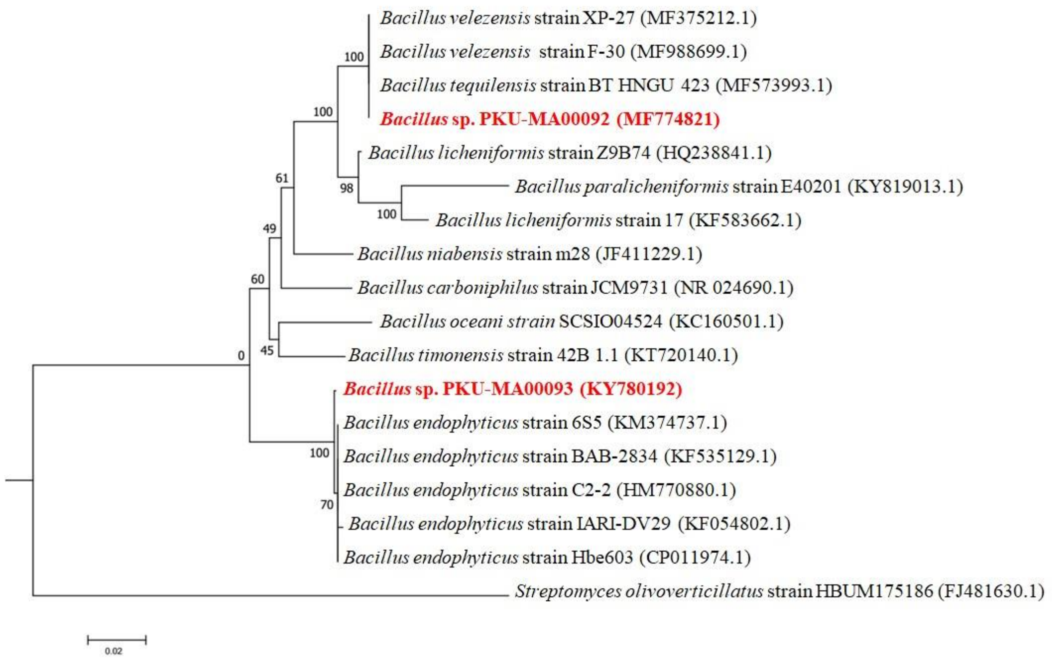

Figure S4. The ^{1}H NMR spectrum of compound **1** (DMSO-d_6, 400 MHz)

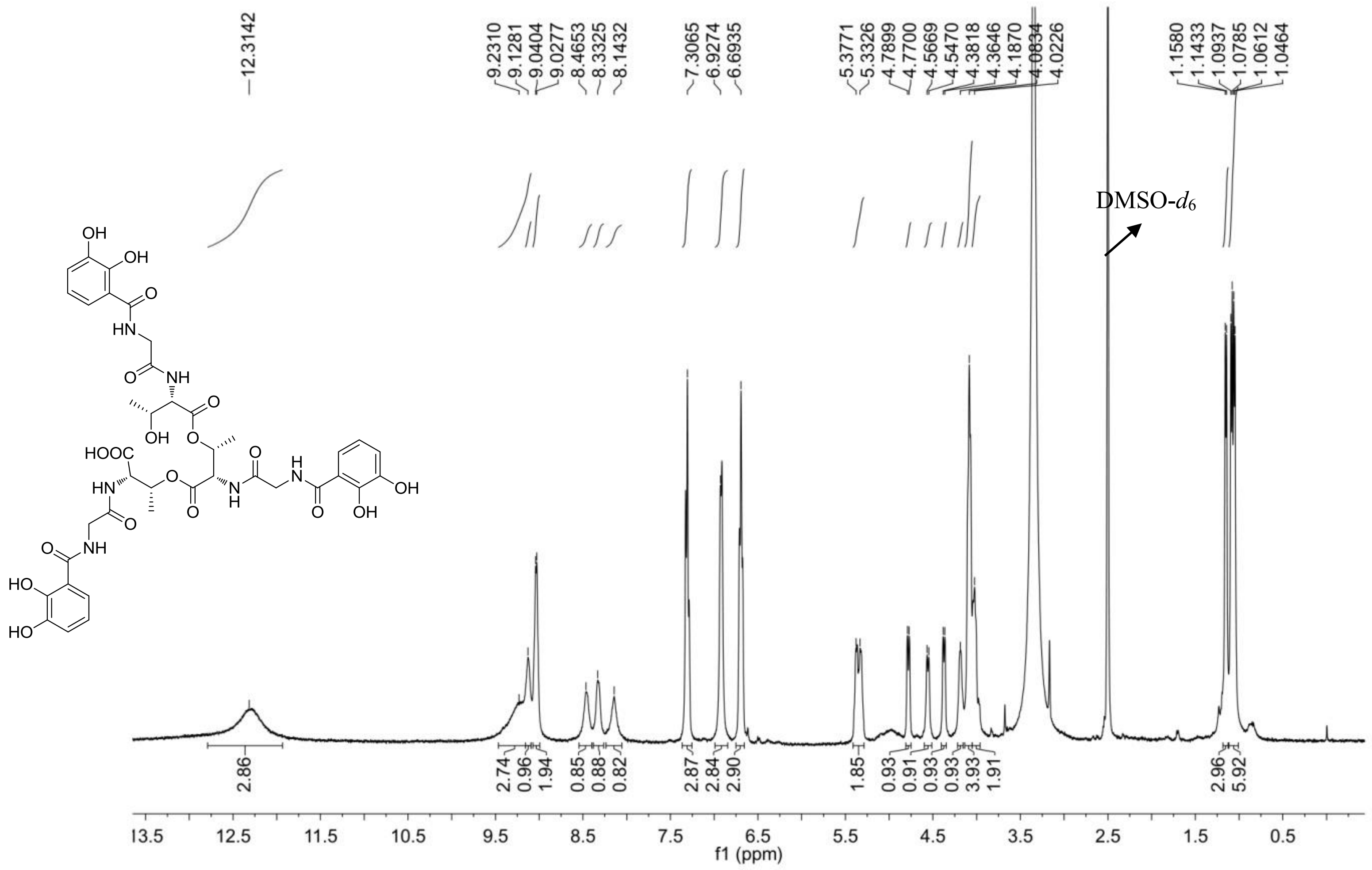

Figure S5. The COSY spectrum of compound **1** (DMSO-d_6, 400 MHz)

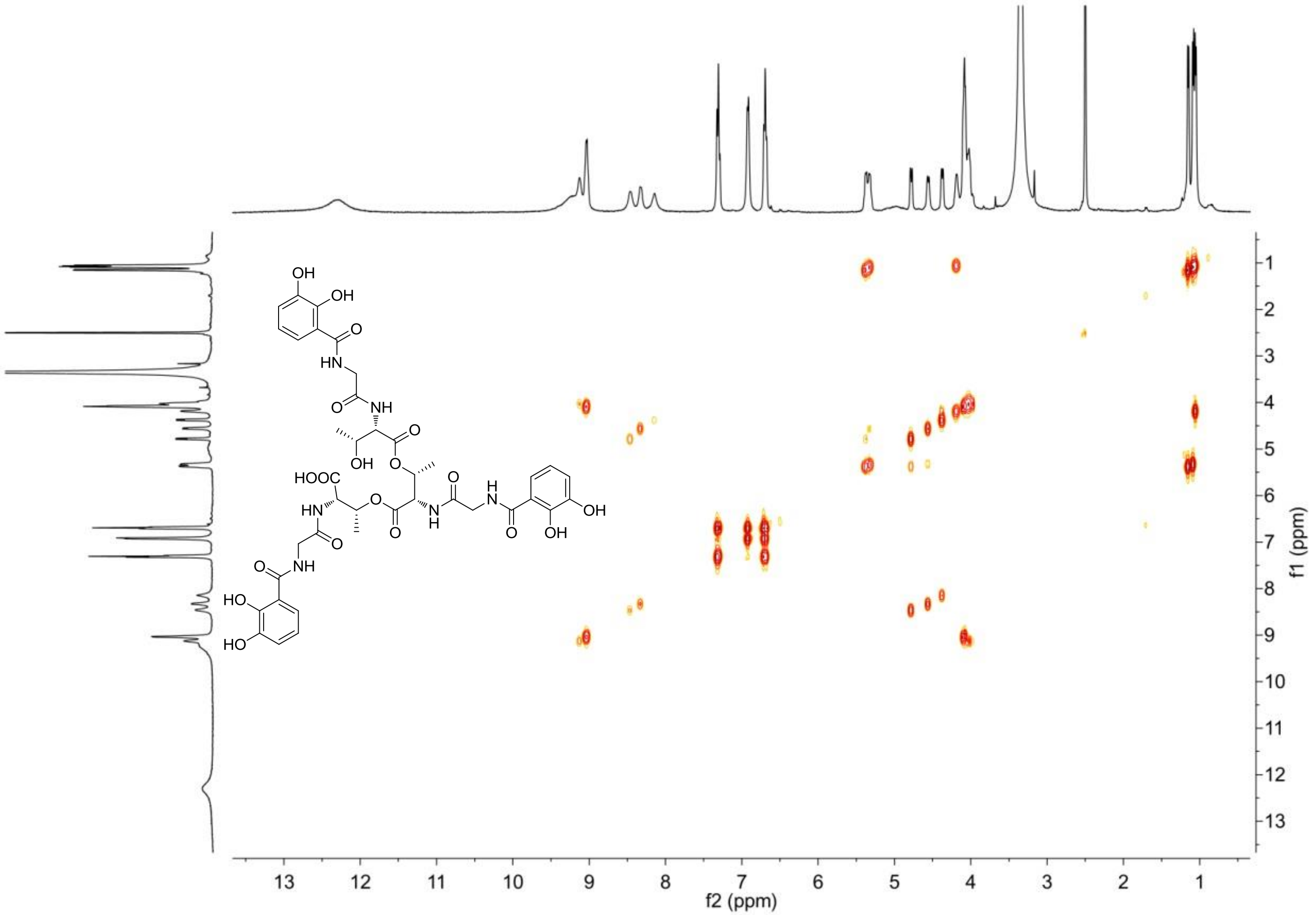

Figure S6. The ^{13}C NMR spectrum of compound **1** (DMSO-d_6, 100 MHz)

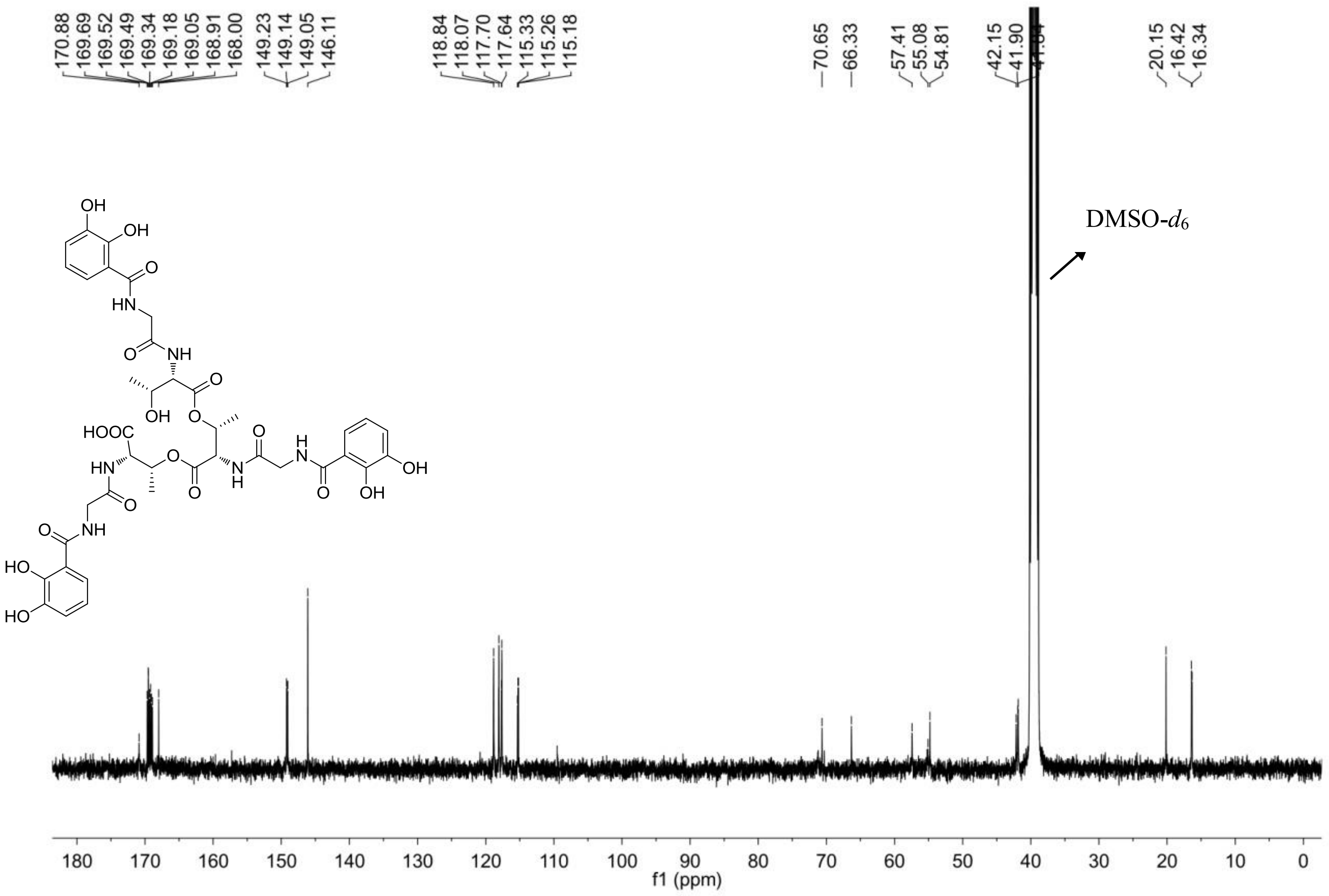

Figure S7. The HSQC spectrum of compound **1** (DMSO-d_6, 400 MHz)

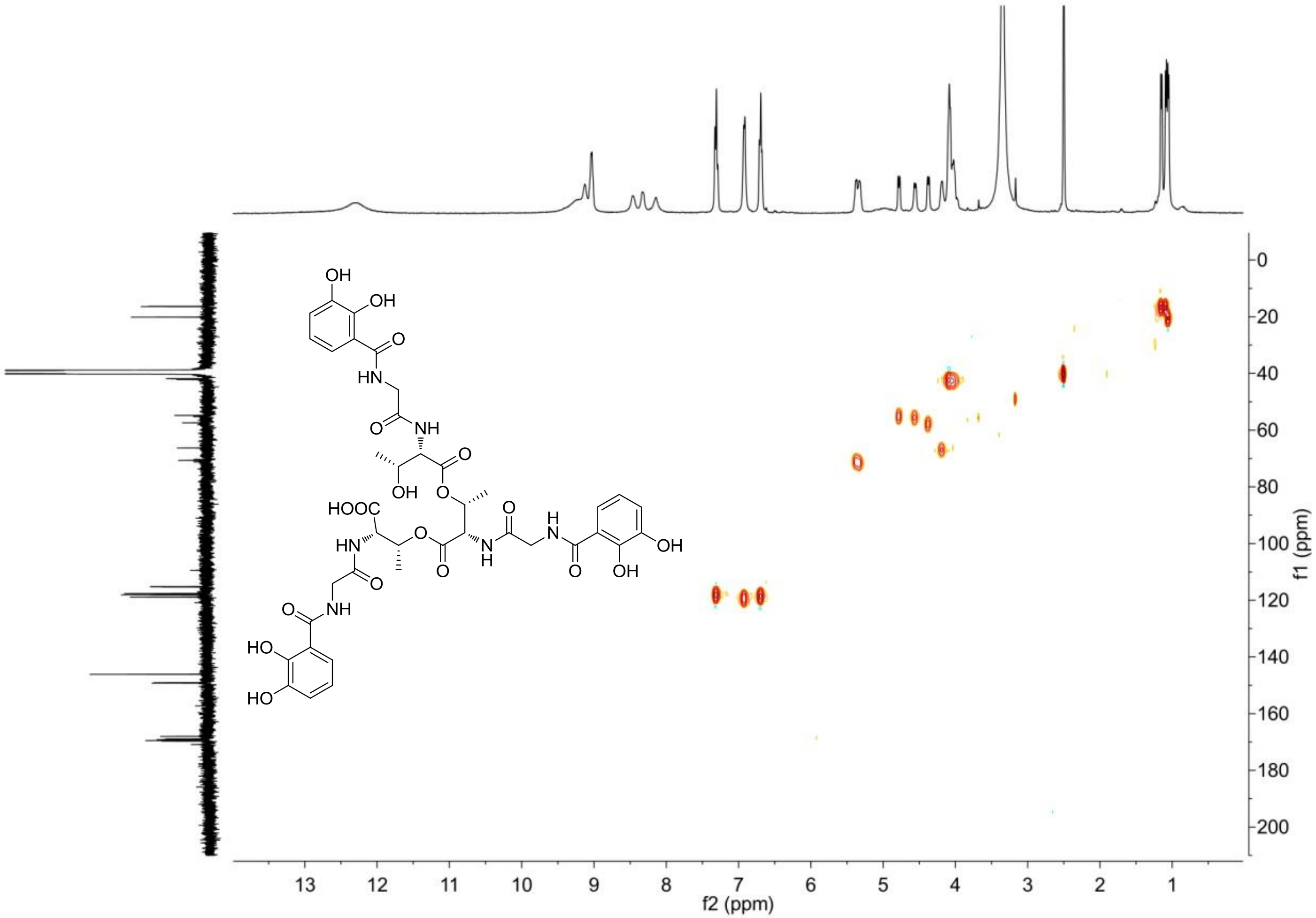

Figure S8. The HMBC spectrum of compound **1** (DMSO-d_6, 400 MHz)

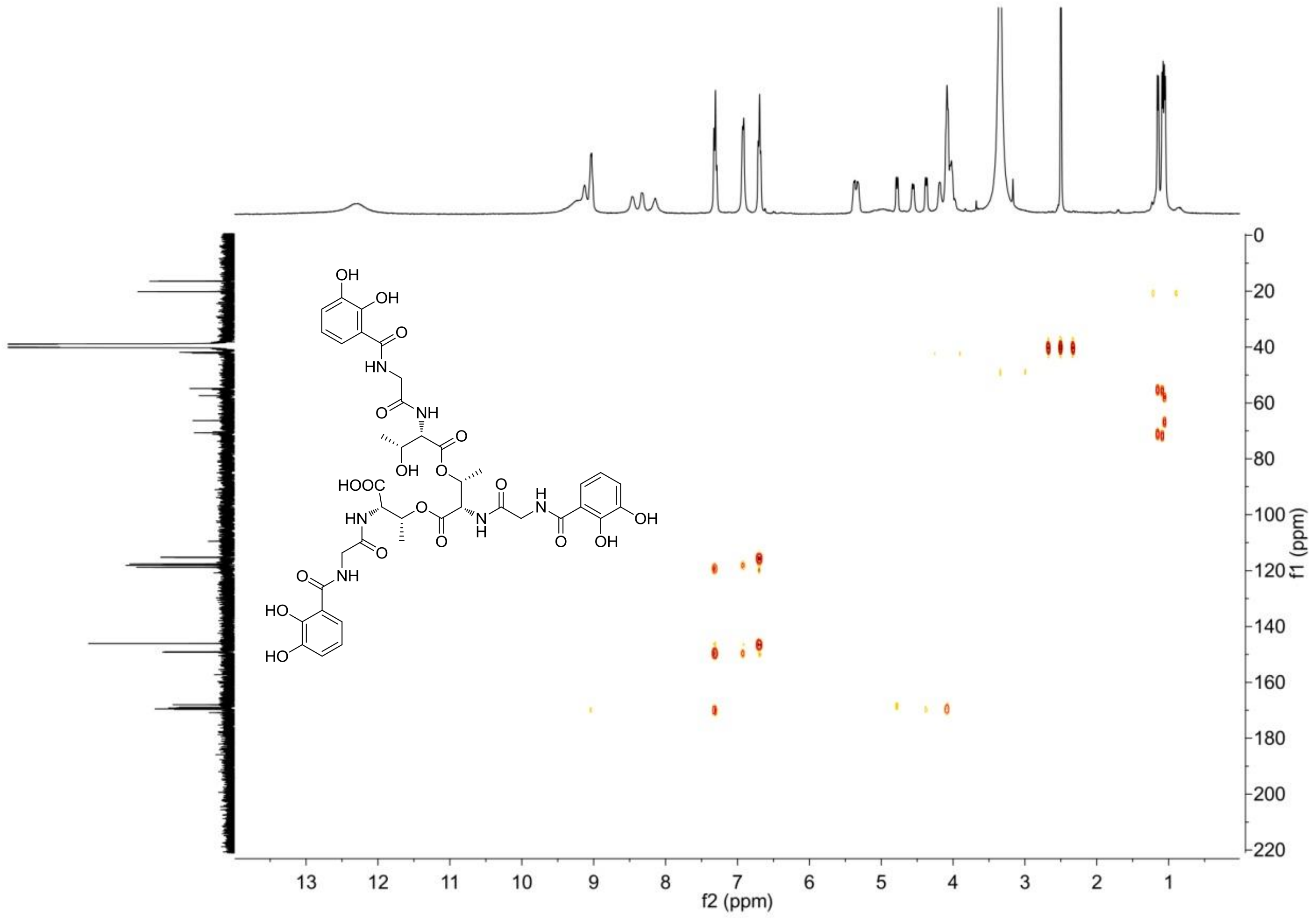

Figure S9. The HRESIMS spectrum of compound **1.**

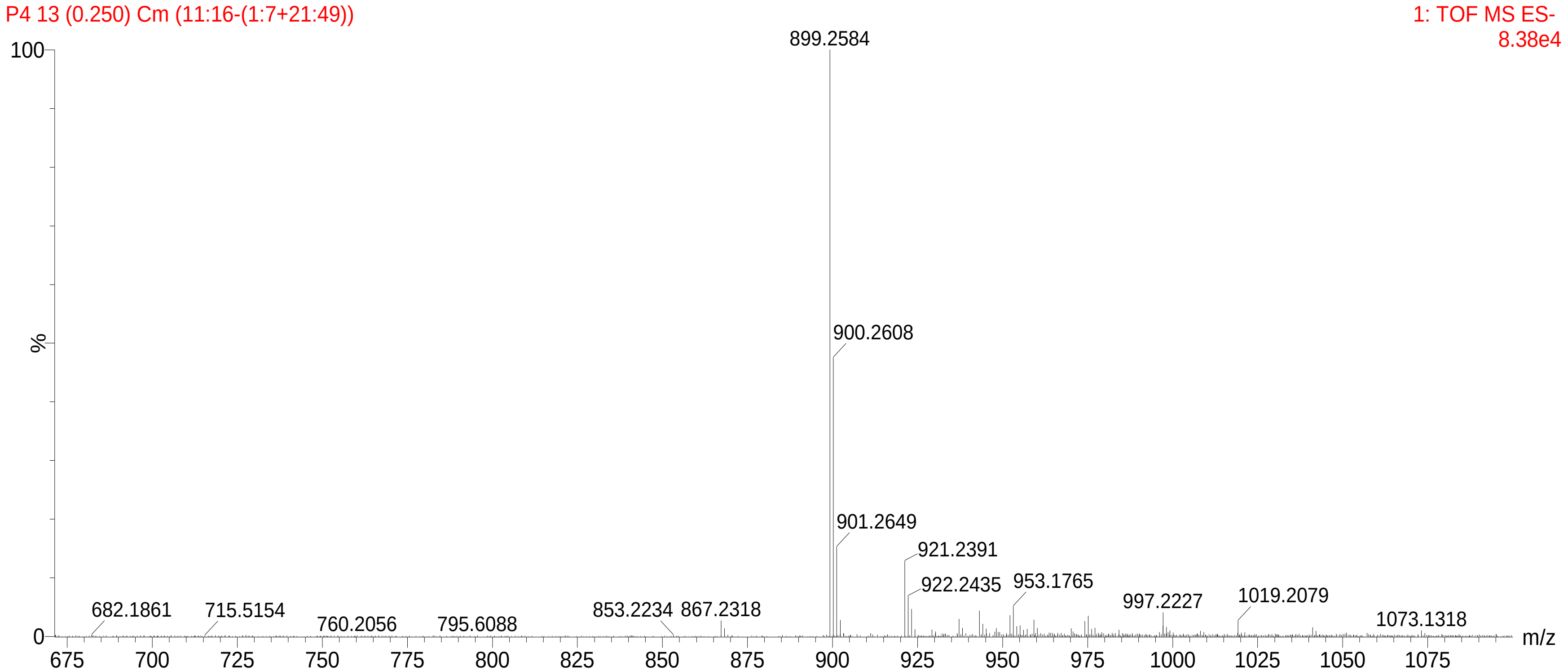

Mass	Calc.Mass	mDa	ppm	DBE	i-FIT	Norm	Conf(%)	Formula
899.2584	899.2583	0.1	0.1	21.5	93.7	1.140	31.99	$C_{39}H_{43}N_6O_{19}$

Figure S10. The IR spectrum of compound **1**.

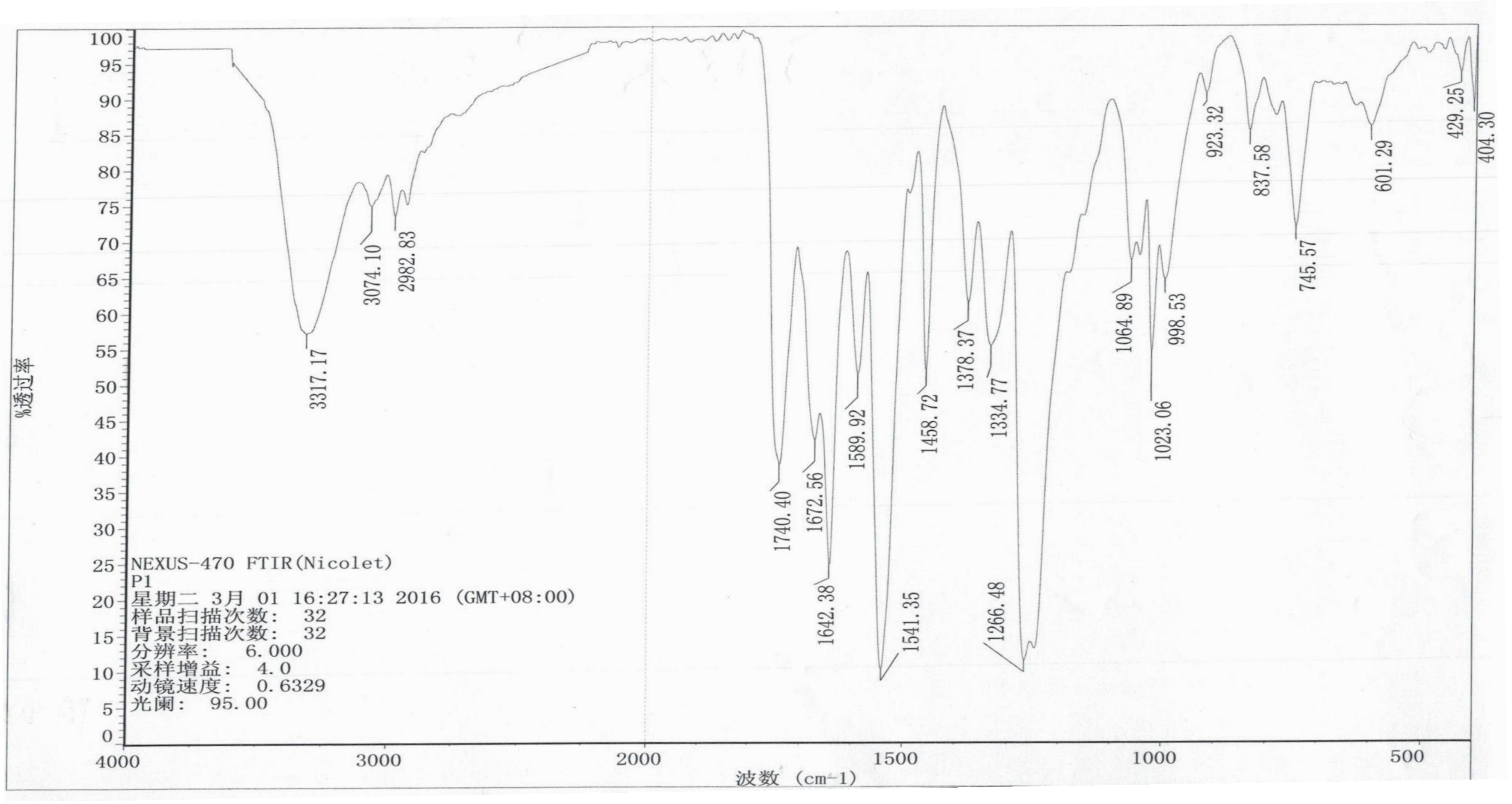

Figure S11. The MS/MS analysis of compound **1**.

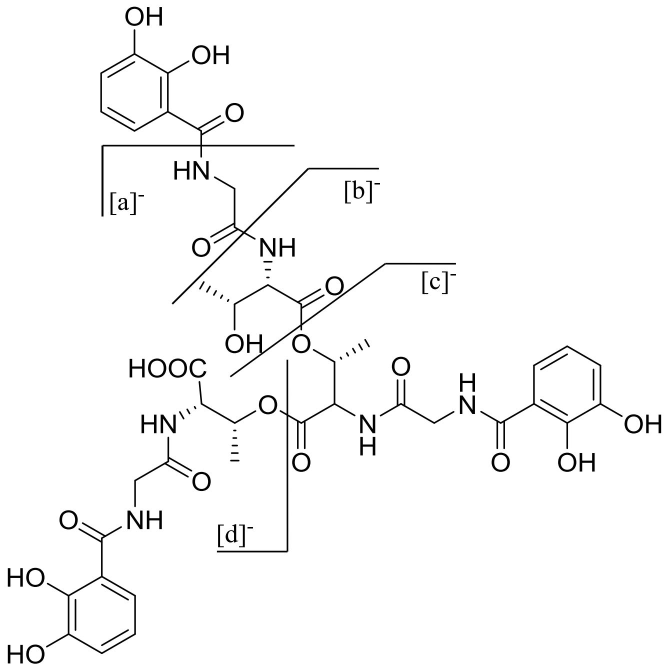

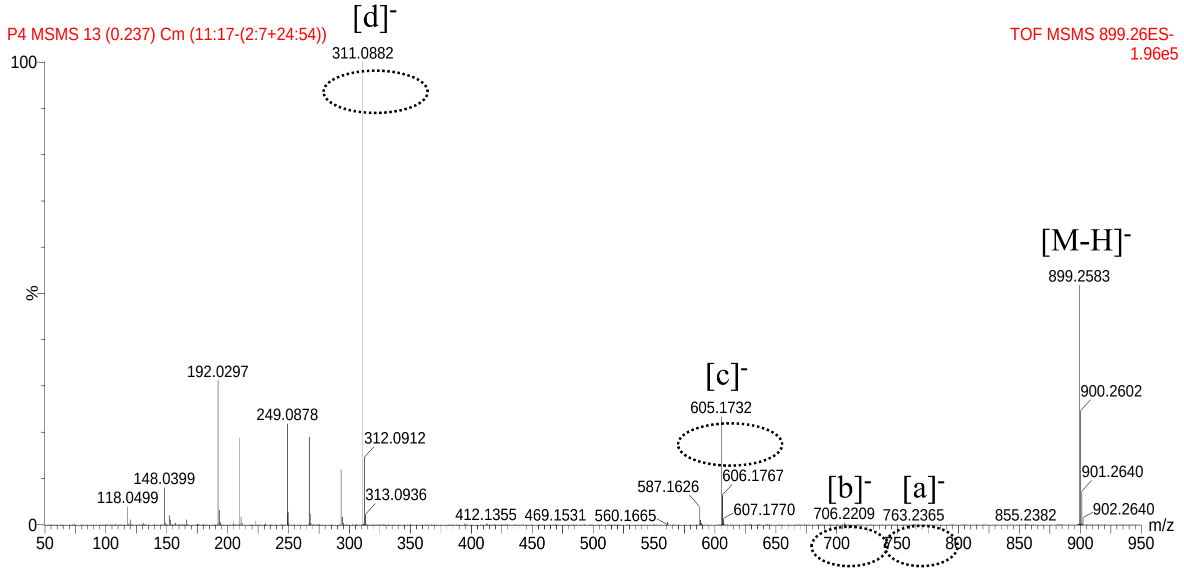

Figure S12. The ^{1}H NMR spectrum of compound **2** (DMSO-d_6, 400 MHz)

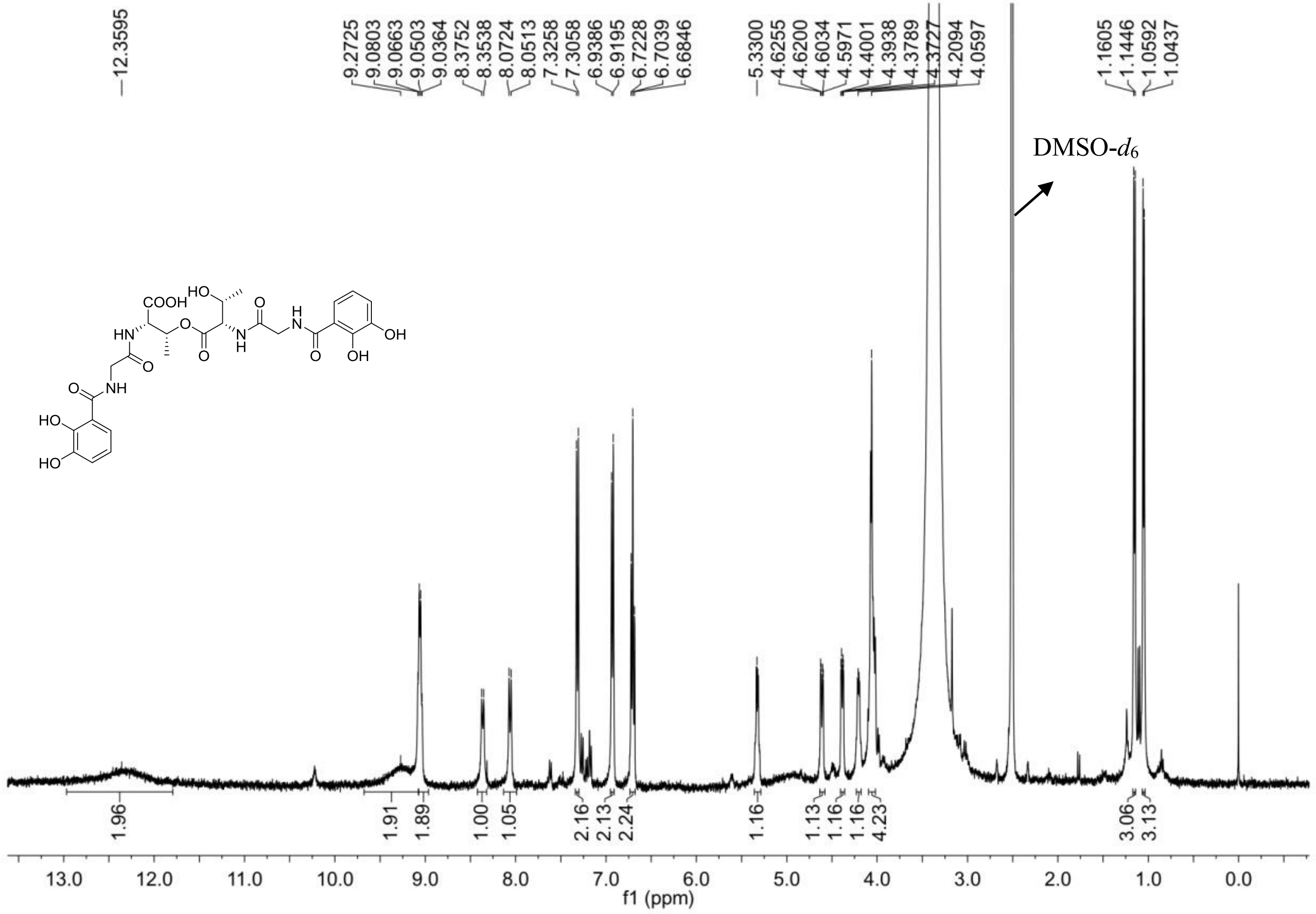

Figure S13. The COSY spectrum of compound **2** (DMSO-d_6, 400 MHz)

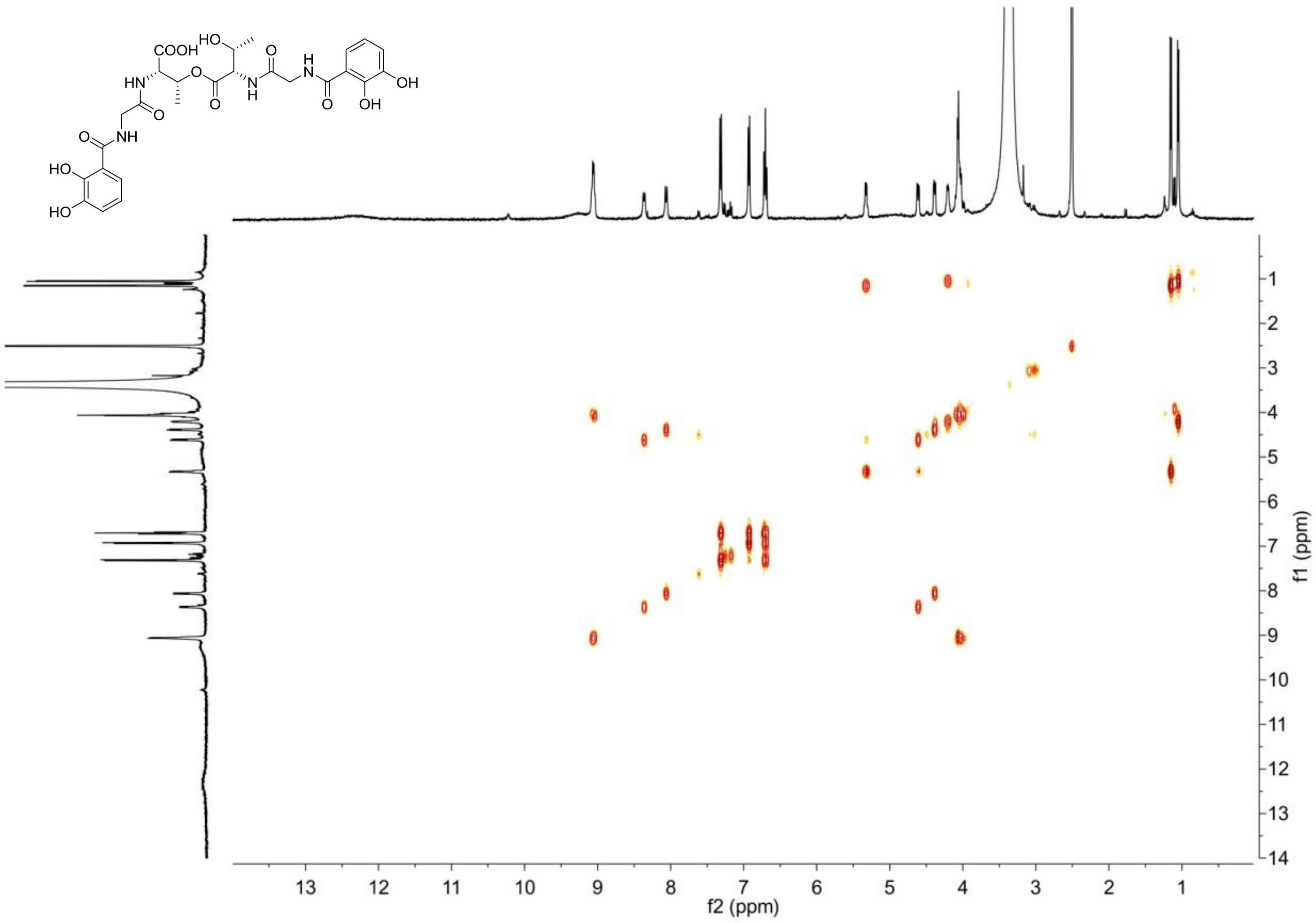

Figure S14. The APT spectrum of compound **2** (DMSO-d_6, 100 MHz)

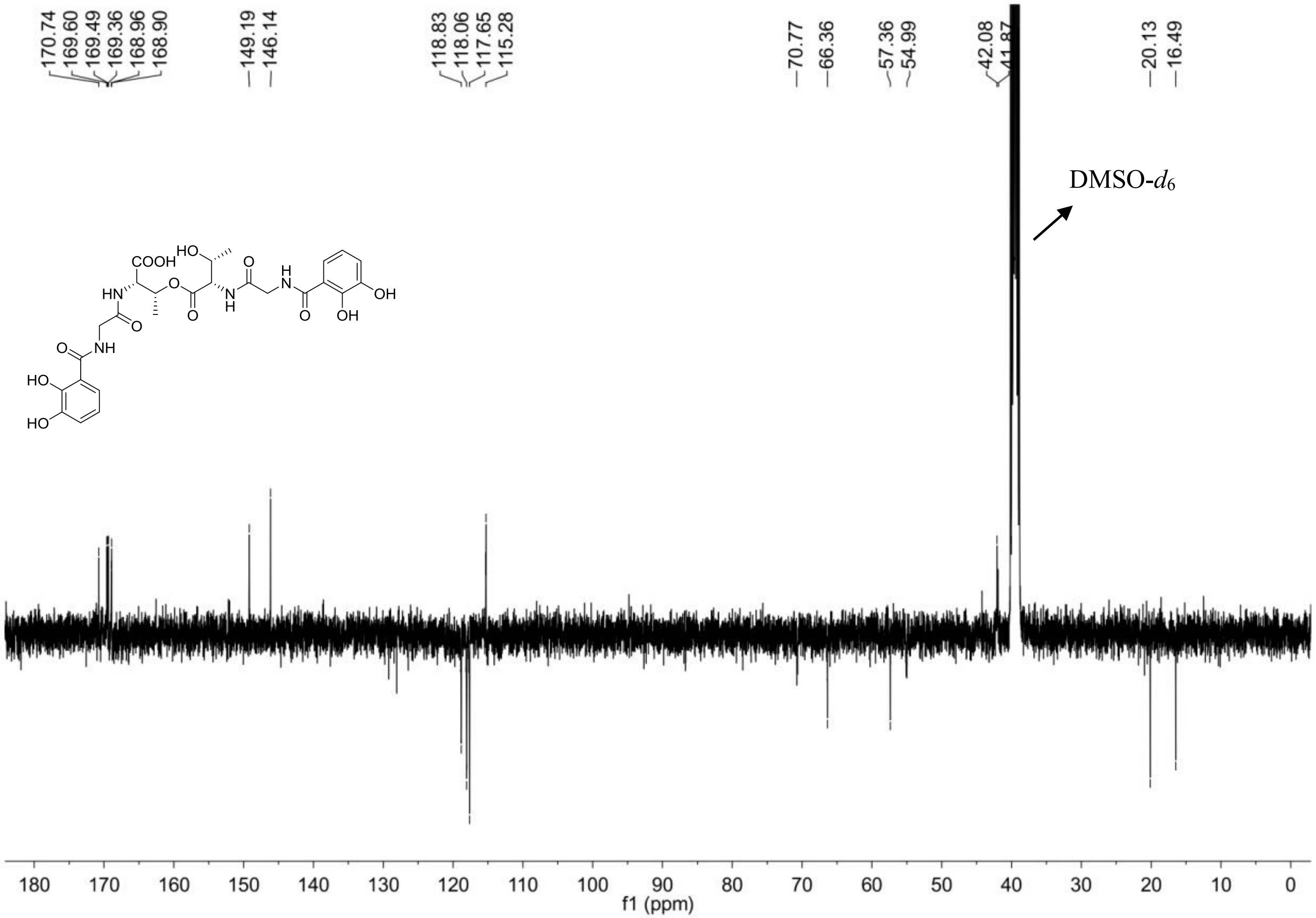

Figure S15. The HSQC spectrum of compound **2** (DMSO-d_6, 400 MHz)

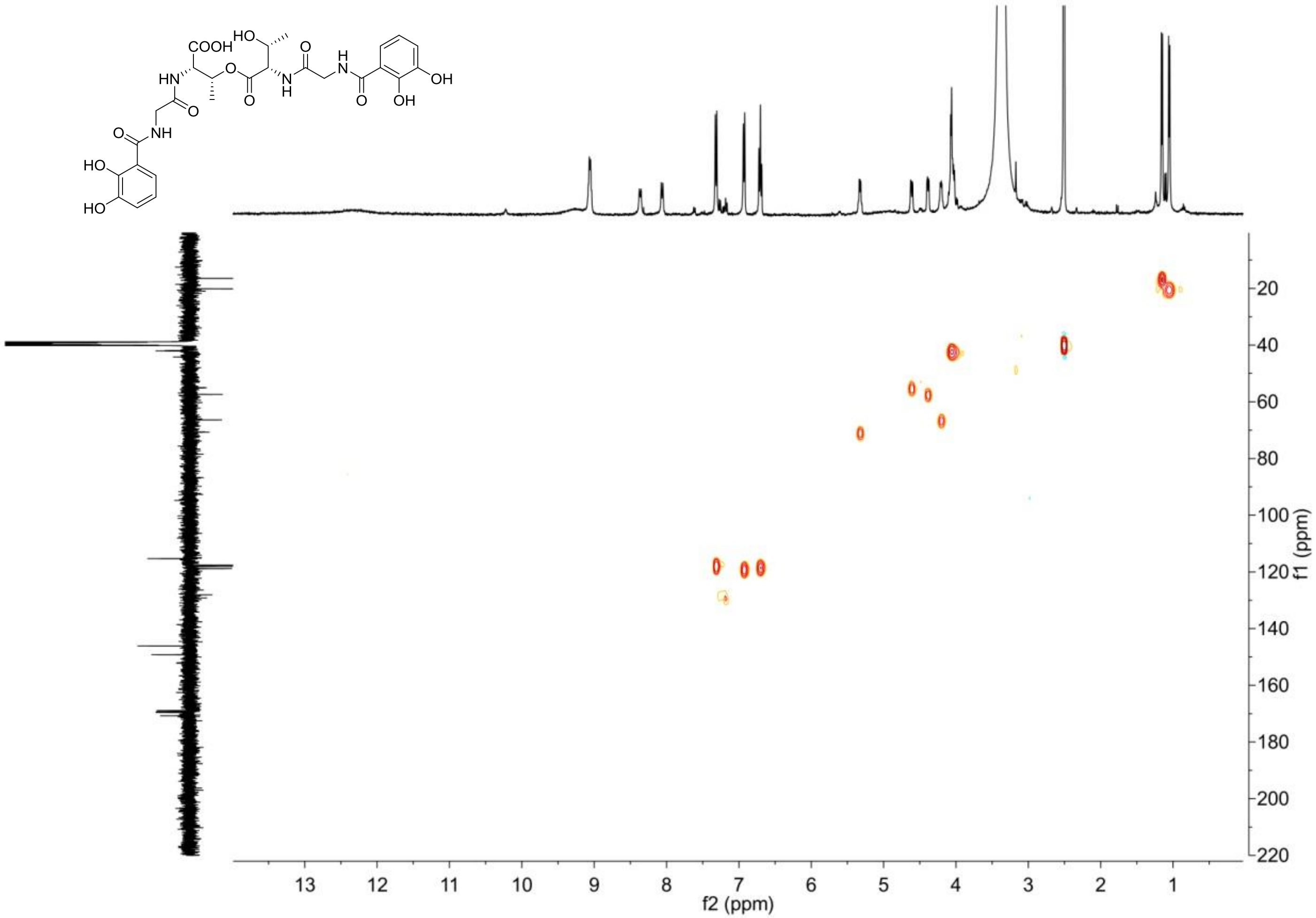

Figure S16. The HMBC spectrum of compound **2** (DMSO-d_6, 400 MHz).

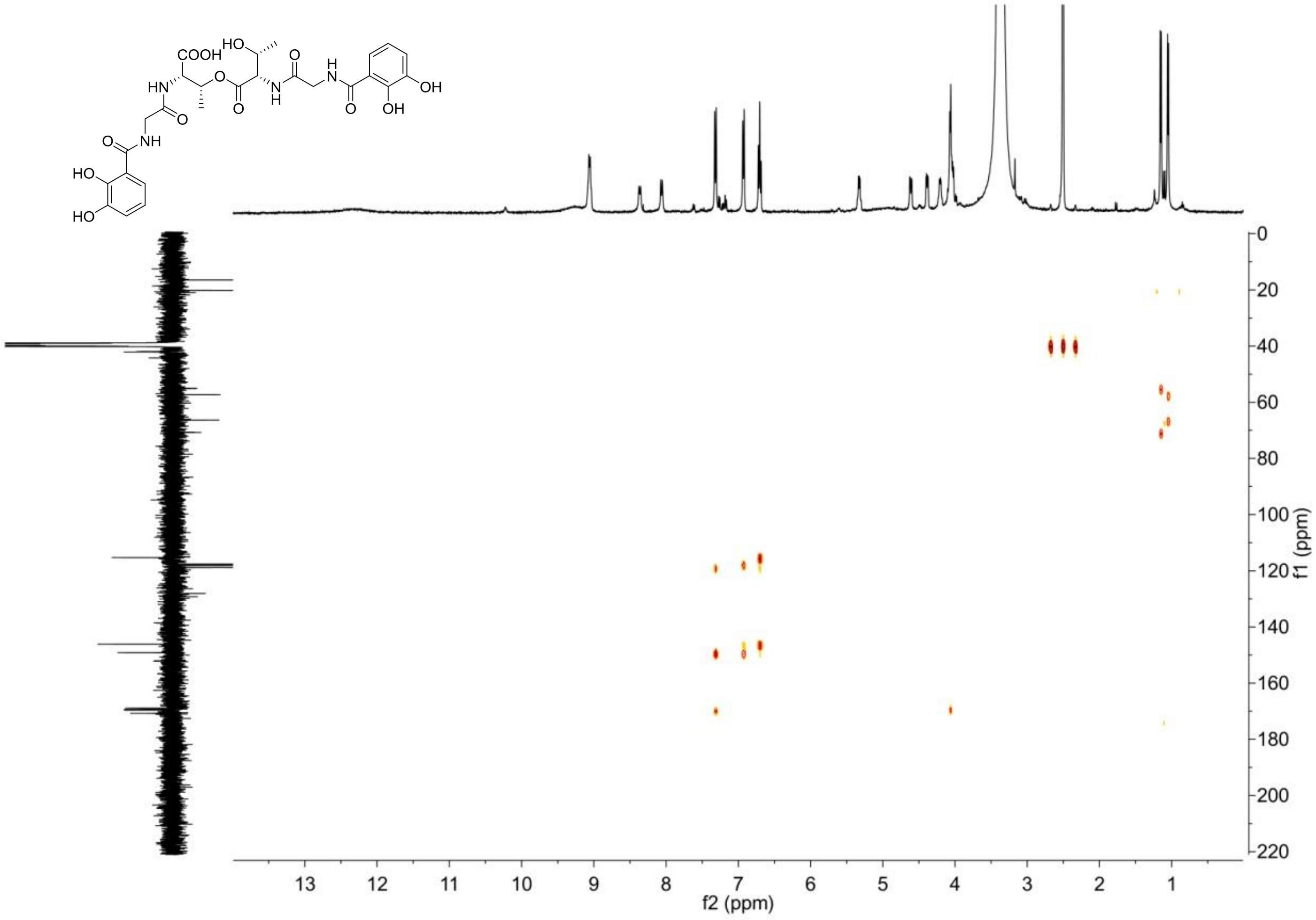

Figure S17. The HRESIMS spectrum of compound **2**.

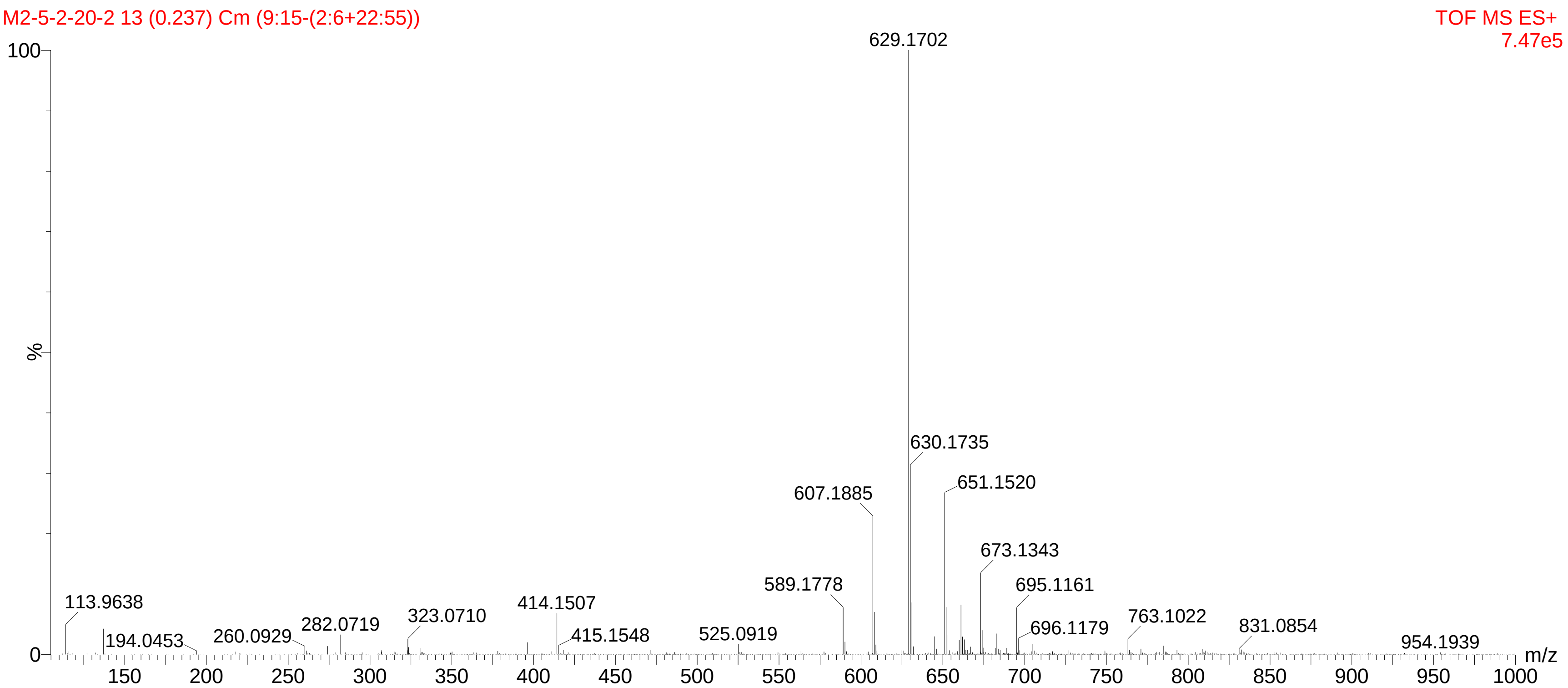

Mass	Calc.Mass	mDa	ppm	DBE	i-FIT	Norm	Conf(%)	Formula
629.1702	629.1707	-0.5	-0.8	13.5	147.1	0.974	37.76	$C_{26}H_{30}N_4O_{13}Na$

Figure S18. The IR spectrum of compound **2**.

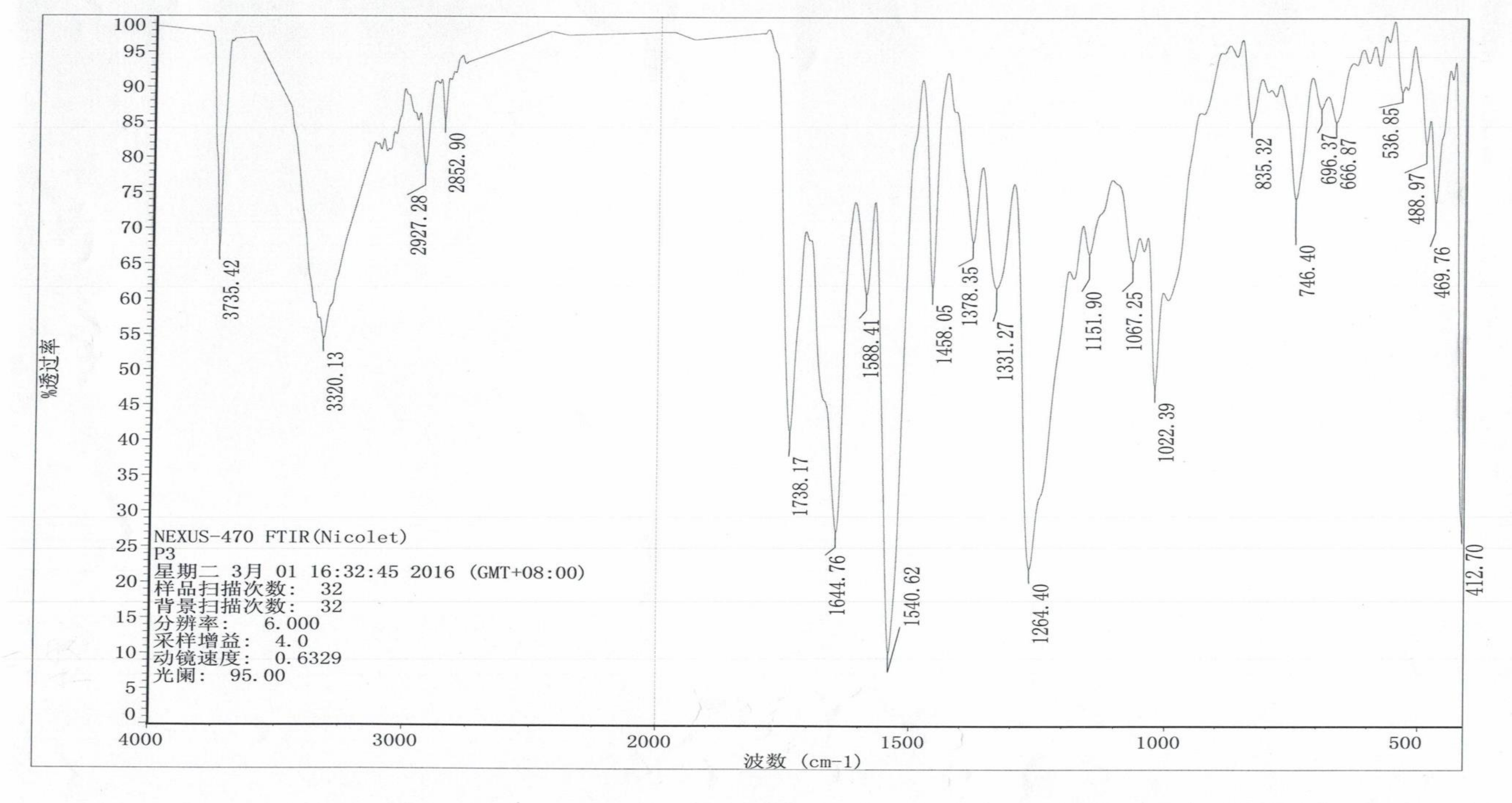

Figure S19. The MS/MS analysis of compound **2**.

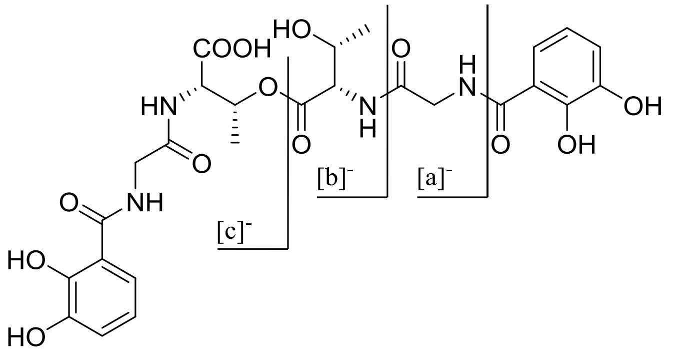

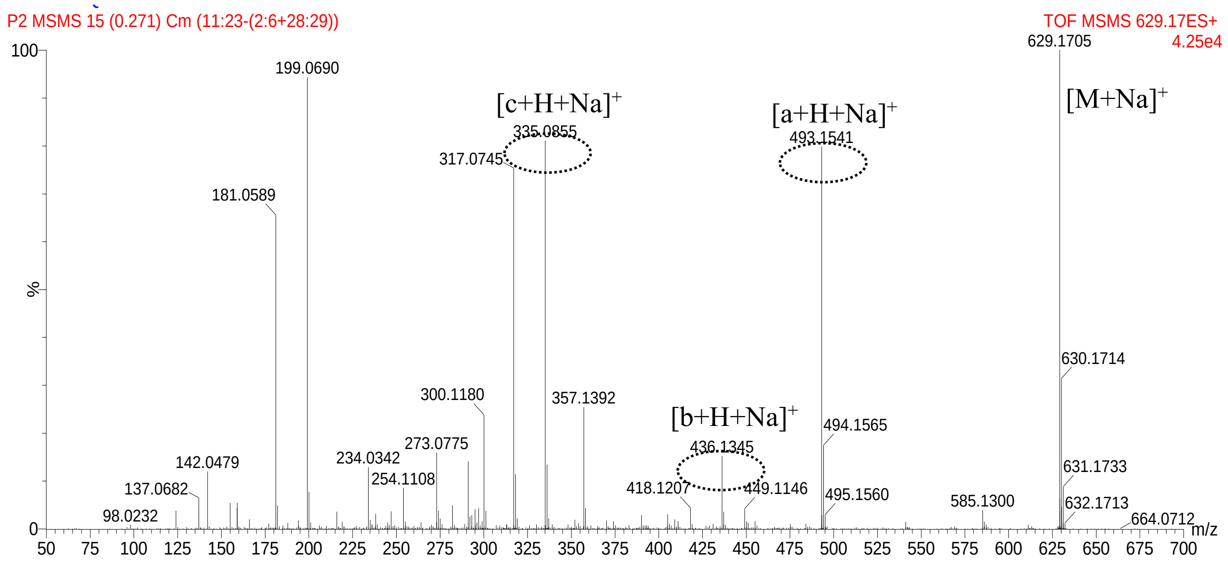

Figure S20. The ^{1}H NMR spectrum of compound **3** (DMSO-d_6, 400 MHz)

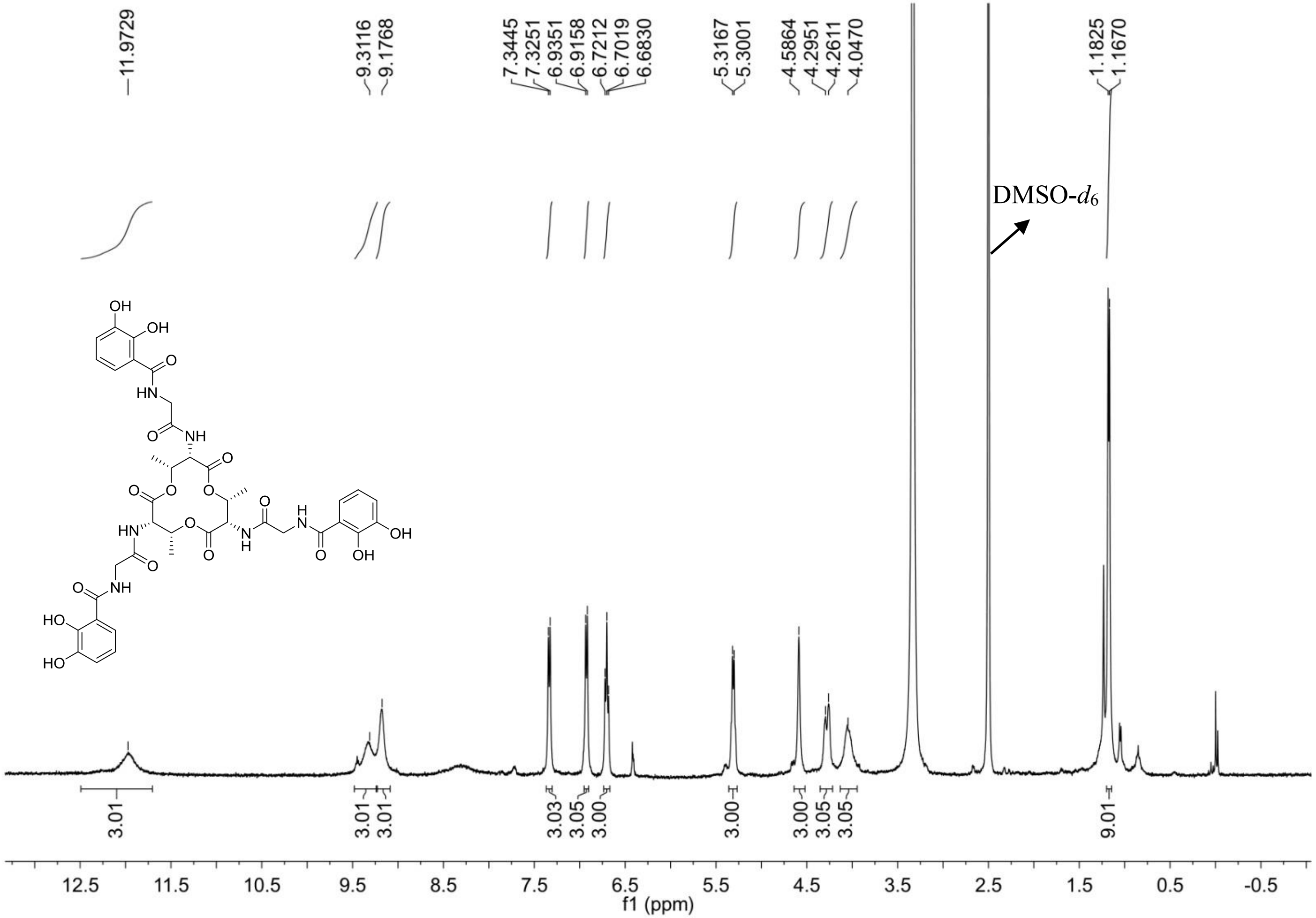

Figure S21. The APT spectrum of compound **3** (DMSO-d_6, 100 MHz)

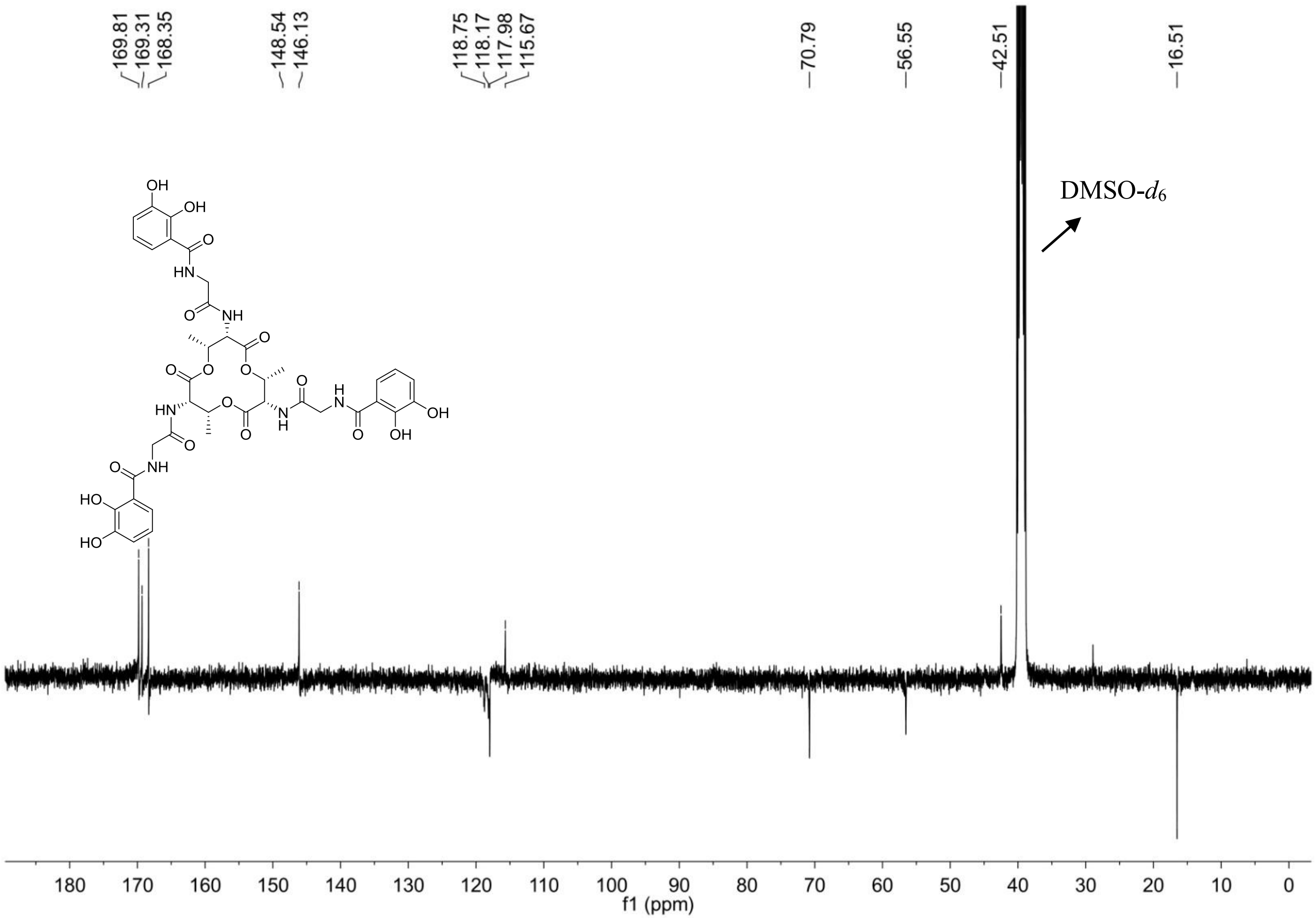

Figure S22. The HMBC spectrum of compound **3** (DMSO-d_6, 400 MHz).

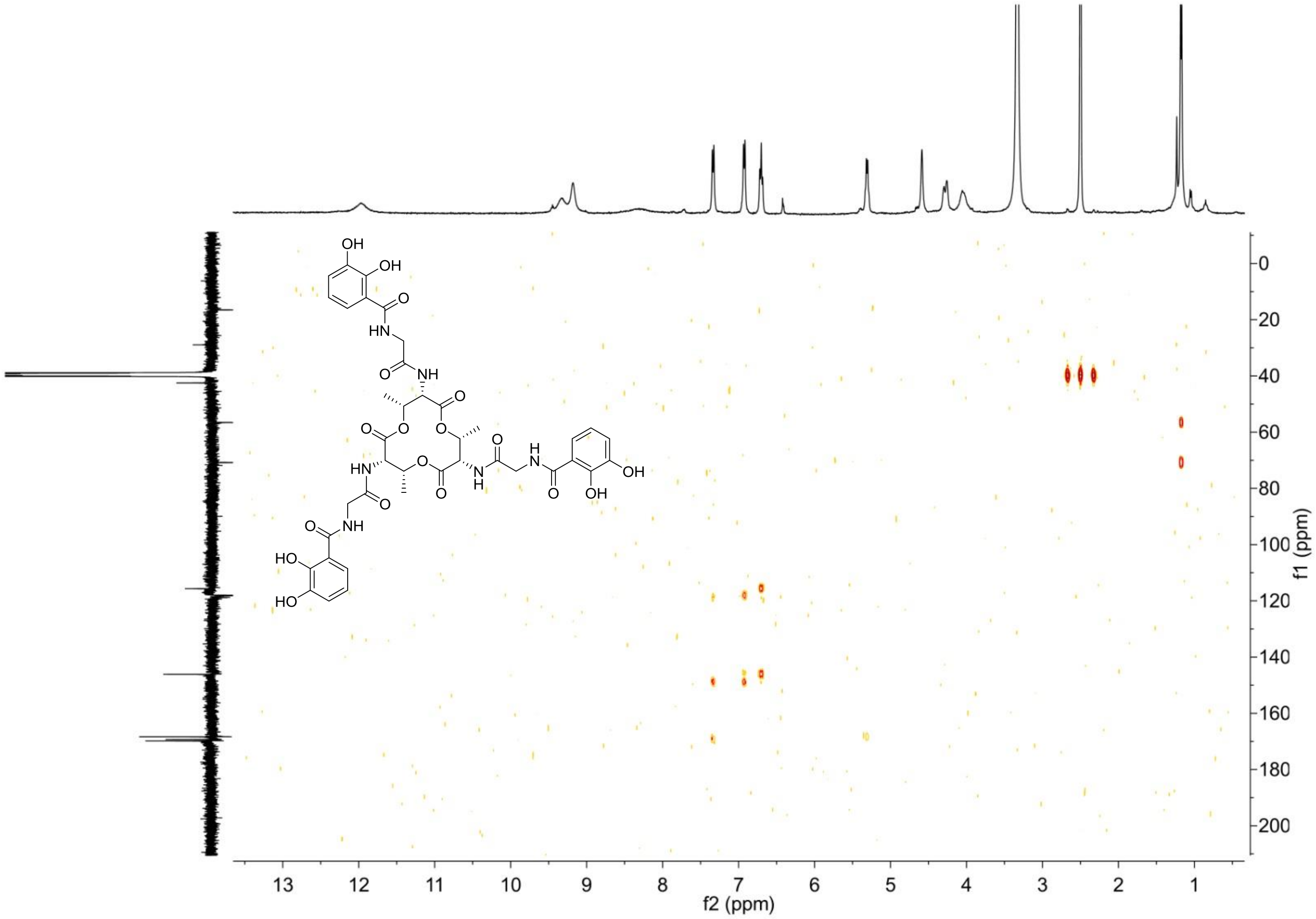

Figure S23. The HRESIMS spectrum of compound **3**.

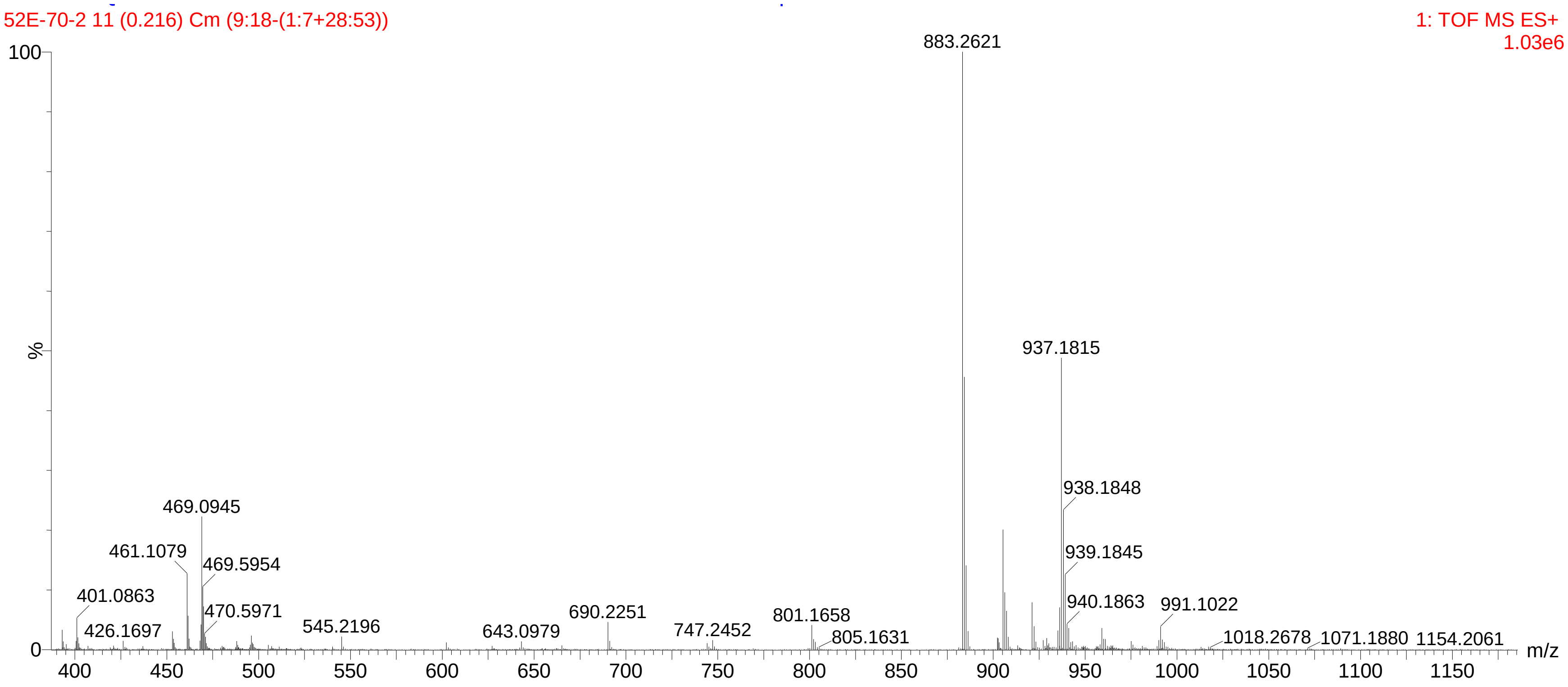

Figure S24. The ^{1}H NMR spectrum of compound **4** (DMSO-d_6, 400 MHz)

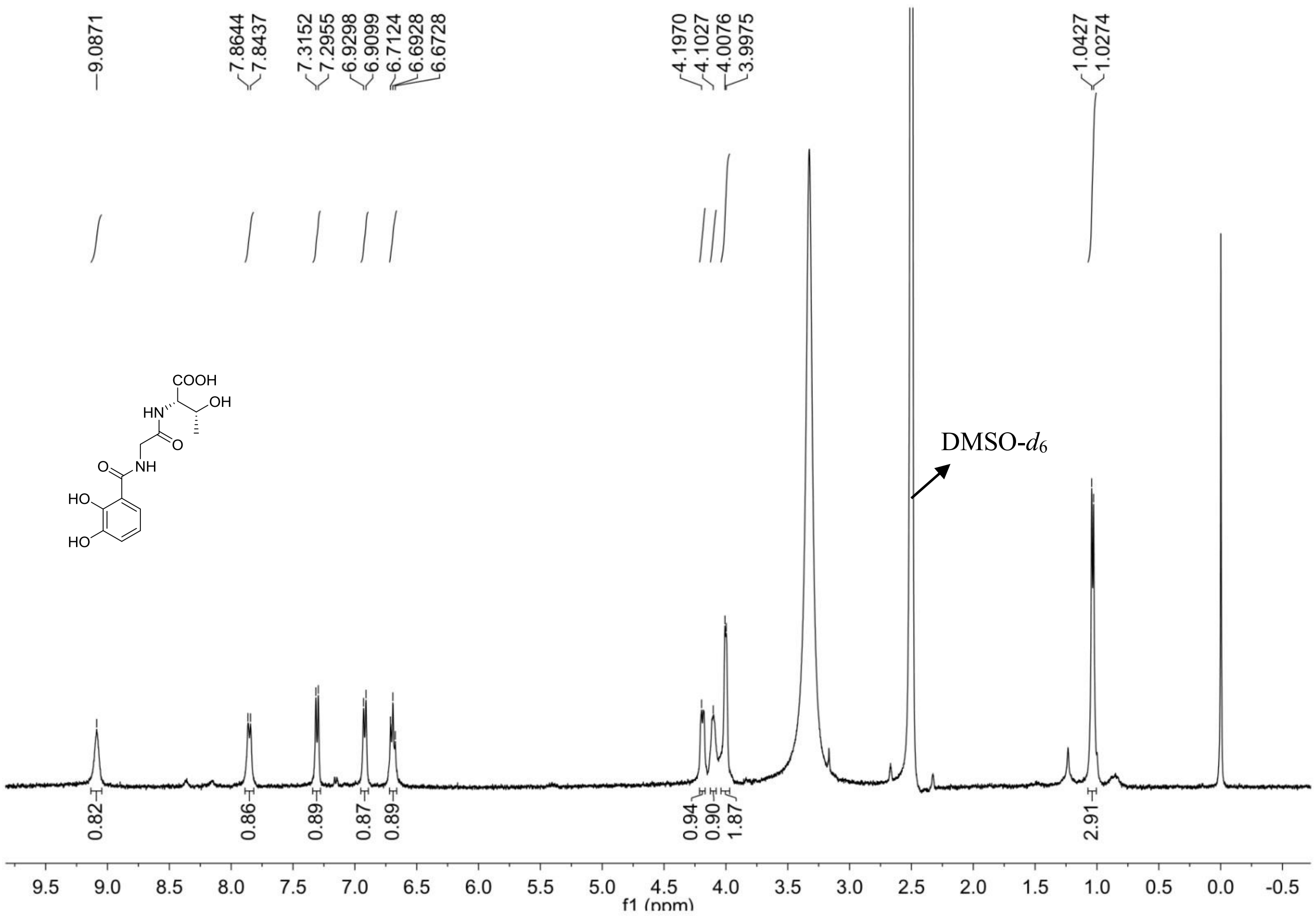

Figure S25. The ^{13}C NMR spectrum of compound **4** (DMSO-d_6, 100 MHz)

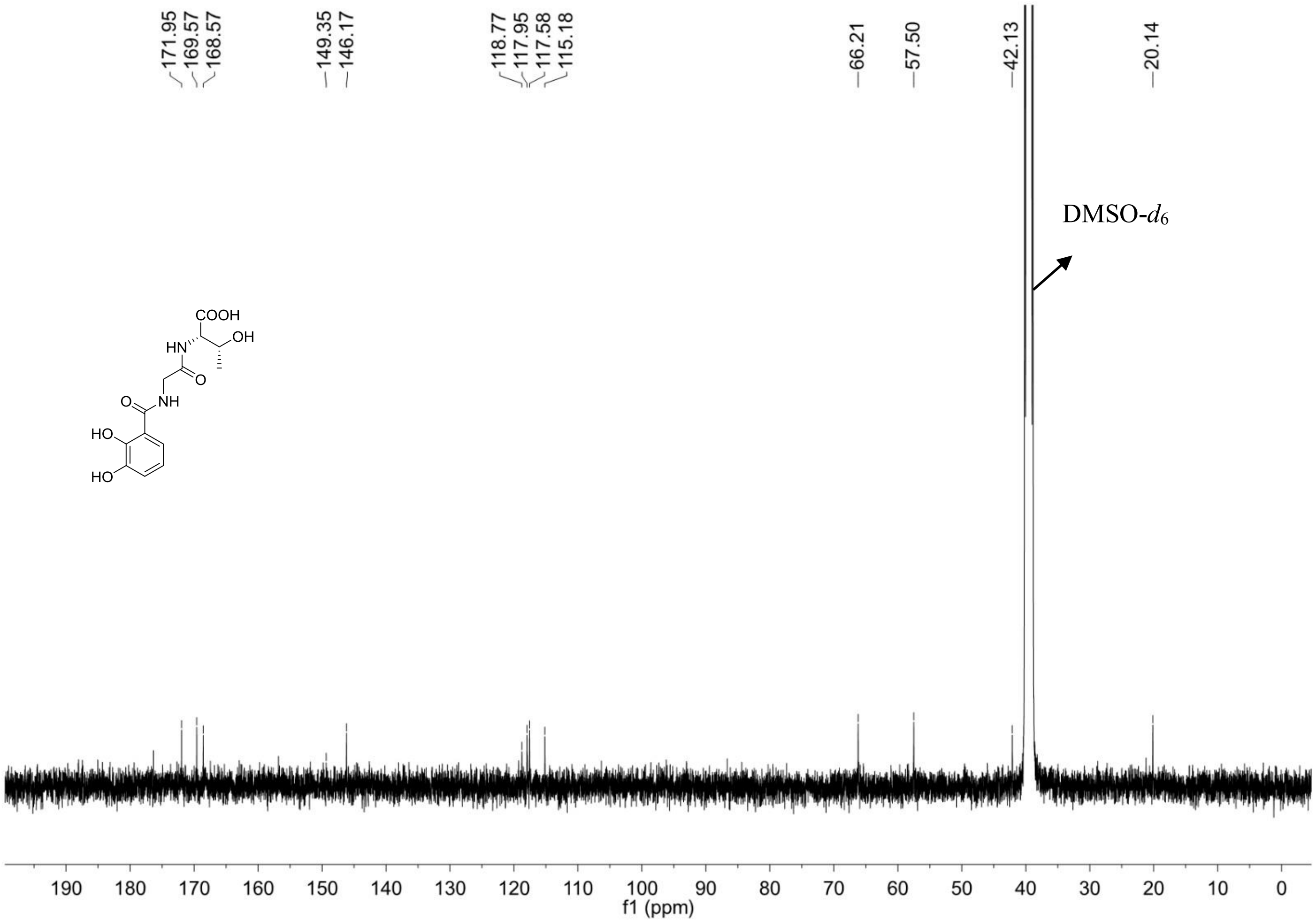

Figure S26. The HRESIMS spectrum of compound **4**.

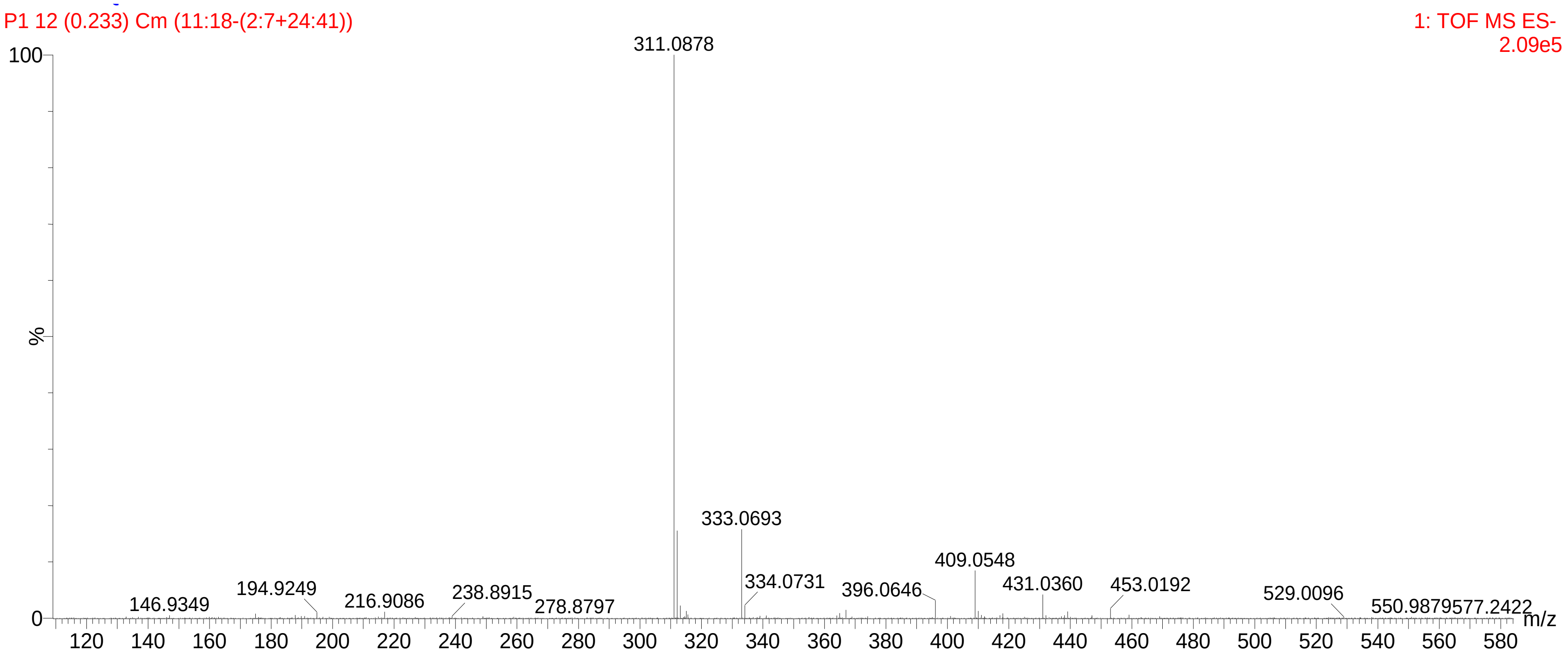

Figure S27. The ^{1}H NMR spectrum of compound **5** (pyridine-d_5, 400 MHz)

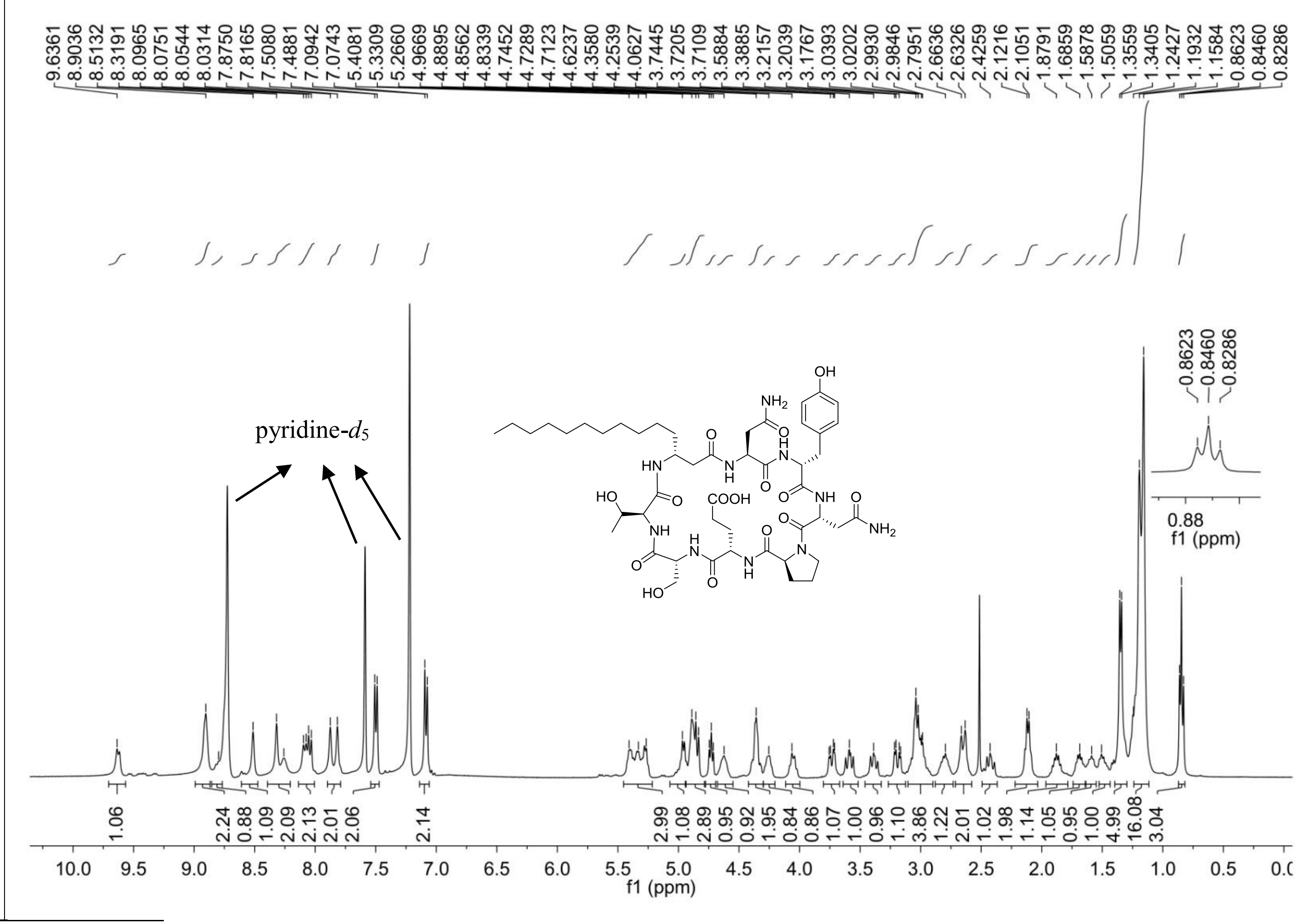

Figure S28. The COSY spectrum of compound **5** (pyridine-d_5, 400 MHz)

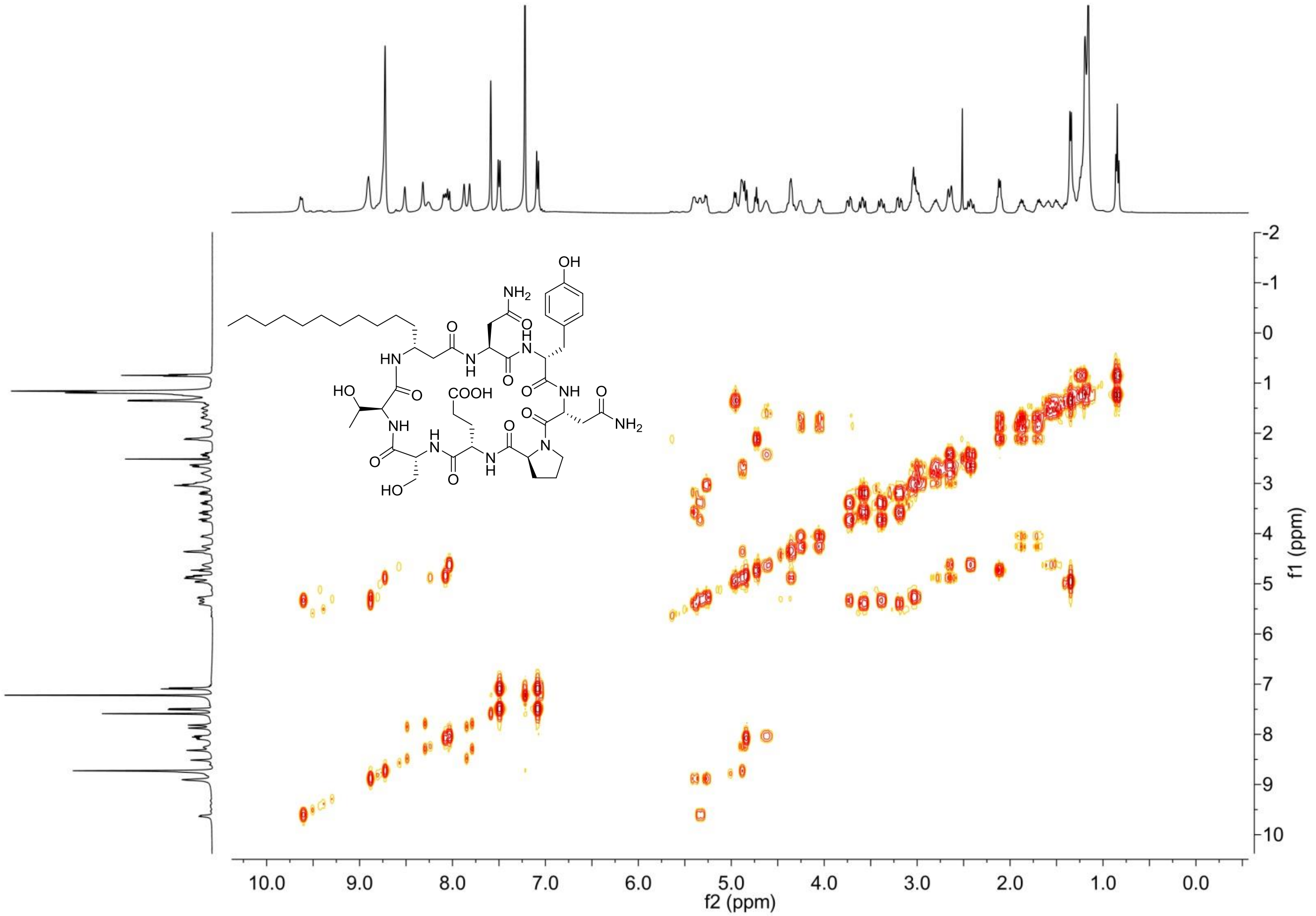

Figure S29. The ^{13}C NMR spectrum of compound **5** (pyridine-d_5, 100 MH)

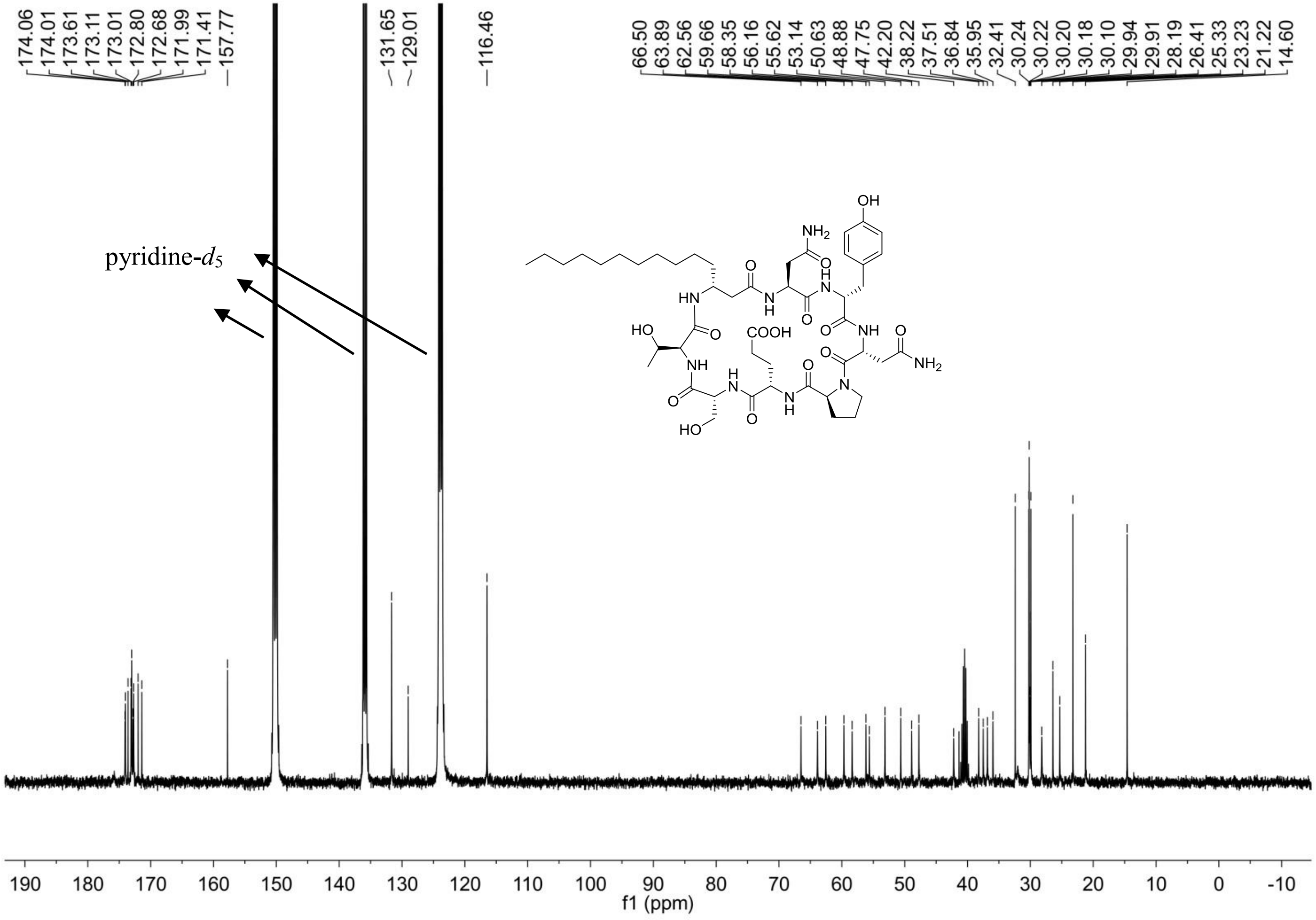

Figure S30. The HSQC spectrum of compound **5** (pyridine-d_5, 400 MHz)

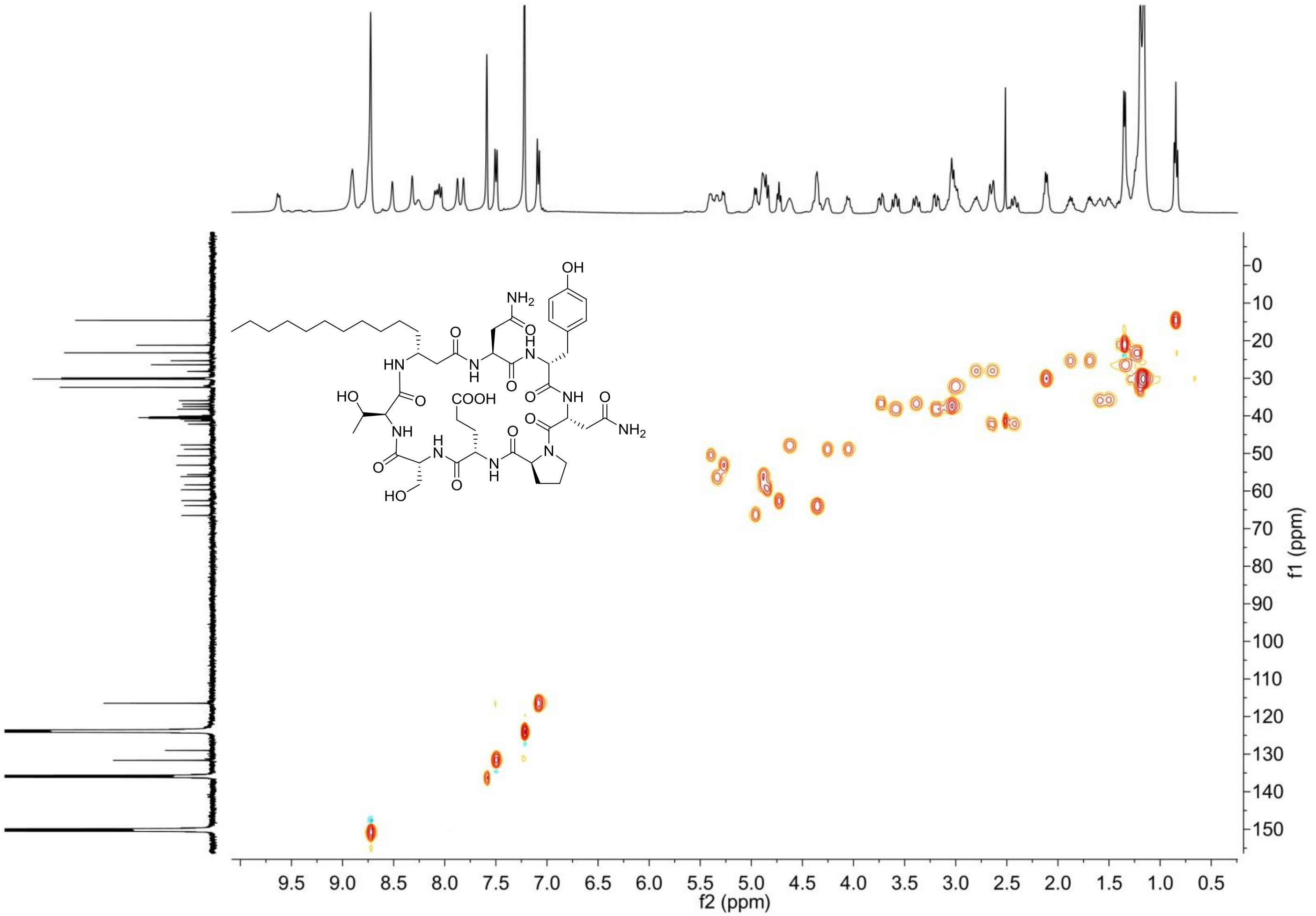

Figure S31. The HMBC spectrum of compound **5** (pyridine-d_5, 400 MHz)

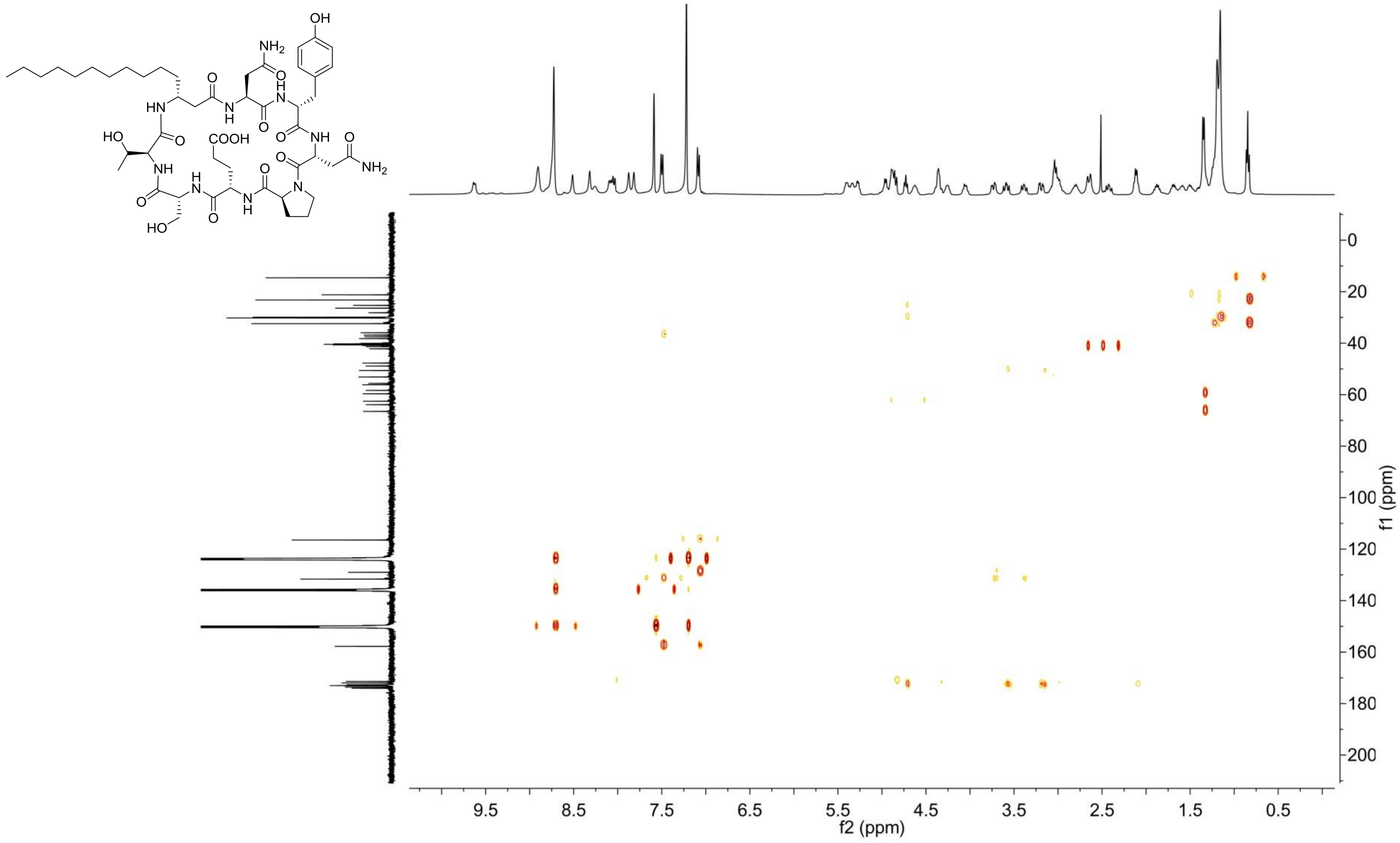

Figure S32. The ROESY spectrum of compound **5** (pyridine-d_5, 400 MHz).

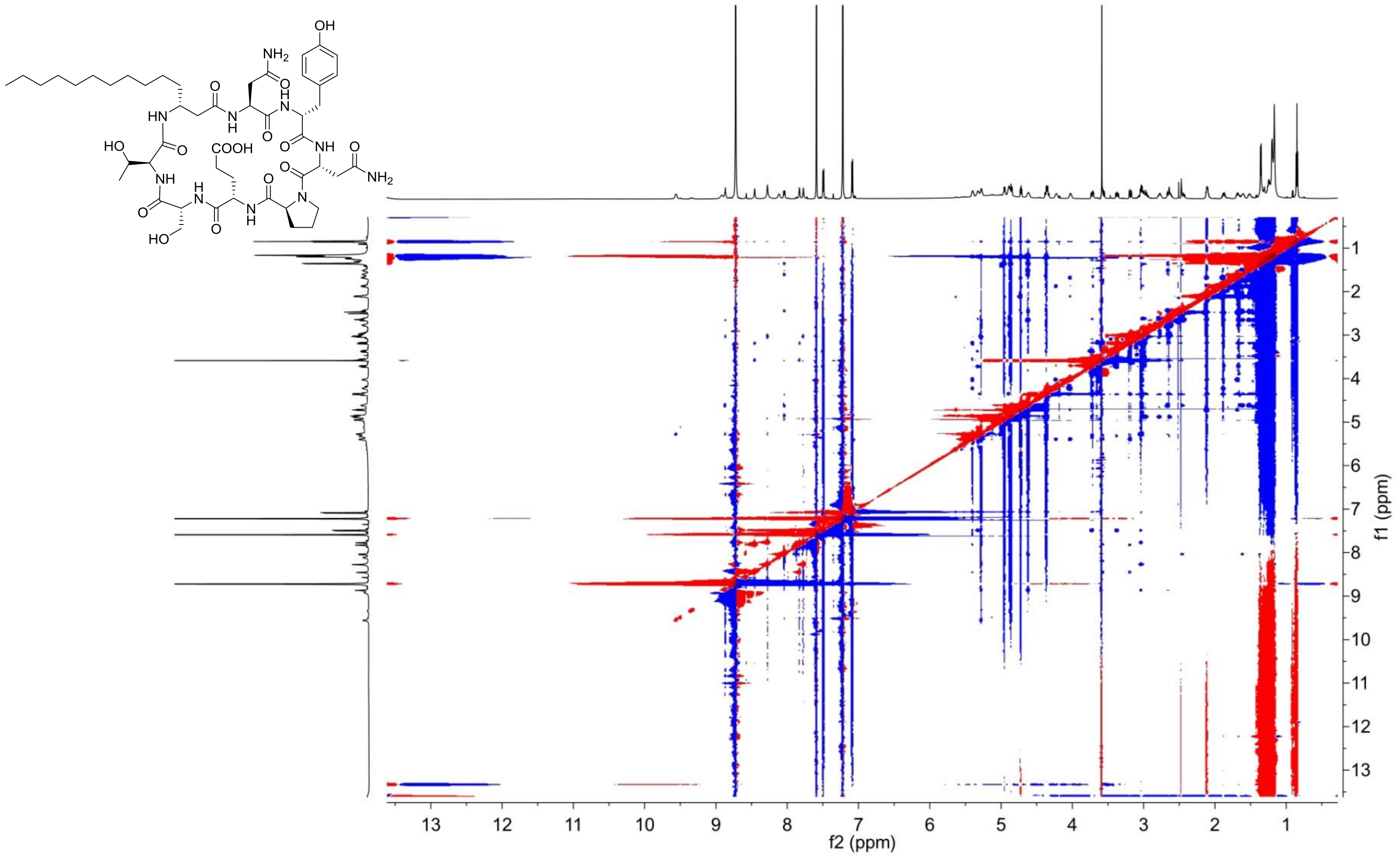

Figure S33. The HRESIMS spectrum of compound **5**.

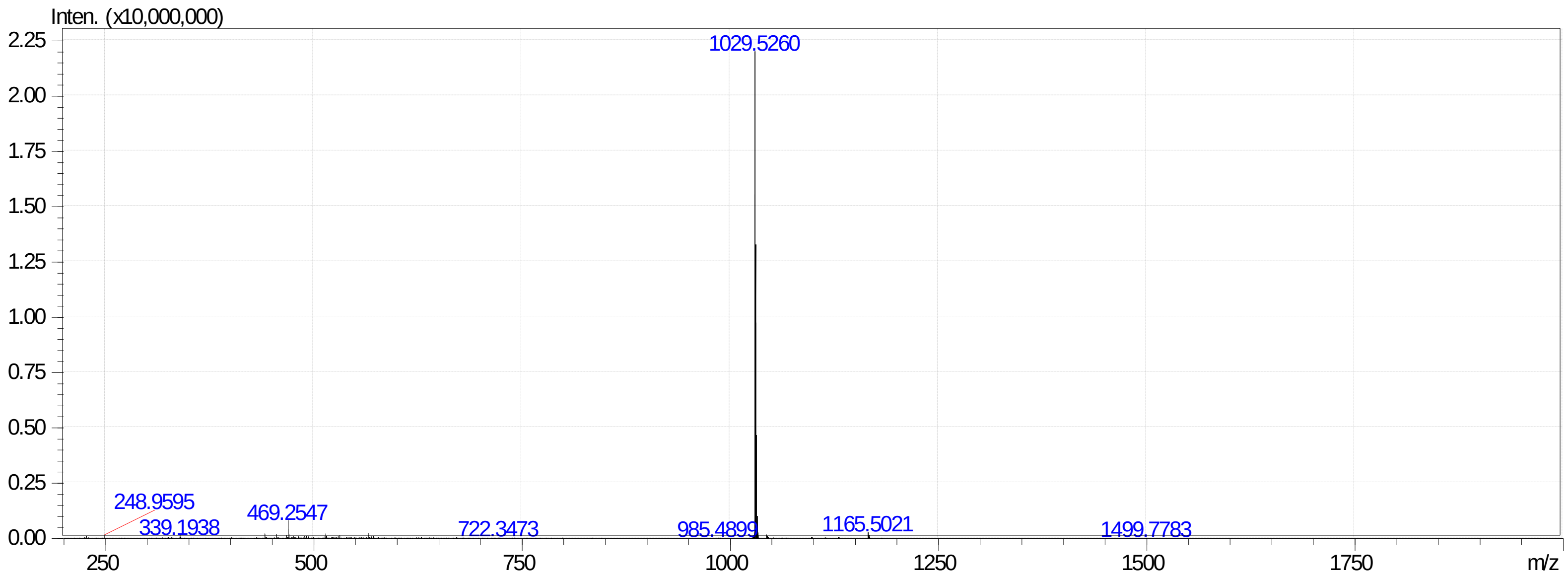

Figure S34. The ^{1}H NMR spectrum of compound **6** (pyridine-d_5, 400 MHz)

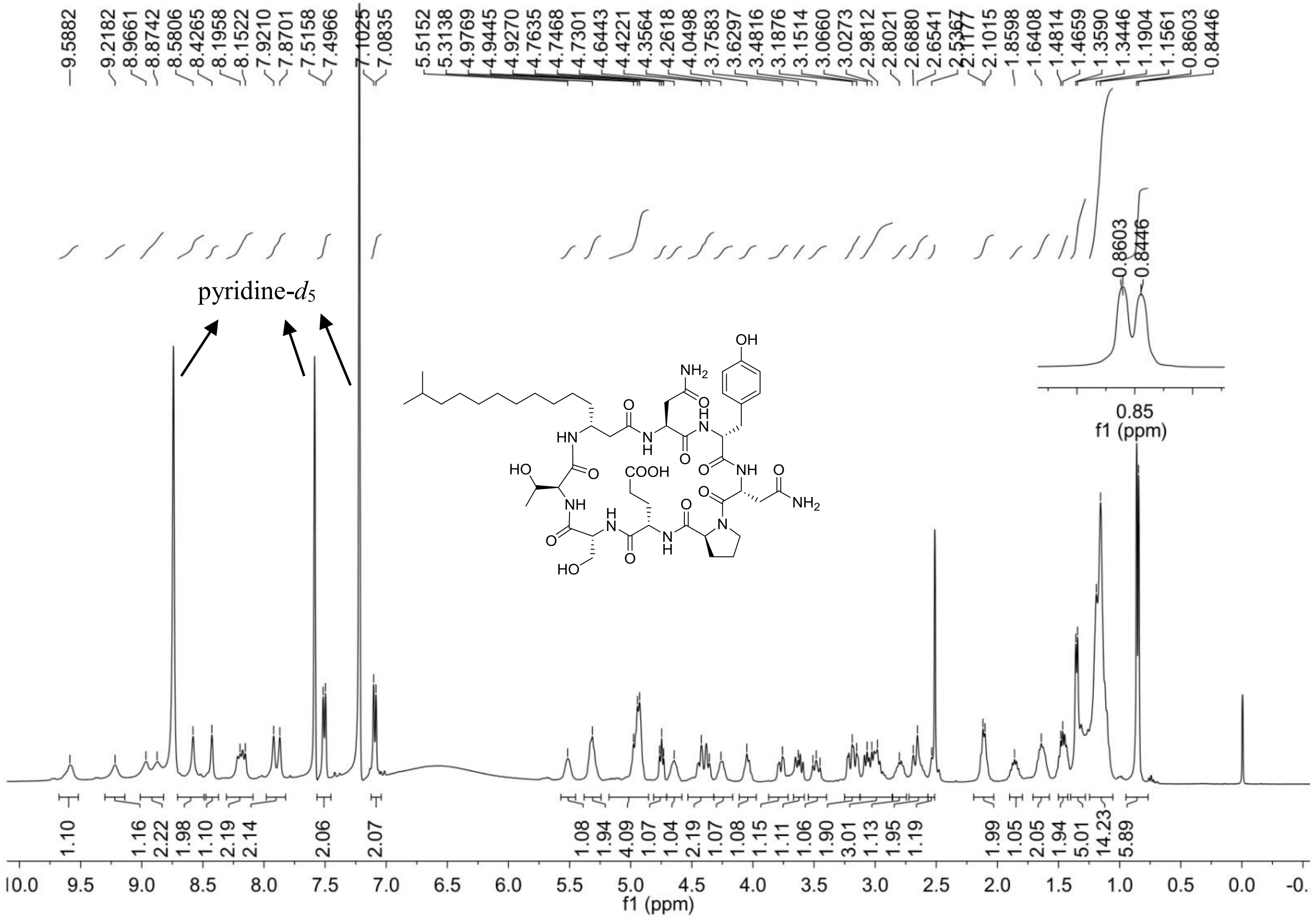

Figure S35. The ^{13}C NMR spectrum of compound **6** (pyridine-d_5, 100 MH)

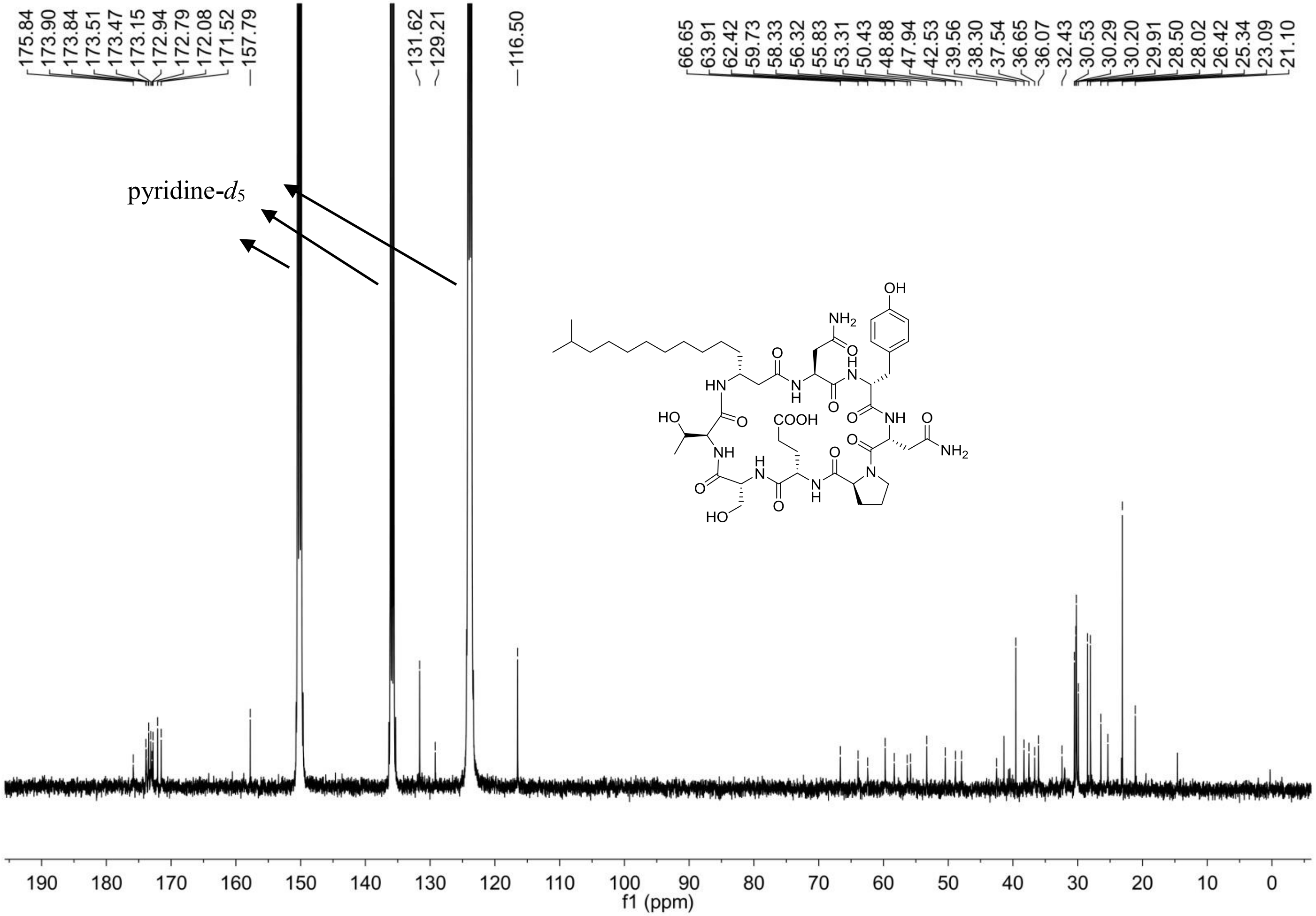

Figure S36. The HRESIMS spectrum of compound **6**.

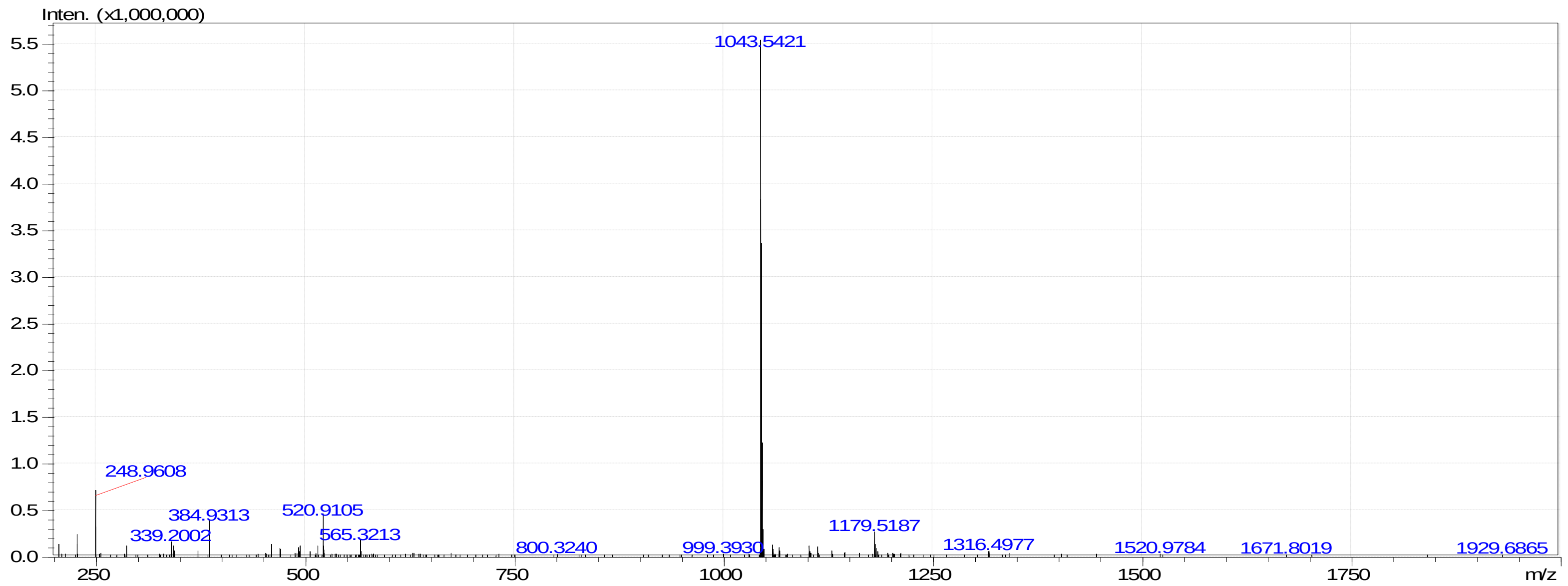

Figure S37. The ^{1}H NMR spectrum of compound **7** (pyridine-d_5, 400 MHz)

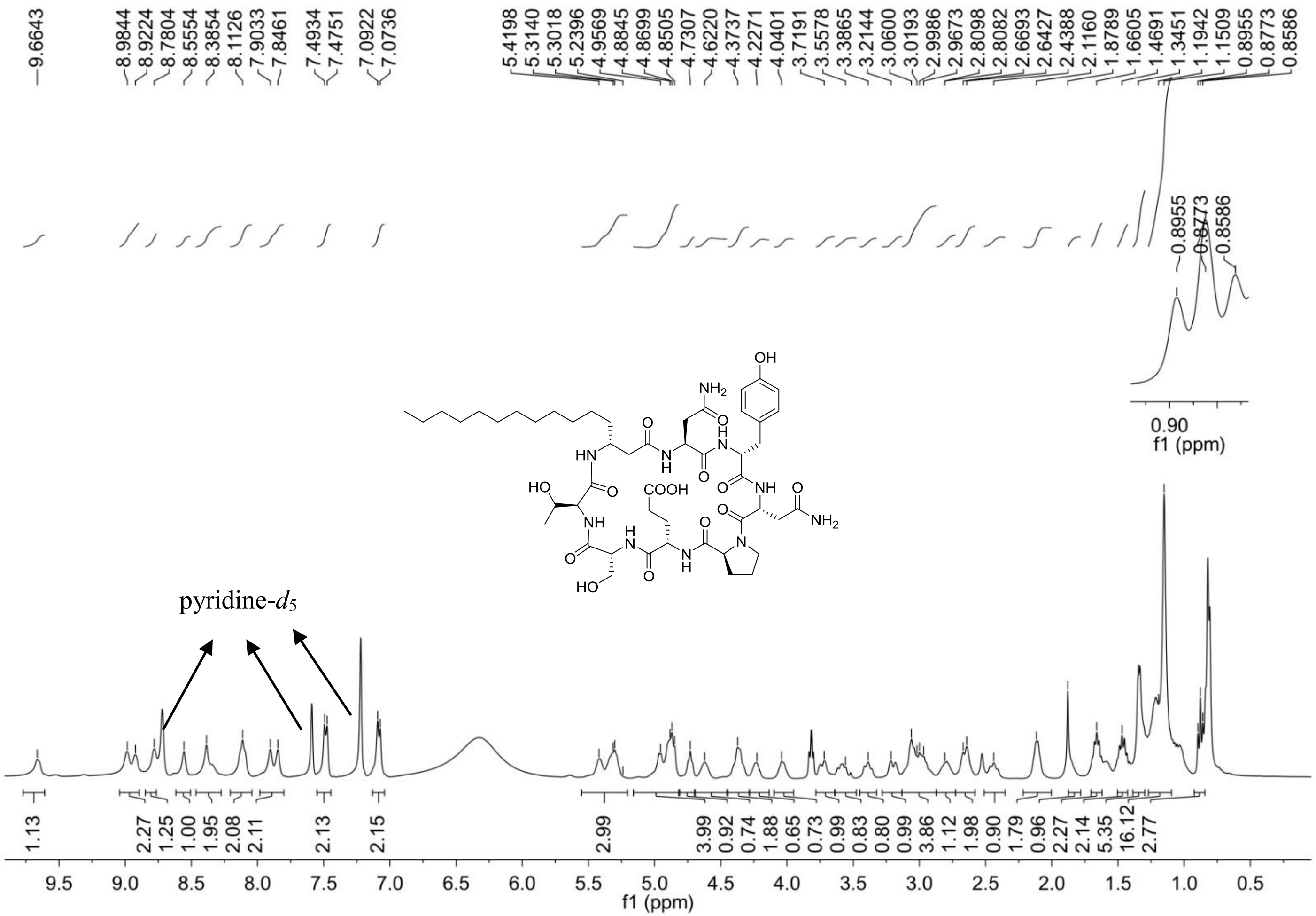

Figure S38. The ^{13}C NMR spectrum of compound **7** (pyridine-d_5, 100 MH)

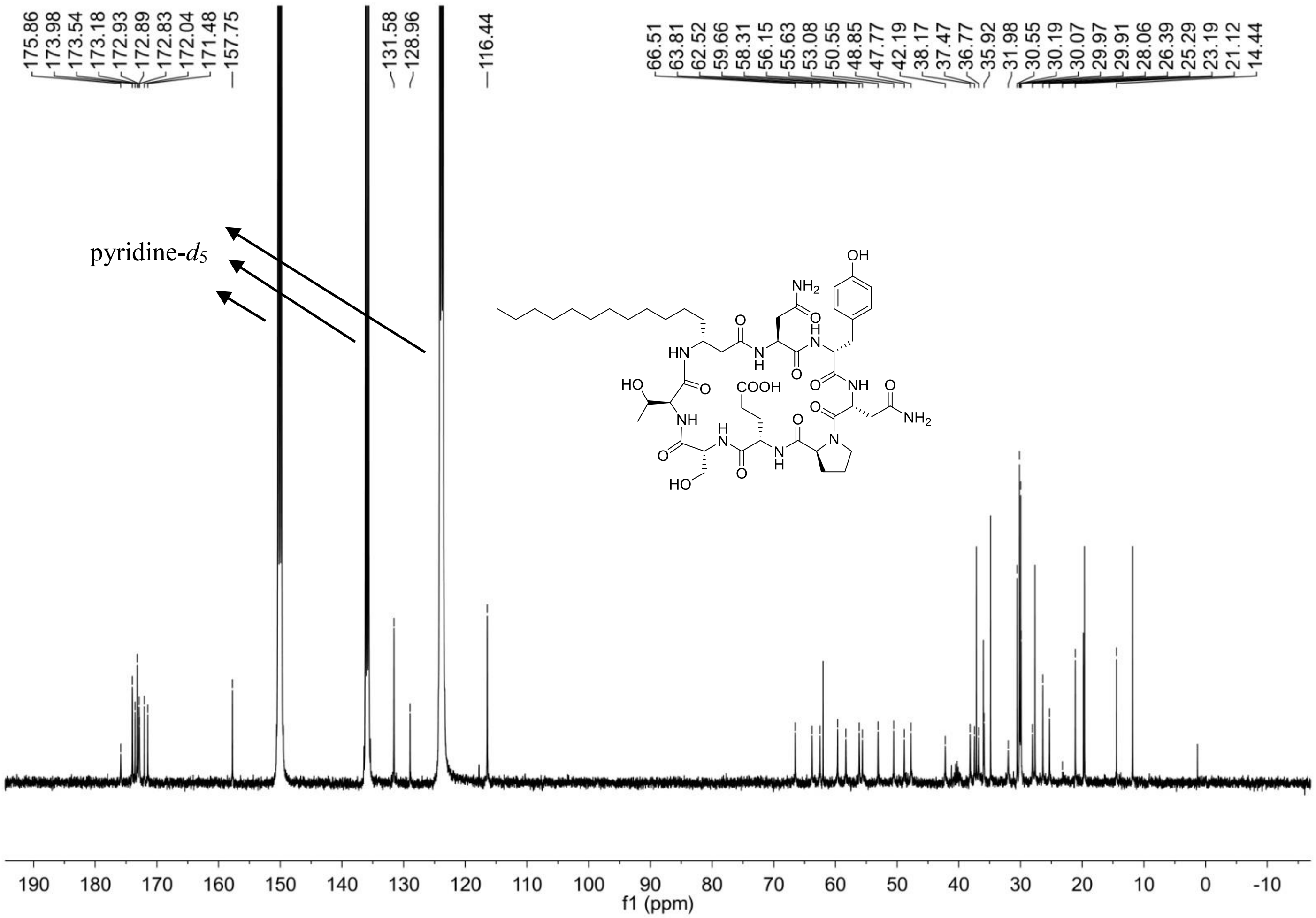

Figure S39. The HRESIMS spectrum of compound **7**.

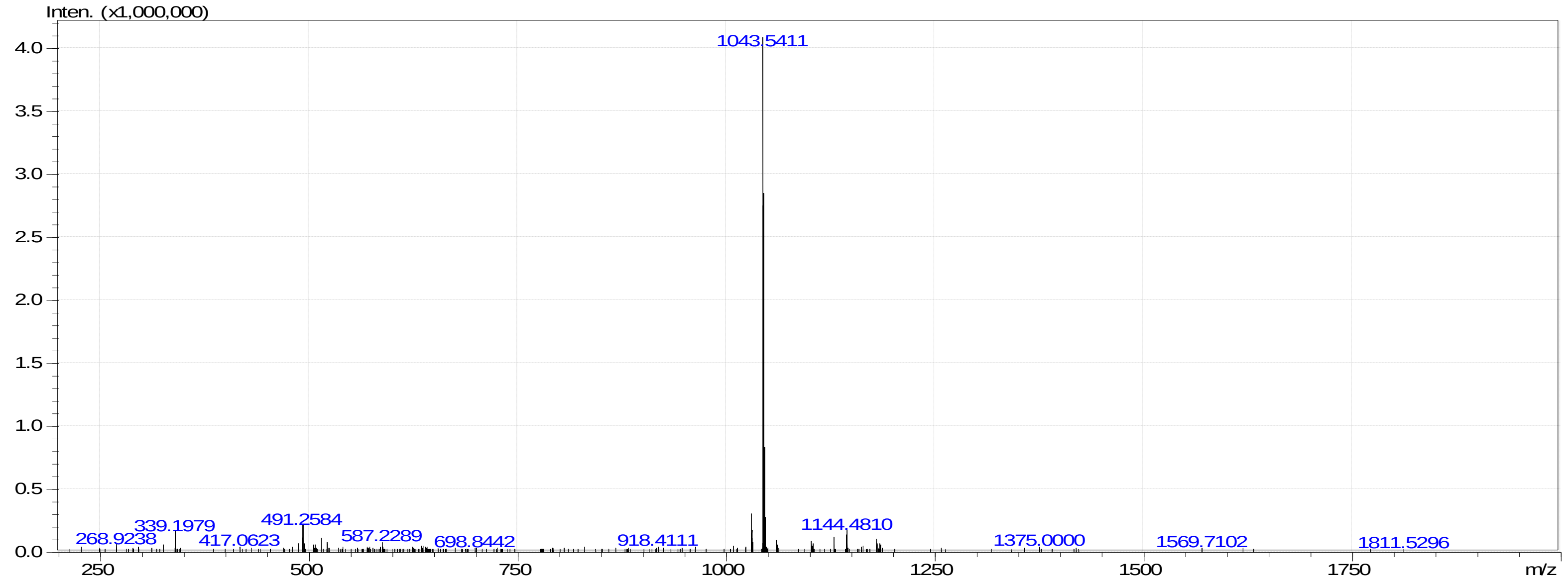

Figure S40. The ^{1}H NMR spectrum of compound **8** (pyridine-d_5, 400 MHz)

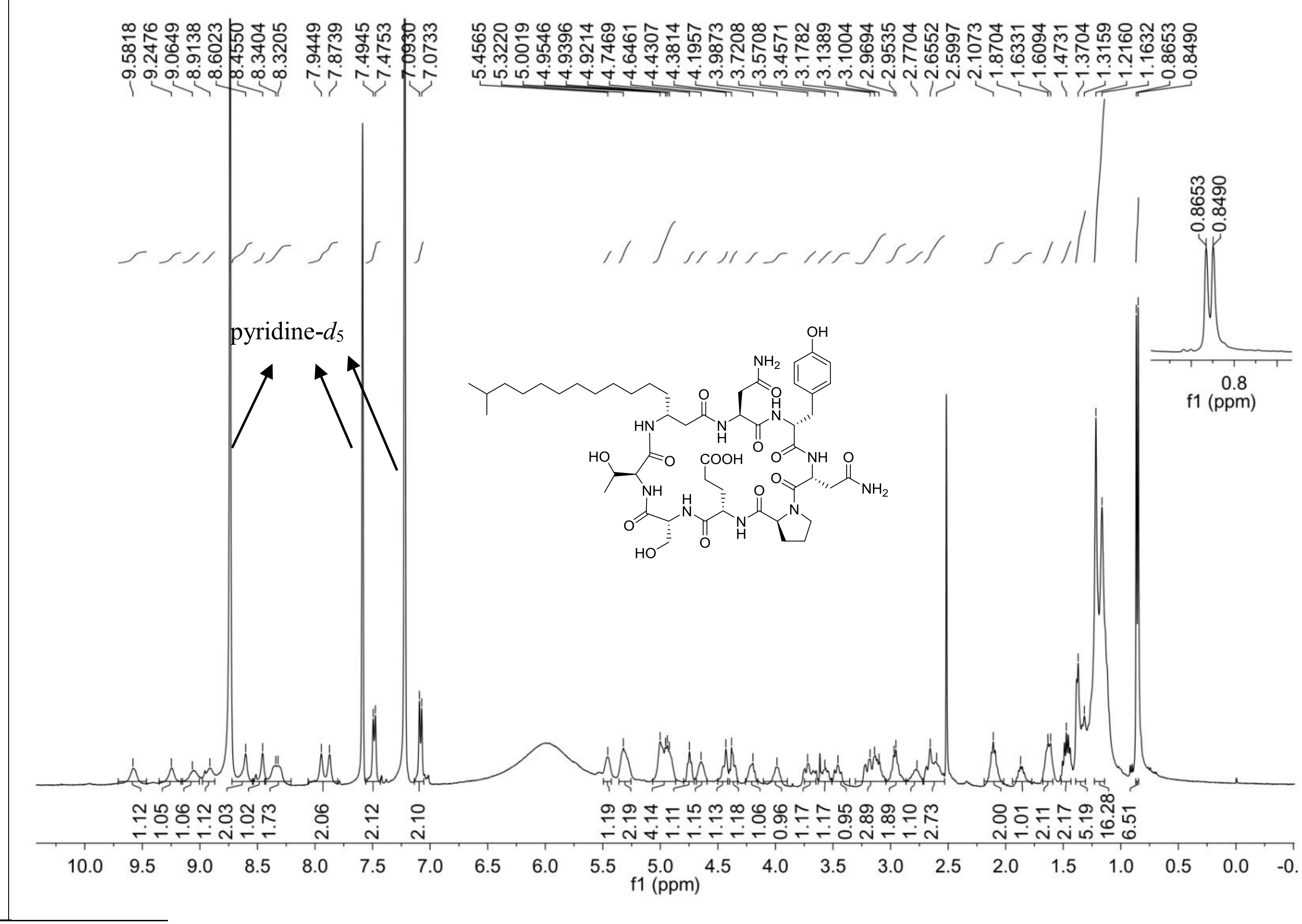

Figure S41. The ^{13}C NMR spectrum of compound **8** (pyridine-d_5, 100 MH)

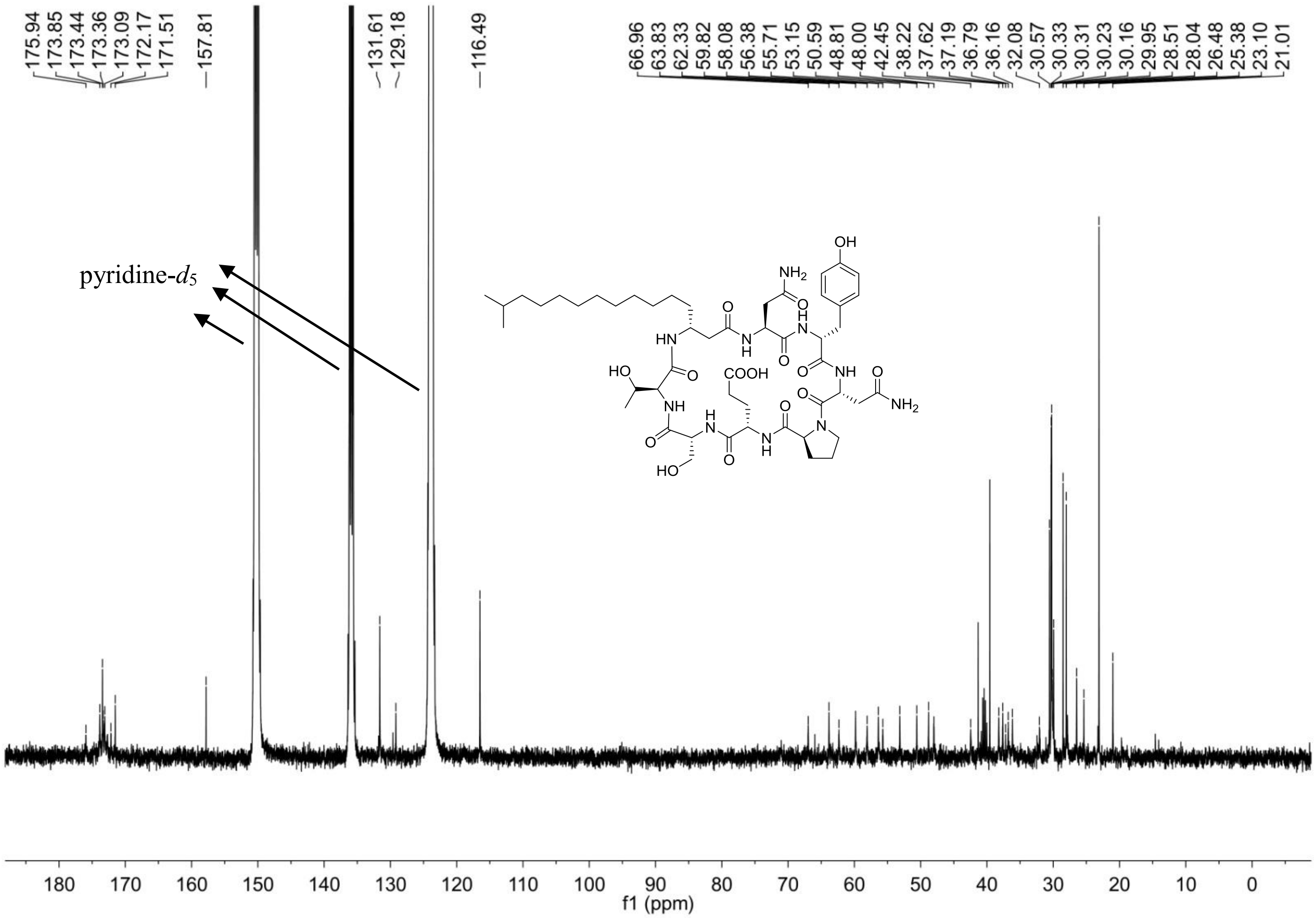

Figure S42. The ESIMS spectrum of compound **8**.

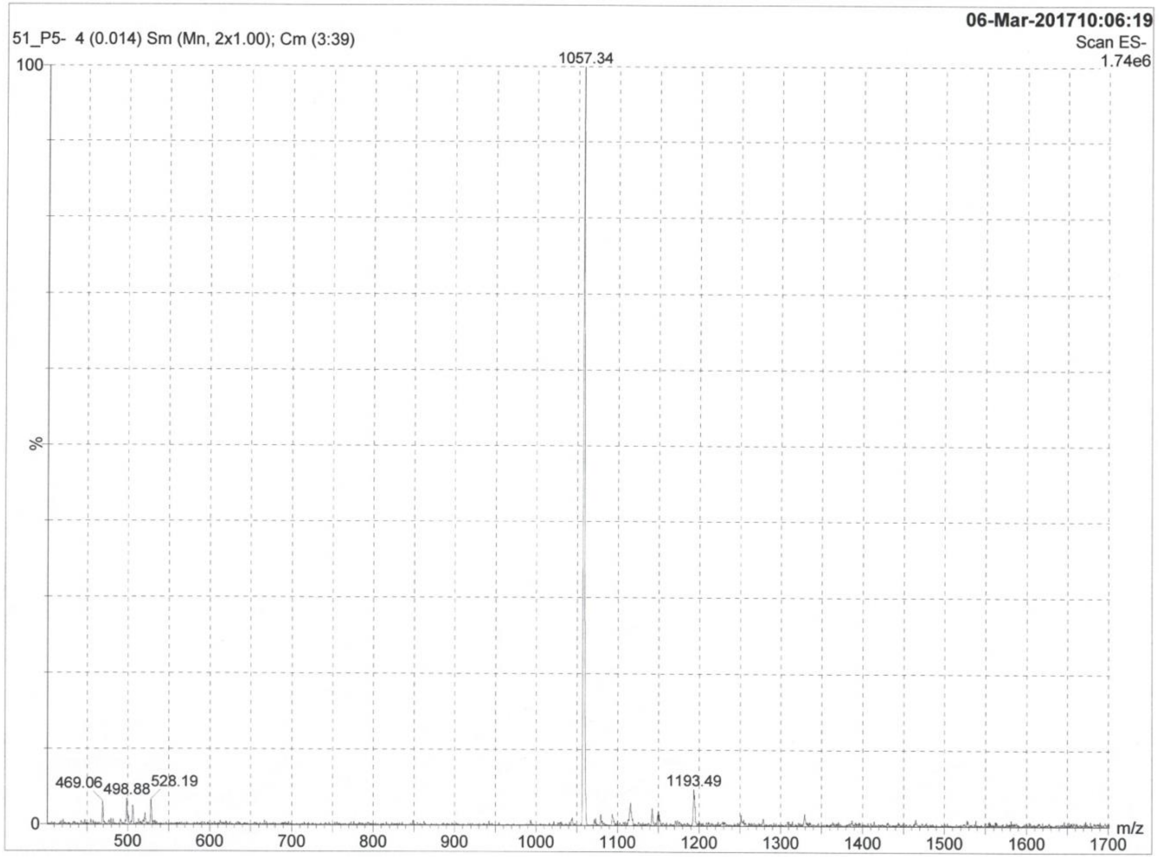

Figure S43. The ^{1}H NMR spectrum of compound **9** (pyridine-d_5, 400 MHz)

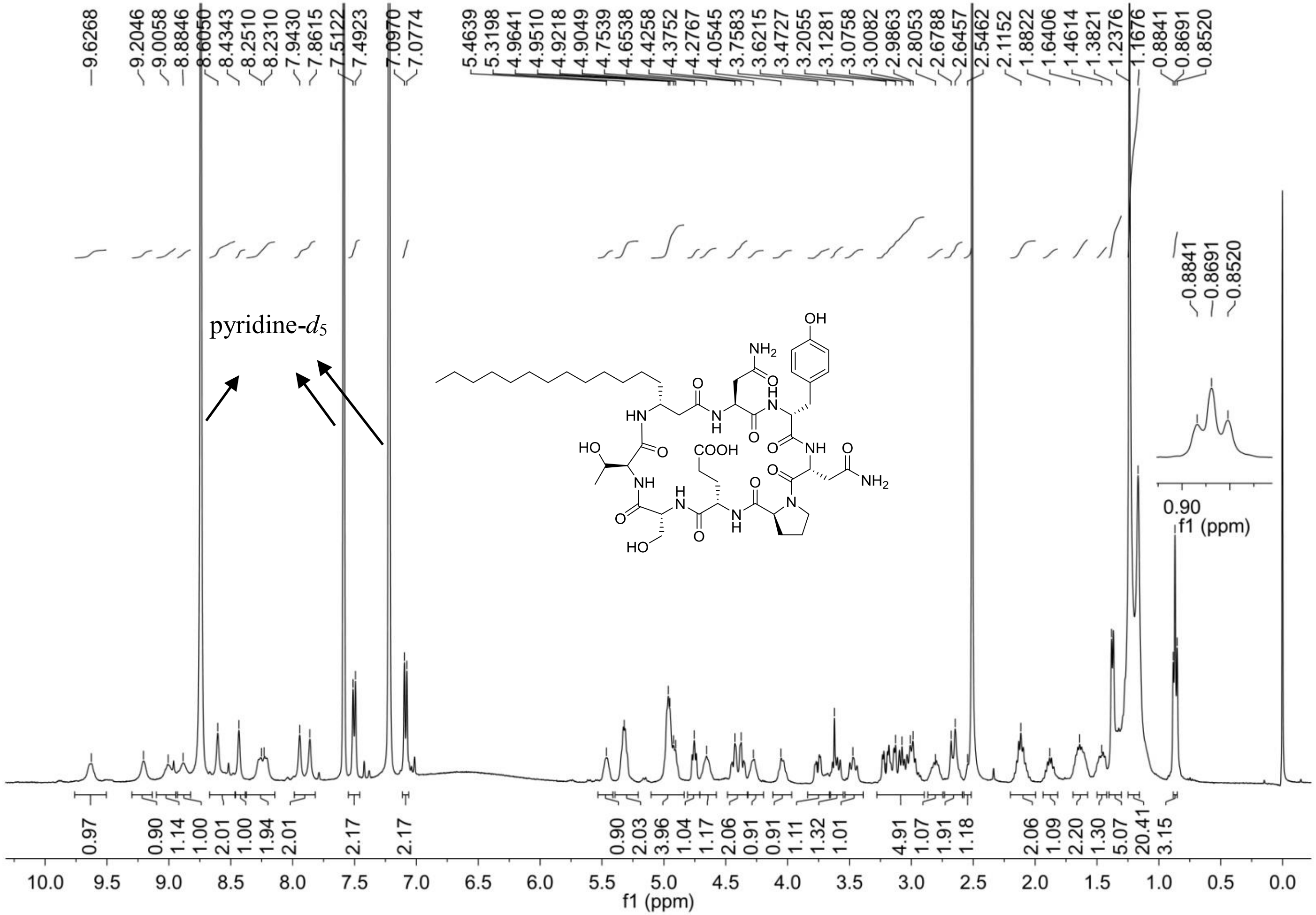

Figure S44. The ^{13}C NMR spectrum of compound **9** (pyridine-d_5, 100 MH)

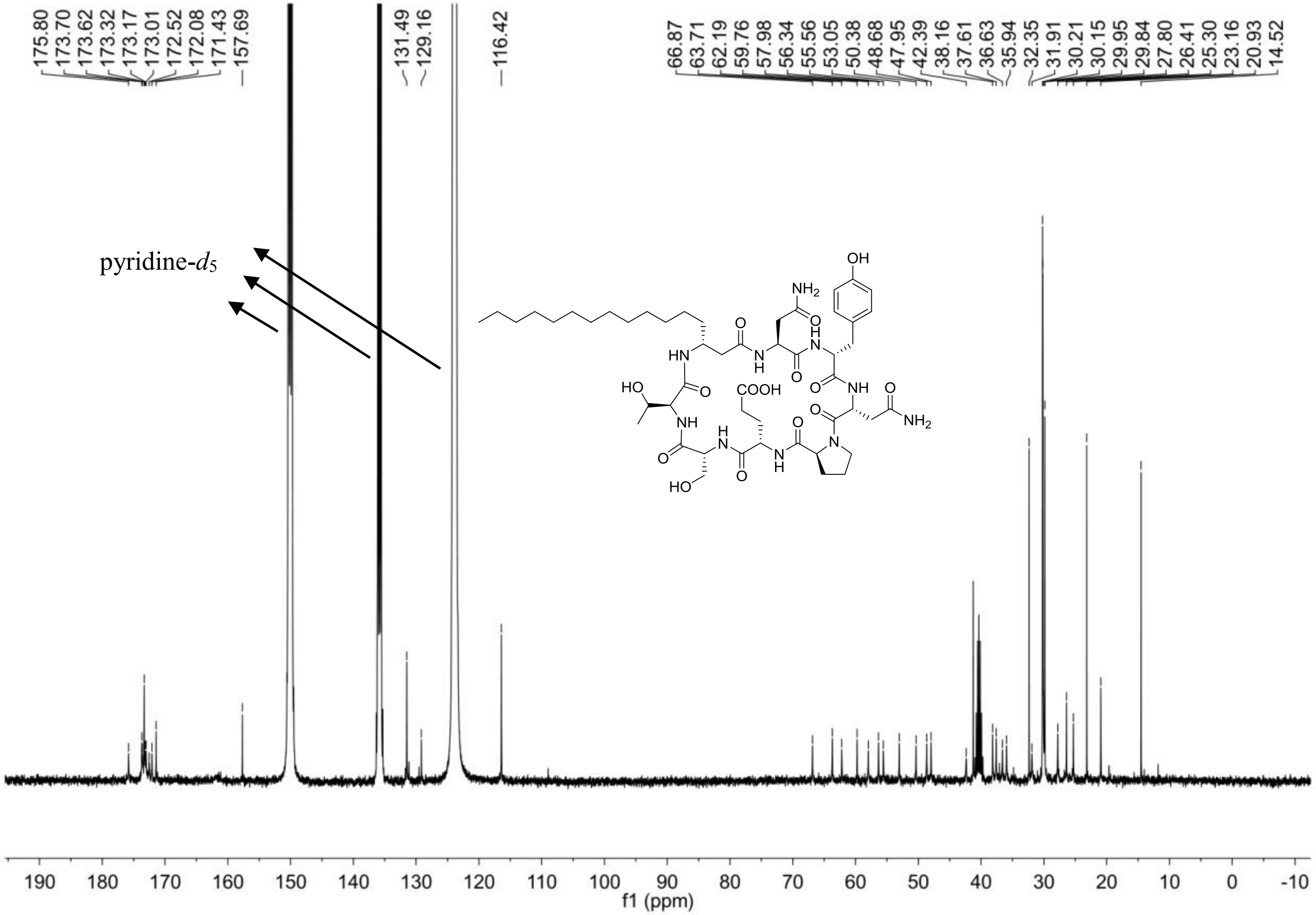

Figure S45. The ESIMS spectrum of compound **9**.

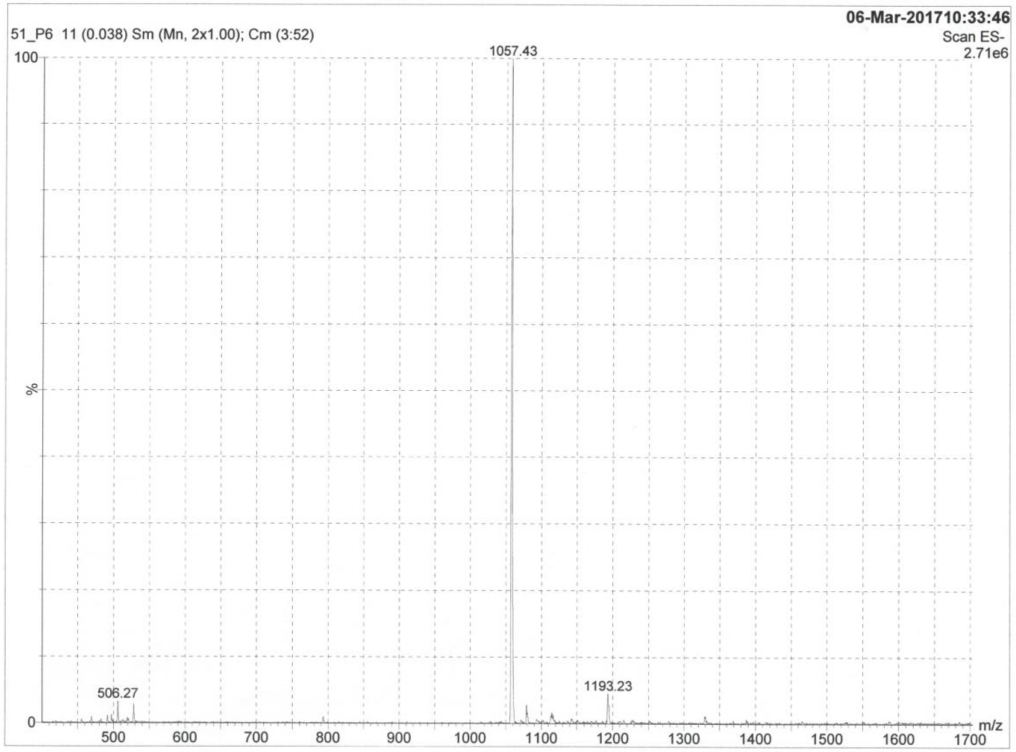

Figure S46. The ^{1}H NMR spectrum of compound **10** (pyridine-d_5, 400 MHz).

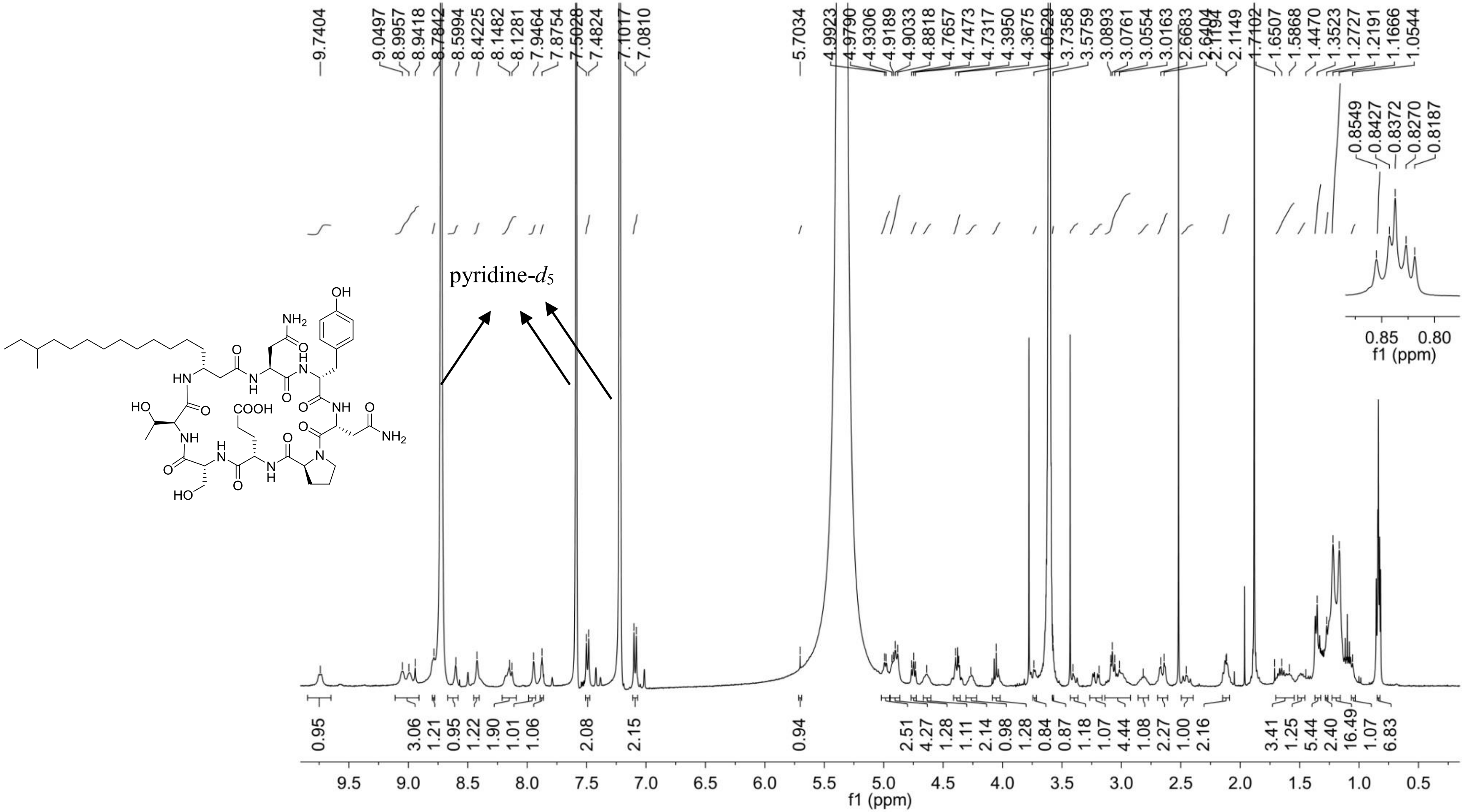

Figure S47. The COSY spectrum of compound **10** (pyridine-d_5, 400 MHz).

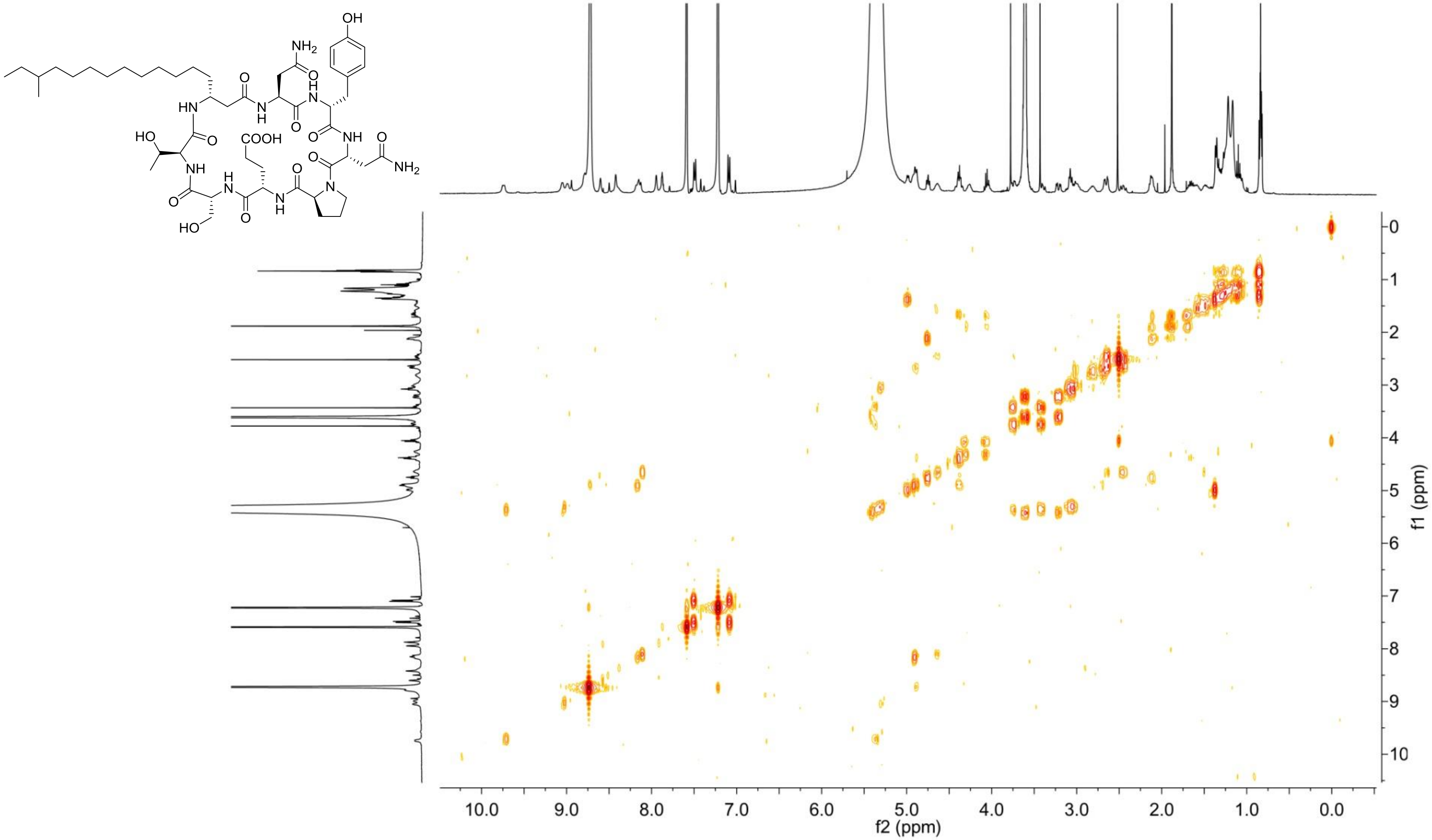

Figure S48. The ESIMS spectrum of compound **10**.

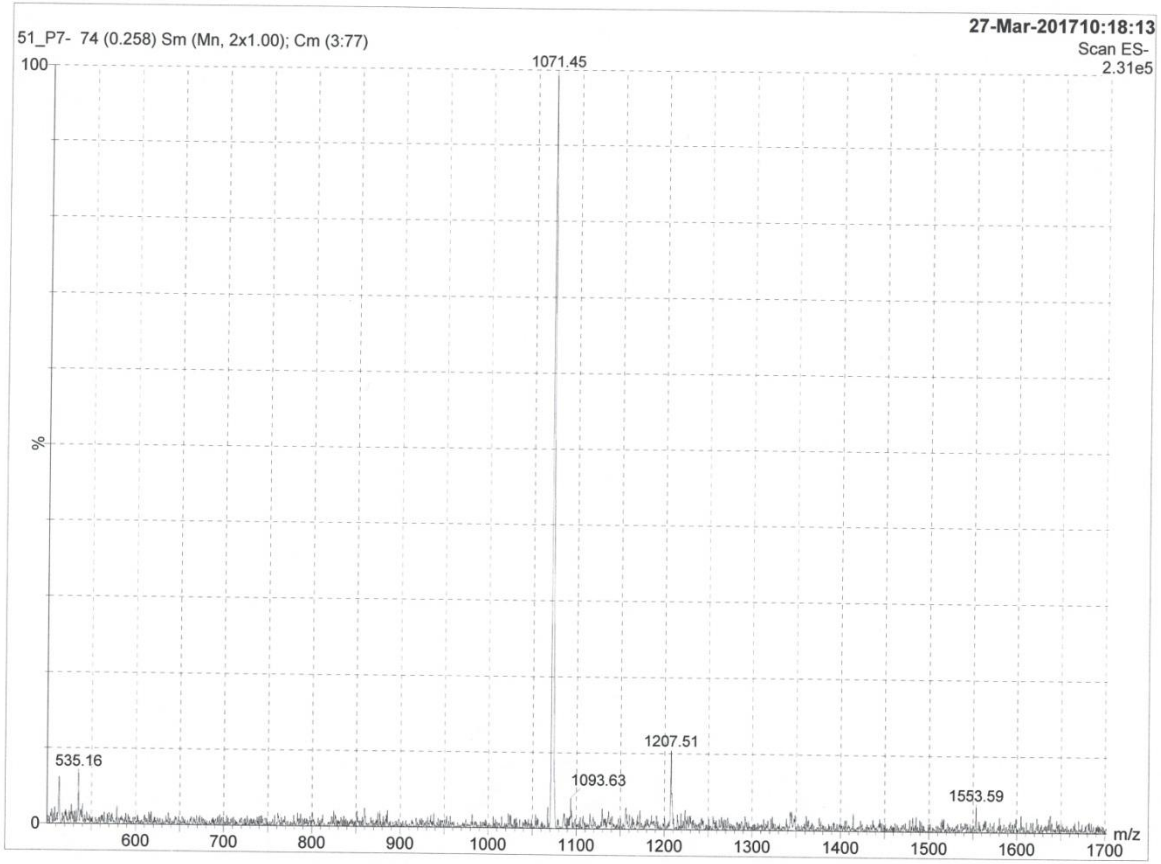

Figure S49. The Marfey's analysis of compound **5**. (I) The FDAA derivatives of hydrolysates of compound **5**. (II) The FDAA derivative of D-serine. (III) The FDAA derivative of L-serine. (IV) The FDAA derivative of D-threonine. (V) The FDAA derivative of L-threonine. (VI) The FDAA derivative of D-aspartic acid. (VII) The FDAA derivative of L-aspartic acid. (VIII) The FDAA derivative of D-glutamate. (IX) The FDAA derivative of L-glutamate. (X) The FDAA derivative of D-proline. (XI) The FDAA derivative of L-proline. (XII) The FDAA derivative of D-tyrosine. (XIII) The FDAA derivative of L- tyrosine.

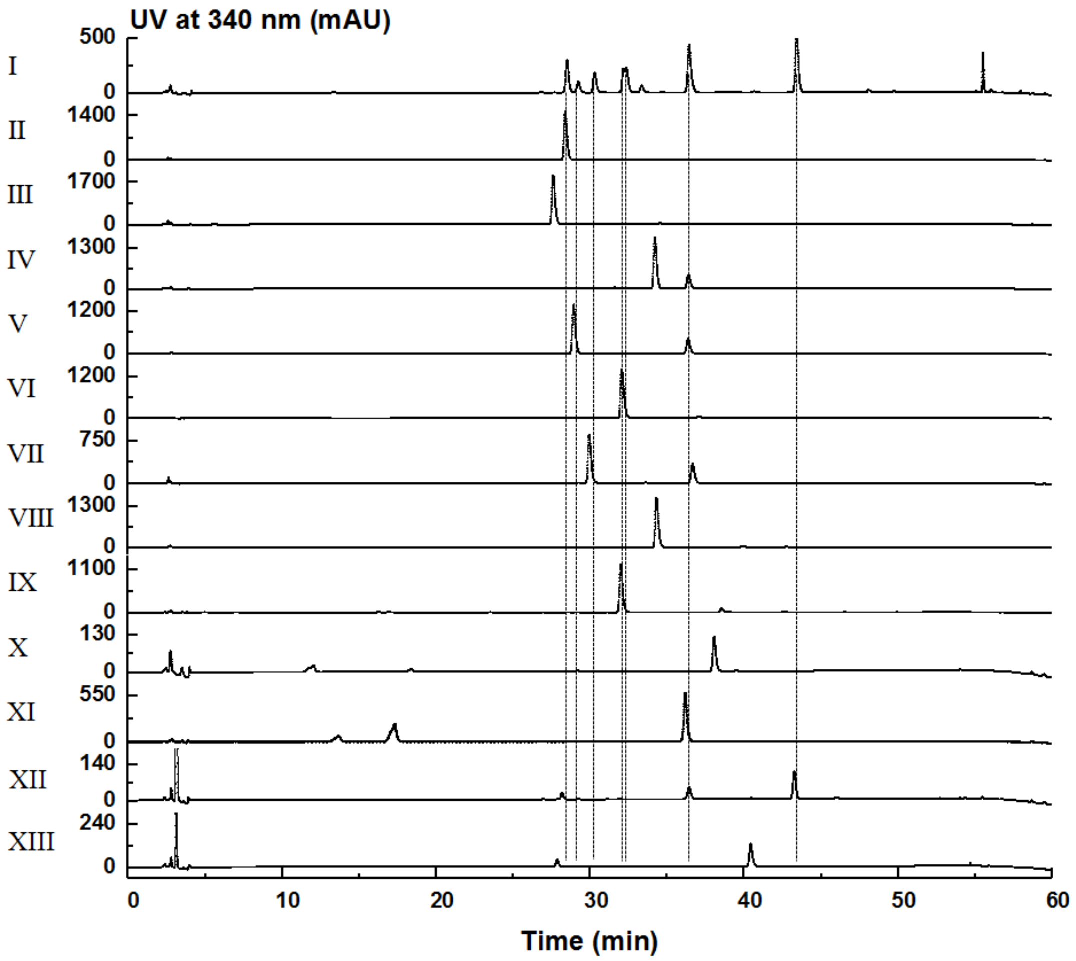

Supplement: Supplementary file 1 [file marinedrugs-16-00022-s001.pdf]
